# High-risk antimicrobial resistance genomic traits in multidrug-resistant *Salmonella* across China’s food chain: projections to 2050

**DOI:** 10.1038/s41467-026-78346-7

**Published:** 2026-10-20

**Authors:** Michelle Baker, Xin Lu, Chengchang Luo, Ge Wu, Alexandre Maciel-Guerra, Komkiew Pinpimai, Ming Luo, Mengyu Wang, Yuan Zhang, Weihua Meng, Baoli Zhu, Biao Kan, Tania Dottorini

**Affiliations:** 1 Faculty of Life Sciences & Medicine, School of Immunology and Microbial Sciences, Department of Infectious diseases, King’s College London, London, UK; 2 National Key Laboratory of Intelligent Tracking and Forecasting for Infectious Diseases, National Institute for Communicable Disease Control and Prevention, Chinese Center for Disease Control and Prevention, Beijing, China; 3 Centre for Smart Food Research, Nottingham Ningbo China Beacons of Excellence Research and Innovation Institute, University of Nottingham Ningbo China, Ningbo, China; 4 Faculty of Medicine and Health Sciences, Biodiscovery Institute, University of Nottingham, Nottingham, UK; 5 Yulin Center for Disease Control and Prevention, Yulin, China; 6 School of Public Health, Cheeloo College of Medicine, Shandong University, Jinan, China; 7 Key Laboratory of Pathogenic Microbiology and Immunology, Institute of Microbiology, Chinese Academy of Sciences, Beijing, China

**Keywords:** Machine learning, Bacterial infection, Antimicrobial resistance

## Abstract

Antimicrobial resistance (AMR) is rising globally, yet factors shaping its genomic evolution remain poorly understood. We investigated how socioeconomic, climate, and health factors are associated with AMR spread in multi-drug resistant (MDR) *Salmonella enterica* across China’s food chain, mapped hotspots and forecasted genomic trait prevalence to 2050. We analysed 2,536 MDR genomes from 15 provinces over 16 years, covering patients, food and non-food workers, meat, livestock, and environmental sources. A machine learning framework integrated genomic surveillance with risk assessment to identify high-risk AMR traits (clinically relevant, MDR, mobile). Regression models linked trait prevalence to socioeconomic, climate, health, and antimicrobial consumption data, informing projections to 2050. Eighty-four high-risk traits, including carbapenem, colistin, and β-lactam resistance, are projected to rise, with antibiotic use, population growth, healthcare spending, and urbanisation. Projections were trait-specific: *bla*_*CTX-M-64*_ and *bla*_*TEM-1*_ may increase up to 20%, while *bla*_*CTX-M-65*_ and *bla*_*CTX-M-14*_ remain below 5%. A mechanistic shift in fluoroquinolone resistance is projected: mutation-based resistance (*gyrA* 87) declines while plasmid-mediated genes (*qnrB17*, *qnrS1*) rise. Food workers emerged as a potential reservoir, showing higher trait prevalence than food sources, uniquely carrying last-resort antibiotic-resistance genes (*bla*_*TEM-30*_, *bla*_*NDM-5*_, *bla*_*CTX-M-64*_). Integrating genomic with socioeconomic surveillance could shift AMR interventions from reactive to preventive.

## Abstract (web summary)

A study of 2,536 MDR Salmonella genomes across China’s food chain forecasts resistance to 2050. Antibiotic use and population growth associate with rising carbapenem and colistin resistance, with food workers an overlooked last-resort ARG reservoir.

## Introduction

Antimicrobial resistance (AMR) is among the most pressing public health threats globally^1^, with an estimated 10 million deaths per year by 2050^2^. AMR emergence and spread is associated with a complex interplay of multidrug-resistant (MDR) genomic traits, mobile genetic elements (MGEs), cross-species transmission, and One Health interactions, all deeply influenced by socioeconomic, health and climate factors. These include antibiotic use, urbanisation, population density, climate, policy frameworks, healthcare practices and spending, WASH (water, sanitation, and hygiene) access, and income levels. Human behaviours, such as misuse of antibiotics for viral infections and alcohol use, alongside health conditions such as diabetes and obesity also heighten vulnerability to infections or reduce drug effectiveness. In Asia and, particularly in China, these dynamics are intensified by widespread antibiotic use in agriculture, rapid urban development, treatment practices, and seasonal climatic variability^3^.

The intersection of human, animal, environment and food, central to One Health and planetary health frameworks, is recognised as key to understanding the evolution and spread of AMR. This is reflected in the quadripartite collaboration between the World Health Organization (WHO), the World Organization for Animal Health, the Food and Agriculture Organisation of the United Nations (UN), and the UN Environment Programme, which together address human, animal, plant, food, water, and environmental health. Food plays a critical role in this framework as a reservoir for drug-resistant bacteria and AMR genes, with transmission occurring between hosts through direct and indirect routes, further amplified by global trade^4^. Globally, WHO estimates that unsafe food causes 600 million illnesses and 420,000 premature deaths each year, with low- and middle-income countries, especially young children, bearing the greatest burden^5^.

Among foodborne infections, *Salmonella* is responsible for an estimated 93.8 million cases of gastroenteritis and approximately 155,000 deaths per year globally^6^. Nontyphoidal *Salmonella* (NTS) are major contributors to foodborne illness, accounting for approximately 87% of all *Salmonella* infections globally^6^ and are among the most commonly reported causes of diarrhoeal diseases in China^7^, where *Salmonella* infections rank among the top two causes^7,8^ and NTS serovars are commonly reported causes^8^. How AMR spreads across the One Health domains of the food chain, particularly via major pathogens like *Salmonella enterica*, is poorly understood. Growing evidence supports that different factors, such as antibiotic usage, population growth, and income, are associated with AMR spread^9–12^. Building on this evidence, we have previously shown that in poultry farms and connected slaughterhouses in China, the gut microbiomes of livestock, workers and their households share similar clinically relevant antimicrobial resistance genes (ARGs) and similar associated mobile genetic elements^13–16^. Our previous recent surveillance study has begun to characterise the role of food workers as asymptomatic carriers of MDR *Salmonella*^17^. Notably, this landmark 8-year occupational health study of 260,315 workers in Yulin, China, confirmed that food workers carry *Salmonella* at significantly higher rates than non-food workers, with greater serovar diversity and higher levels of antimicrobial resistance, and identified plasmids as the primary vehicles of ARG dissemination^17^. However, that study was confined to a single city and two occupational groups, used phenotypic resistance data and basic genomic screening, and did not examine spread across the broader One Health food chain. Critically, it lacked a risk stratification framework, a population-structure-corrected analytical pipeline to disentangle lineage effects from true AMR associations, and any forecasting component to project future resistance trends. Building on this foundation, here we address these gaps by integrating high-resolution genomic surveillance of MDR *S. enterica* across the full One Health food chain, spanning patients, food and non-food workers, retail meat, livestock, and farm environments, across 15 Chinese provinces over 16 years, with national-level socioeconomic, climate, and health indicators, to identify the genomic traits and contextual factors associated to current and projected AMR trajectories to 2050. Here, by integrating high-resolution genomic data from MDR *S. enterica* with socioeconomic, health, and climate indicators we identify the factors correlated with AMR trait-specific projections across China’s One Health food chain domains, and forecast AMR trait prevalence to 2050.

## Results

### A wide array of *Salmonella* serovars with phylogenetic intermixing are spread throughout the food chain

To investigate AMR within the food chain (Fig. S1–S3) we analysed 2,536 whole genome sequences of MDR *S. enterica* isolates collected in China over a 16-year period. These isolates were collected from asymptomatic food and non-food workers, symptomatic patients, pork and chicken meat, livestock, and the livestock environment, Figure. S4. The dataset included 88 *S. enterica* serovars, with serovars *S*. Typhimurium monophasic variant 4,[5],12:i:- (19%) and *S*. Typhimurium (18%) being the most prevalent. Eighty different sequence types (STs) were found, with ST34 (22%) and ST19 (10%) being the most prevalent, in line with recent studies in China^18^, Figure. S4 and Supplementary Data 1. Serovar and ST were highly correlated (*V* = 0.85, *p-*value < 2.2 × 10⁻¹⁶, two-tailed Cramer’s *V* test).

To contextualise the genetic relatedness of MDR *S. enterica*, we also analysed 306 non-MDR isolates from the same sources (Fig. S4 and S5A and Supplementary Data 1). A maximum likelihood phylogenetic tree was constructed using both the MDR and non-MDR sequences, Fig. S5B. Clustering was observed by serovar, with *S*. Typhimurium and its monophasic variant intermixed. However, no relevant clustering was evident by source type, MDR status, geographic location, or collection year, except where these factors aligned with serovar.

### Antimicrobial resistance is disseminated throughout the food chain, via ARGs residing primarily in mobile elements

We identified 421 AMR traits, 88% of which were primarily located on mobile elements,either plasmids (ISs-Plasmid-ARGs and Plasmid-ARGs) or within ISs on the chromosome (ISs-ARGs),Fig. S6A and Supplementary Data 2. Among features present in multiple source types, ARG locations varied by source type (Kruskal-Wallis, two-tailed, *p*-value = 3.68 × 10^−^^15^). Forty of the 421 of the AMR traits were found in all seven source types (food workers, non-food workers, symptomatic patients, retail pork meat, and chicken meat, and chicken farms and their environments). Among these 40 AMR traits, aminoglycoside and cephalosporin resistance genes were the most prevalent, Fig. S6B–D and Supplementary Data 3. Notably, food workers had greater AMR feature richness than any other source type (Chao1 richness, with two-tailed Kruskal–Wallis test, *p*-value = 2.2 × 10⁻¹⁶), *post hoc* Mann Whitney pairwise tests with FDR correction *p*-values = 2.2 × 10⁻¹^6^).

Moreover, food workers not only showed higher trait richness but also uniquely carried 134 genomic features, including last-resort antibiotic-resistant AMR traits (conferring resistance to cephalosporins, carbapenems, and colistin, e.g., *bla*_CTX-M-64_, *bla*_TEM-30_, *bla*_NDM-5_, and *mcr-1.1*_IS*1A*), and these were primarily mobile (93.2%).

When examining shared traits between food workers and retail meat, we found differences between food worker-chicken and food worker-pork pairings, Figure. S7. Specifically, 30 AMR traits were shared between food worker and chicken isolates but absent in pork (3.3% Chr-ARG, 6.7% ISs-ARGs, 6.7% Plasmid-ARGs, 3.3% ISs-Plasmid-ARGs and 80% mixed across genomic locations), while 50 were shared between food worker isolates and pork isolates but absent in chicken (6% ISs-Plasmid-ARGs, 46% Plasmid-ARGs and 48% mixed across genomic locations). While 71 traits were common to food workers, pork, and chicken isolates (5.1% ARGs-Chr, 1.3% ISs-Plasmid-ARGs, 6.5% Plasmid-ARGs, and 79.2% mixed). The most prevalent resistance classes among these shared traits were aminoglycoside (31.1%) and cephalosporin (20.8%). Differences in the distribution of genomic features were observed between MDR and non-MDR isolates, see Fig. S8 and Supplementary Results.

In summary, a large proportion of AMR genomic traits were located on mobile elements, and many were found across more than one source type, with 40 traits shared across all sources.

### Genomic risk scoring reveals food workers and symptomatic humans (diarrhoea patients) carry the highest-risk AMR traits

**Fig. 1:**
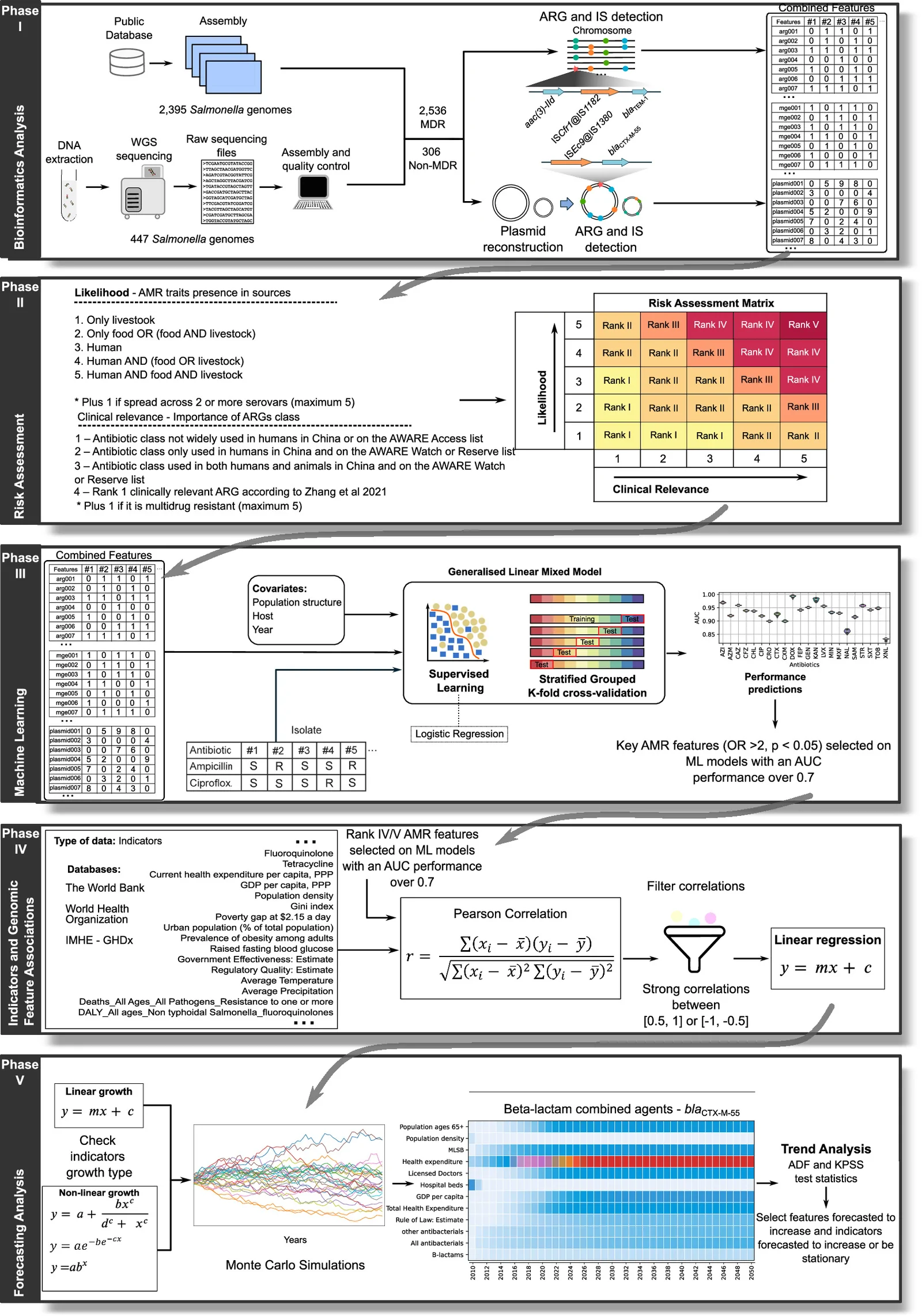
Data analysis workflow of the bespoke ML-based data mining and forecasting method. The workflow is divided into five phases: Phase I, Bioinformatic Analysis, Investigation of 2536 MDR and 306 non-MDR *Salmonella* isolates to identify all genomic AMR traits and their associated MGEs; Phase II, Risk Assessment, Application of the framework to classify AMR traits by their risk of spread, based on likelihood and clinical relevance; Phase III: Machine Learning, Training and testing of ML models to predict resistance profiles based on MDR genomes. Only models with high predictive power (>70% AUC) were retained to identify traits with strong genotype–phenotype associations. Phase IV: Indicators and Genomic Feature Associations, Correlating the prevalence of ML-selected, high-risk AMR traits with 96 socio-economic, environmental, and climate indicators across 15 provinces in China using Pearson correlation; Phase V: Forecasting Analysis, Development of forecasting models for AMR traits and indicators. Temporal trends were projected using regression, Monte Carlo simulations for uncertainty estimation, and statistical trend tests (Augmented Dickey-Fuller and Kwiatkowski-Phillips-Schmidt-Shin)^66,67^. Figure adapted from Baker et al. 2026^22^ published under CCBY 4.0 Attribution license (https://creativecommons.org/licenses/by/4.0/), changes were made to the figure.

Not all ARGs pose equal threats to public health; hence, we applied a risk assessment framework to identify high-risk traits integrating likelihood of spread and clinical relevance. High risk traits were defined by their capacity to spread across hosts and serovars, confer multidrug resistance, and include resistance to critically important antibiotics (based on their usage in humans and animals and AWARE status^19^); see Fig. 1 and Methods. High-risk AMR traits comprised Rank IV and Rank V risk classes, Fig. S9.

Across the full dataset of 2536 MDR *Salmonella enterica* genomes, 56.5% of the 421 AMR genomic traits were classified as high-risk, Supplementary Data 4 and Fig. S9. This included 95 AMR genomic traits conferring cephalosporin, carbapenem or colistin resistance. Some isolates harboured up to 24 high-risk traits, with the number of high-risk traits varying significantly by source type (Kruskal-Wallis, two-tailed, *p-*value = 3.657 × 10^−^^15^). Among the high-risk traits were ARGs such as *bla*_TEM-1_, associated with cephalosporin and penicillin resistance^20^, and *ermB* associated with streptogramin, lincosamide, and macrolide resistance^20^. Several variants conferring resistance to carbapenem, cephalosporin, or colistin were classified as high-risk (Rank IV and V). These included *mcr-9.1*, *bla*_TEM-135_, *bla*_CTX-M-3_, *bla*_CTX-M-27_, *bla*_TEM-238_, *bla*_OXA-10_, *bla*_TEM-150_, *bla*_CTX-M-123_, *bla*_NDM-5_, *bla*_CTX-M-65_, *bla*_CTX-M-14_, *bla*_NDM-1_, *bla*_CTX-M-55_, *bla*_OXA-1_, *mcr-1.1*, and *bla*_TEM-1_. In contrast, variants such as *bla*_DHA-1_, which showed limited spread across the food chain, were classified as medium-risk (Rank III), while *bla*_CTX-M-34_ (Rank II) and *bla*_NDM-9_ (Rank II), and *bla*_CTX-M-199_ (Rank II) were categorised as low risk.

Notably, 95% of all high-risk traits (Rank IV and V) occurred in food worker isolates, a significantly higher proportion than in food isolates (retail pork and chicken meat) (59%; *p*-value = 4.289 × 10⁻²¹, proportion test, two-tailed). As differences in sample size across source categories can affect the likelihood of observing rarer traits, we checked whether this difference was due only to rarer traits being found in the larger group. Firstly, assessing the proportion of high-risk AMR traits exclusively found in food workers vs those found in both food workers and food. Overall, only 35.5% of traits were found in food workers alone, compared to 64.5% that we also found in food and/or livestock, indicating that most high-risk traits were shared across food and/or livestock. Secondly, a bootstrapping analysis (10,000 iterations) in which food worker isolates were down-sampled to match the sample size of food isolates. This confirmed a smaller but statistically significant enrichment of high-risk traits in food worker isolates compared to food isolates after sample size correction (mean difference 7%; 95% CI [3.4%, 11.3%], one-tailed bootstrap *p*-value = 0.0004), indicating that while the magnitude of the difference was in part inflated by sample size, the direction and significance of the enrichment reflect a true biological difference between groups.

To assess whether this enrichment might reflect a systematic bias in the risk assessment framework toward human-derived isolates more broadly, or broader human-associated AMR patterns rather than food worker-specific characteristics, we applied the same bootstrapped down-sampling analysis to the two other human source groups in our dataset. Non-food worker isolates, recruited from the same Guangxi Province under the same occupational health surveillance programme as food workers, providing the strongest available control for both human-source effects and regional epidemiological differences, showed a slightly higher proportion of high-risk traits than food isolates after sample size correction (mean difference 4.3%; 95% CI [1.3%, 7.1%], one-tailed bootstrap *p*-value = 0.002), but with a smaller effect size than food workers. Symptomatic patient isolates showed a significantly lower proportion of high-risk traits than food isolates after sample size correction (mean difference −5.9%; 95% CI [−9.2%, −2.5%], one-tailed bootstrap *p*-value = 0.0002). We note that the symptomatic patient comparison is weaker evidence than the non-food worker comparison, as symptomatic patients differ from food workers in province, collection period, and health/exposure status simultaneously, whereas non-food workers differ only in occupational food contact within an otherwise matched sampling frame. As a further test, food worker isolates were directly compared to non-food worker isolates (all collected as part of the same surveillance programme), removing confounding factors of host, geography, collection and years. This showed that after down-sampling, although not statistically significant, food workers had a slightly higher proportion of high-risk AMR traits compared to non-food workers (mean difference 2.35%, CI [−1.68%, 6.3%], one-tailed bootstrap *p*-value = 0.31).

The gradient of effect sizes across human source groups, food workers 7% > non-food workers 4.3% > symptomatic patients −5.9%, is inconsistent with a uniform artificial inflation of risk scores across human sources and instead supports a genuine occupational exposure gradient specific to food-related work. Nevertheless, as food workers and food isolates were derived from different provinces, residual geographic confounding cannot be fully excluded (see limitations in the Discussion), and the enrichment should be interpreted as an epidemiological association rather than a causally attributed source effect.

As an additional assessment the decomposed risk score was also analysed, specifically considering the risk relevance only and disregarding the risk likelihood that contained the mobility component, which was host dependent. Considering AMR traits with a risk relevance score of 4 or 5 (which included only Rank I clinically significant traits and MDR traits conferring resistance to antibiotics used in both animals and humans and on the AWARE Watch or Reserve lists) revealed similar results to the previous analysis. Specifically, food worker isolates vs food isolates: mean difference +7.1% (95% CI [2.8%, 11.3%], one-tailed bootstrap *p*-value = 0.0006); non-food worker isolates vs food isolates: mean difference +5.0% (95% CI [2.43%, 7.7%], one-tailed bootstrap *p*-value = 0.0002); Symptomatic patient isolates vs food isolates: mean difference −5.9% (95% CI [−8.9%, −2.8%], one-tailed bootstrap *p*-value = 0.0001).

Thirty-three traits were classified as Rank V high-risk, of which 31 were found in food workers and eight in isolates from both food workers and symptomatic patients. Together, these findings suggest that food workers may serve as a potential reservoir and hotspot for AMR transmission, though causal directionality cannot be inferred from cross-sectional data alone.

### Machine learning predicts that high-risk AMR traits are strong predictors of observed AMR resistance/susceptibility profiles

Beyond mapping the spread of AMR traits across the food chain, we sought to identify genomic traits that are not only present across all sources but are also strongly predictive of observed resistance profiles, reinforcing their functional relevance. We applied a Bayesian mixed-effects logistic regression approach to determine which AMR traits were associated with the observed resistant or susceptible profiles from antibiotic susceptibility testing to 37 antibiotics, adapted from previously described methods^15^, Fig. 1 and S2. The analysis was conducted across all available isolates spanning different lineages (PopPUNK clusters, see Methods), provinces, years, and source types, which were included as random effects to account for lineage and sampling dependence. For each isolate, AMR genomic traits were encoded as input features for the ML models (see Methods).

Following training and validation, 18 antibiotic-specific models demonstrated strong predictive performance, each with an AUC > 0.7, Fig. S10 and Supplementary Data 5. Interclass correlation coefficients (ICCs) were calculated for each model to assess the model variance attributable to each random effect. On average across the 18 models, ICCs were 0.074 (lineage), 0.149 (province), 0.164 (year), and 0.123 (source type), indicating that all four random effects contributed meaningful variance. Province ICC (0.149) and year ICC (0.164) were the largest contributors, this shows that temporal and geographic variation are the strongest confounders.

The most important AMR traits were selected from the accepted models (AUC > 0.7) on the basis of three criteria: an effect size (odds ratio, OR) ≥ 2 in the final fitted model; statistical significance defined as a lower 95% OR confidence interval ≥ 1 (equivalent to a *p*-value ≤ 0.05); and cross validation stability defined as a positive association with resistance across 80% of cross-validation trials (averaged across folds per trial, see Methods). On average, 67 AMR traits were selected per model (range: 17–91), with a total of 339 unique traits identified across all models, Supplementary Data 6. Many traits (75%) recurred across multiple models, particularly among antibiotics from the same class Fig. S11. Among the ML-selected traits, 205 traits were classified as high-risk (28 Rank V and 177 Rank IV), Supplementary Data 6. The remaining 128 were of lower risk: 76 classified as medium-risk (Rank III) and 58 as low-risk (Rank I and II). Of the 205 high-risk traits, 98% were mobile (IS-ARGs, Plasmid-ARGs or IS-Plasmid-ARGs) in at least one isolate, with 69.8% associated with ISs (IS-ARGs or Plasmid-IS-ARGs). The same ML pipeline, when applied to the non-MDR subset, identified 18 traits correlated with resistance profiles, see Supplementary Results, Supplementary Data 7, Table S2 and Fig. S12.

ML-selected features were not disproportionately associated with the dominant serovars. Specifically, after normalising by the serovar frequency, the median trait enrichment in the ML selected features was 1.02, i.e., consistent with the baseline serovar distribution. In contrast, the non-selected traits had a median enrichment of 0.12, indicating a strong skew towards rare serovars. This indicates that ML-selected, high-risk traits are distributed across serotypes in proportion to their overall prevalence in the dataset, providing no evidence of dominant-serovar bias. The lower enrichment score observed for non-selected traits reflects the fact that many such traits are rare, isolate-specific findings restricted to less common serovars, which were appropriately excluded by the cross-validation stability criterion (≥80% of CV trials) rather than by any serovar-related selection bias.

We sought to test the predictive performance of the models beyond the cross-validation already employed within the pipeline. The full dataset was partitioned into a training set comprising all pre-2021 isolates (*n* = 2286) and a within-group validation set comprising all 2021–2022 isolates (*n* = 250). The original GLMM methodology was applied unchanged to the training set only, including cross-validation and final model fitting. The resulting fixed-effect coefficients were applied to the within-group validation set to calculate predictive performance metrics. Models with poor performance in the original analysis (AUC < 0.7; MXF and NAL) were excluded from summary reporting, though their results are included in Supplementary Data 8 for completeness. Excluding DOX, for which the small holdout sample size (*n* = 36) precluded stable estimation (see below), the mean AUC was 0.94 ± 0.04 (sensitivity 0.86 ± 0.14, specificity 0.85 ± 0.15), confirming that model performance remains high when applied to data not used during model fitting. External AUC values were generally higher than those obtained from cross-validation, reflecting the more stringent design of the original pipeline, which used stratified grouped k-fold cross-validation to assess performance generalised to genomic lineages not seen in the training data. STR and CHL showed poorer performance on some metrics, attributable to extreme class imbalance (≥95% resistant) within the validation set rather than to deficiencies in the underlying model. DOX showed reduced performance attributable to small sample size (*n* = 36) within the validation set. For KAN, the within-group holdout set (*n* = 39) contained no susceptible isolates (100% resistant), meaning AUC and precision could not be calculated and are reported as undefined rather than reduced; the corresponding accuracy, PR-AUC, precision, and sensitivity metrics of 1.0 should be interpreted with this caveat rather than as evidence of strong discriminatory performance

As a further stress-test, we applied the final fitted models to an independent dataset of human and food *Salmonella* isolates collected in Guangdong province between 2017 and 2024^21^, comprising diarrhoeal patients from sentinel hospitals and food sources including pre-packaged ready-to-eat foods, cooked foods, pastry products, baked goods, pre-prepared dishes, and vegetables. We note that although all patients had diarrhoeal *Salmonella* infections, the original study did not present evidence of a direct link to the food chain. While this external dataset differs substantially from ours in geography, source composition, and resistance prevalence, it provides a useful stress-test of the modelling approach under conditions of dataset shift. Despite these differences, the fitted models retained discriminatory performance in this altered epidemiological context. Of five comparable antibiotic models, three achieved AUCs comparable to or exceeding the full model AUC (relative AUC: AZM 1.05, CAZ 1.04, SXT 0.98), and the remaining two showed slightly reduced AUCs that nonetheless remained within the standard deviation of the original model performance (relative AUC: CIP 0.84, CTX 0.89). Sensitivity and specificity were similarly maintained or improved in three models and reduced in the other two (Supplementary Data 6 and 9). Some models showed reduced precision (AZM, CTX), driven by large shifts in the proportion of resistance between datasets (AZM: 26% to 5%; CTX: 43% to 16%), illustrating the effect of dataset shift and the importance of recalibration when applying the pipeline in altered epidemiological contexts.

### Health, governance, socioeconomic factors, and antibiotic usage are associated with high-risk AMR trait prevalence, highlighting emerging global threats in the food chain

We then investigated which of the 205 high-risk AMR traits are likely to spread within China’s food chain over the next 25 years and analysed the key indicators associated with these trends.

Specifically, we assessed how factors, such as mortality, healthcare access, socioeconomic status, governance, antibiotic consumption, and climatic conditions, are associated with the spread of AMR traits in MDR *S. enterica* across One Health domains of China’s food chain, quantified using 96 measurable indicators, see Supplementary Data 10. We note that these indicators were collected at national level; province-level GDP per capita data for the 15 study provinces over the study period are provided in Supplementary Data 11 to contextualise the geographic heterogeneity that national-level indicators smooth over.

Using validated forecasting pipelines from a global study^22^, we projected the prevalence of each trait through 2050 and benchmarked indicator forecasts for current health expenditure per capita and the proportion of population over 65 against independent sources^23,24^; our projections were consistent with published estimates, see Supplementary Information and Fig. S13 for details.

Of the 205 ML-selected traits, 165 were associated with at least one indicator with a Pearson correlation |*r*| > 0.5. Most of the 165 traits were strongly associated with socioeconomic (75%, e.g. poverty gap at $2.15/day; the percentage of people living below 50% of median income; and access to basic handwashing facilities), health-related (71%), antimicrobial consumption (51%, e.g. macrolide, lincosamide, and streptogramin usage; β-lactam usage; quinolone usage; and total antibacterial consumption), and climate indicators (53%), Supplementary Data 12.

**Fig. 2:**
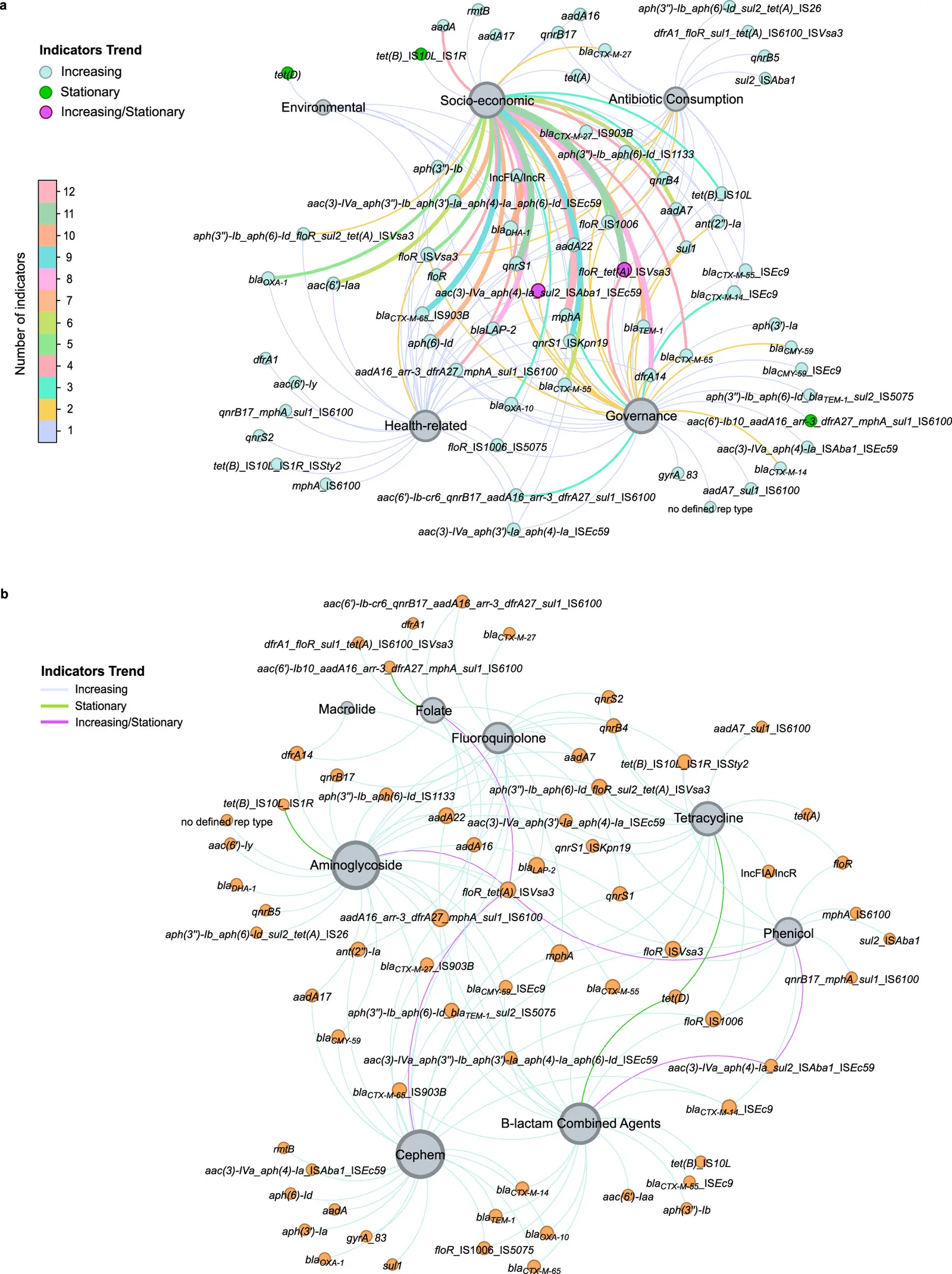
Undirected networks showing associations between high-risk AMR traits and increasing or stationary indicators and antibiotic class. **a** Network of AMR trait nodes color-coded by their indicator trend: increasing (blue), stationary (green), or both increasing and stationary (purple). Indicator type nodes, representing health-related, antibiotic consumption, socioeconomic, and governance categories, are depicted as silver circles, sized proportionally to the number of indicators within each group. Edges represent associations between AMR traits and indicator types, with edge colour and thickness reflecting the number of individual increasing or stationary indicators underlying the association. **b** Undirected network connecting AMR trait nodes to antibiotic class nodes, where each trait edge is color-coded based on its projected trend, increasing, decreasing, stationary, or a combination thereof, derived from its associations with different indicator groups. Antibiotic class nodes are shown as silver circles, sized proportionally to the number of connected features. Edge colours correspond to indicator trends: increasing (blue), stationary (green), or both increasing and stationary (purple). Source data are provided as a Source Data file.

Of the 205 high-risk traits, 101 were forecastable, and 84 were forecast to increase over time, linked to an average of six (range: 1–25) indicators with decreasing, increasing, or stationary trends, resulting in 170 trait-antibiotic model pairs Fig. 2 and Supplementary Data 13, across 84 unique AMR traits. Two-thirds (65.3%) of forecastable traits were associated with more than one indicator type, socioeconomic indicators (*n* = 52) and health-related indicators (*n* = 48) were linked to the most traits, Fig. 2a. Among the 170 trait-antibiotic pairs, 52 showed consistent increasing/stationary trends, 36 showed consistent decreasing trends, and 82 were mixed, Fig. 2b. AMR traits linked to decreasing indicators were considered less relevant to future AMR risk and were not considered in further analyses. Of the 84 high-risk ML-selected AMR traits, 70 were forecast to increase in association with increasing or stationary indicator trends. Among these, 71% were multidrug-resistant, 98% were found across more than one province, and 98% were found across two or more *Salmonella* serogroups, Fig. S14 and Supplementary Data 13.

Forecasts revealed distinct trajectories even within antibiotic classes. For example, while all classes were associated with socioeconomic (e.g., GDP, population density) and governance indicators (i.e., government effectiveness), folate and macrolide classes were not associated with health-related indicators, see Fig. 2.

**Fig. 3:**
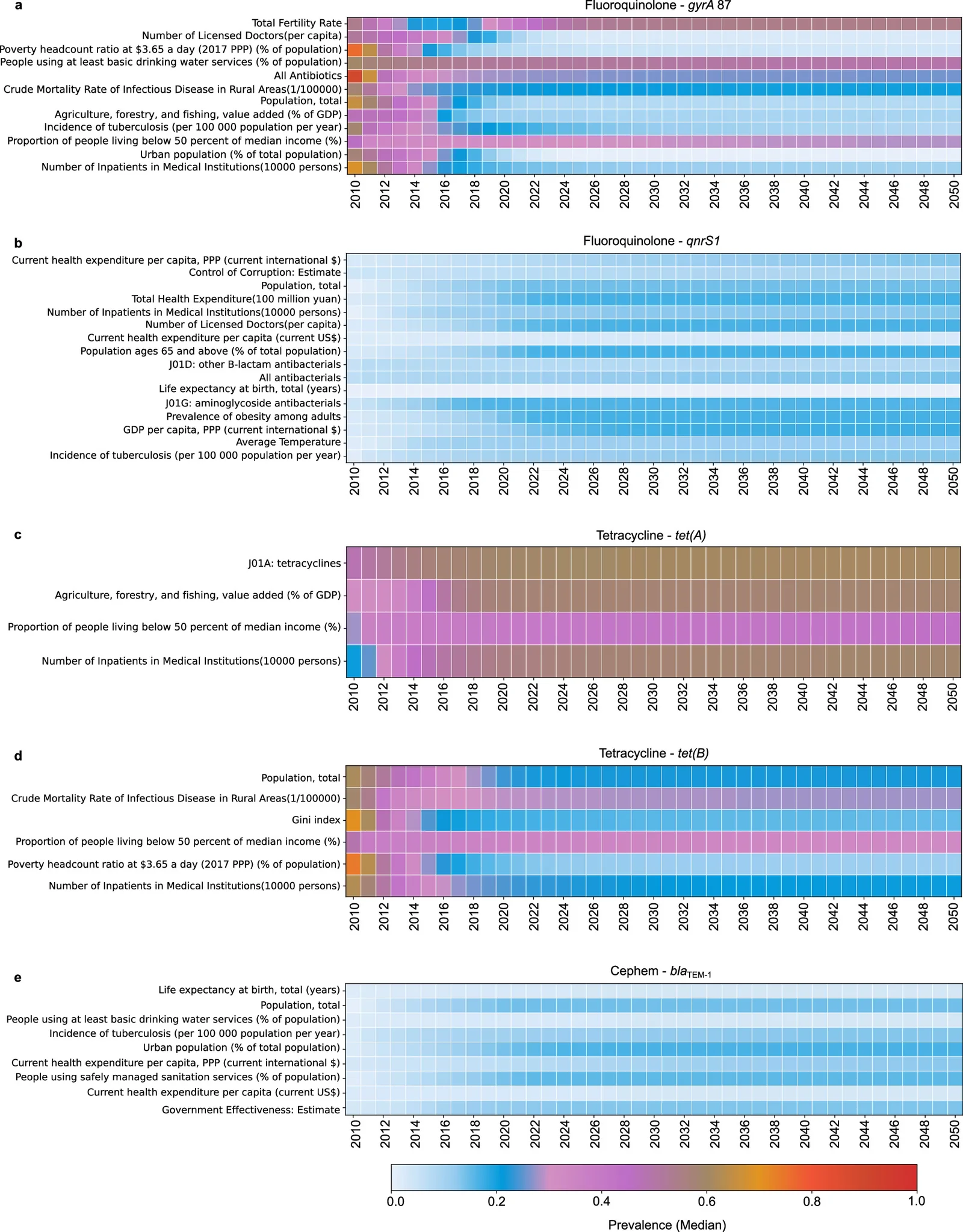
Forecast heatmaps for fluoroquinolone, tetracycline, and cephem antibiotic resistance models. The heatmaps illustrate the median predicted prevalence of key AMR traits in *S. enterica*, based on Monte Carlo simulations extending to 2050. Each panel shows the median forecast from 10,000 simulations, with colour intensity representing feature prevalence rates ranging from 0 to 1 (as shown in the scale bar). The forecasts are strongly correlated with increasing or stationary health-related indicator determinants. (**a**) *gyrA* 87 in fluoroquinolone-resistant *S. enterica*, (**b**) *qnrS1* in fluoroquinolone-resistant *S. enterica*, (**c**) *tet(A)* in tetracycline-resistant *S. enterica*, (**d**) *tet(B)* in tetracycline-resistant *S. enterica*, and (**e**) *bla*_TEM-1_ in cephem-resistant *S. enterica*. Source data are provided as a Source Data file.

Fluoroquinolone resistance was associated with both mutation-based resistance (*gyrA* 87) and acquired resistance genes. However, *gyrA* 87 prevalence was predicted to decrease in association with 13 indicators, while acquired resistance genes such as *qnrS1* were generally predicted to increase over the next 25 years. The increasing prevalence of quinolone resistance genes, *qnrB17*, *qnrA1*, *qnrS2*, and *aac(6’)-Ib-cr6_qnrB17_aadA16_arr-3_dfrA27_sul1_*IS*6100*, was associated with 21 indicators, of which seven were socioeconomic, including current health expenditure, poverty and aging population, Figs. 3a, b and S15–16.

Interestingly, genes within the same antibiotic class were predicted to follow different trajectories. For example, *tet(A)* and *tet(M)* were forecast to increase, whereas *tet(B)* was forecast to decrease in prevalence over time, Fig. 3c, d and S17–18. Similarly, for cephem resistance, *bla*_CTX-M-64_ and *bla*_TEM-1_ were projected to rise up to 20% in prevalence by 2050, however, closely related genes such as *bla*_CTX-M-65_, was only projected to rise to 5% by 2050 Figs. 3e and S19–S20. These divergent patterns were also observed across other antibiotic classes, including aminoglycoside, β-lactam combination agent, folate, macrolide, and phenicol resistance, Supplementary Results and Fig. S21–S30.

### Indicator-indicator correlation and robustness analyses

To assess multicollinearity among the 96 indicators used in the forecasting pipeline, pairwise Pearson correlations were calculated between all indicator pairs following time-series preprocessing (see Methods). Indicator-indicator associations were on average weak (mean |*r*| = 0.237; range: (−0.999 to 1), with the majority of indicator pairs showing low correlation (Fig. S31). Strong associations (|*r*| > 0.5) were limited and generally confined to indicators within the same category, with the two largest correlated clusters comprising antibiotic usage indicators and socioeconomic poverty gap indicators, respectively. Cross-category correlations were generally weak, supporting the independence of associations across indicator domains.

Principal component analysis identified two major components explaining 31.97% of total indicator variance, with 8 components needed to explain 80%. Principal component (PC) 1 reflected broad economic development (loading strongly on poverty indicators, healthcare expenditure, and urbanisation), while PC2 was dominated by antibiotic consumption patterns with a small number of poverty indicators, other components were mixed. The average top PC fraction (proportion of the total trait-associated absolute PCA loading score attributable to a single PCA component) was only 10.6%, which was not larger than would have been expected by chance (one-tailed permutation test, *p*-value = 0.999, see Methods). Similarly, the mean top-versus-second PC margin was also small (0.0126; one-tailed permutation test, *p*-value = 0.990). These high *p*-values indicate that the observed trait-PC specificity was no greater than expected under random trait-label assignment, consistent with AMR traits being associated with multiple independent components rather than concentrated on a single dominant axis of variation, supporting the gene-specific interpretation of the indicator associations.

A sensitivity analysis repeating the forecasting pipeline with one representative indicator per correlated cluster confirmed that the set of forecastable traits and the direction of their projected trajectories were consistent with the main analysis (95% of AMR traits retained), demonstrating robustness to indicator redundancy within correlated clusters.

Examination of indicator category breadth showed that 78.2% of forecastable AMR traits were associated with indicators from two or more distinct indicator groups and 81.2% were associated with two or more distinct indicator clusters, indicating that their projected trajectories are supported by convergent evidence across independent indicator domains.

## Discussion

Despite the rising global burden of AMR, responses often remain reactive rather than preventive, particularly in LMICs where surveillance and reporting systems are often fragmented. Effective prevention requires not only identifying the source and location of resistant pathogens and contaminants but also understanding the associated indicators and their potential future trends. This challenge is particularly pronounced for AMR in foodborne infections, due to the complex and multi-sectoral nature of the human, animal, environmental, and food interfaces. Important gaps remain in our knowledge of the similarities and differences in risk factors for AMR across humans, animals, environment, and food.

Several frameworks have been proposed to assess the risk posed by AMR genes, most notably the omics-based framework of Zhang et al.^25^, which ranks ARGs according to human enrichment, mobility, and association with ESKAPE pathogens, and the WHO AWARE classification^19^, which stratifies antibiotic classes by their importance for human medicine and their role in driving resistance. Our risk assessment framework explicitly incorporates both of these established approaches as components of the clinical relevance dimension of our scoring matrix: the Zhang et al.^25^ Rank I criteria contribute directly to the ARG impact score, and the WHO AWARE classification informs the antibiotic class usage score across all five tiers. However, our framework extends these prior approaches in three important respects. First, it operates at the level of AMR traits, specifically including ARG–MGE combinations rather than ARGs in isolation, capturing the genomic mobility context of each resistance element, which is critical for assessing transmission potential. Second, it explicitly quantifies inter-host and inter-serovar mobility as dimensions of likelihood of spread, using empirical distribution data across One Health source types and serovars within our dataset, neither of which is captured in existing frameworks. Third, it is calibrated to the specific antibiotic usage patterns and One Health food chain context of China, incorporating source-type distribution data from human, animal, food, and environmental isolates collected over 16 years. To our knowledge, no existing ARG risk framework simultaneously integrates MGE context, empirical cross-host mobility, and setting-specific antibiotic usage in this way.

By integrating AMR genomic surveillance of *S. enterica* across the One Health spectrum with national-level socioeconomic, climate, and health data, our framework identified clinically relevant AMR traits likely to spread through China’s food chain over the next 25 years and the key indicators associated with these trends.

A key finding is that not all AMR genes within the same antibiotic class pose equal long-term risk. For example, the third-generation cephalosporin resistance genes *bla*_CTX-M-64_ and *bla*_TEM-1_ are projected to increase by up to 20% in prevalence in China by 2050, associated with socioeconomic factors such as population growth, healthcare spending, and hospital infrastructure. In contrast, *bla*_CTX-M-65_, is forecast to remain below 5%, associated with differing socioeconomic and governance pressures. These findings align with previous works linking carbapenem and third-generation cephalosporin resistance to healthcare investment, hospital density, and urbanisation^1^, and with studies showing a rise of *bla*_CTX-M_ in Chinese foodborne *S. enterica* isolates^26,27^. Analogously, *tet(A)* and *tet(M)* were forecast to increase, while *tet(B)* was forecast to decrease in prevalence over time. This may be explained by differences in the genomic locations of these genes within the food chain. Our data showed that *tet(A)* and *tet(M)* were integrated into the chromosome more frequently than *tet(B)*, which was predominantly carried as a mobile element. Although both *tet(A)* and *tet(M)* are traditionally considered plasmid-carried resistance genes, our findings suggest a shift towards chromosomal integration, consistent with the latest studies of tetracycline resistance in the food chain in China, which have found *tet(A)* to be associated with clonal expansions of serovars^28^ and carried both on plasmids and chromosomally^29^. This flexibility in gene carriage could explain the expected dominance of *tet(A)* and *tet(M)* over *tet(B)* mediated resistance over the next 25 years. However, the gene- and indicator-specific resolution provided by our pipeline highlights the need to move beyond class-level risk assessments toward more granular gene-level surveillance and policy action. By capturing these granular patterns, our findings not only extend previous risk classifications^25^, but demonstrate how individual AMR genes may evolve under future, region-specific foodborne exposure scenarios.

Fluoroquinolone resistance in *S. enterica*, a WHO-designated priority threat, is projected to rise across all One Health domains in China by 2050. Notably, our findings reveal a mechanistic shift in the basis of this resistance: mutation-based resistance (*gyrA* 87) is projected to decline over the next 25 years, while acquired plasmid-mediated resistance genes including *qnrB17* and *qnrS1* are predicted to increase. This divergence may reflect both the known fitness costs associated with *gyrA* 87 substitutions^30–32^, which may reduce competitive fitness under relaxed selective pressure, and the progressive impact of China’s antibiotic stewardship policies, including the 2020 ban on quinolone premixes as veterinary feed additives. However, the simultaneous rise in acquired resistance genes suggests that stewardship measures targeting growth promoter use alone may be insufficient: plasmid-mediated quinolone resistance genes are transferable horizontally and can persist and spread even as chromosomal mutation-based mechanisms decline. Five genes, including *qnrB17* and *qnrS1*, often associated with multidrug resistance, are projected to increase in prevalence, associated with antibiotic usage, including fluoroquinolone and tetracycline use, urbanisation, and economic growth. Given that fluoroquinolones remain first-line treatments for invasive *S. enterica* infections, this shift from chromosomal to mobile acquired resistance represents a critical and underappreciated public health threat, as it enables horizontal spread across serovars, hosts, and settings in ways that mutation-based resistance cannot. These findings underscore the need for stewardship policies that specifically target plasmid-mediated quinolone resistance, including surveillance of PMQR genes in both clinical and food chain settings.

Gene-specific associations were also observed with GDP (e.g., *dfrA14*, *qnrS1*), population (e.g., *floR_tet(A)_*IS*Vsa3*), and climatic factors such as temperature and precipitation (e.g., *bla*_DHA-1_, *tet(D)*). These patterns extend earlier studies done in China^33^ linking these factors to average ARG counts (the average number of ARGs per isolate in decade-specific data sets). Our analysis extends this by providing gene-specific prevalence forecasts linked to GDP (e.g., *dfrA14*, *qnrS1*) and population density (e.g., *floR_tet(A)_*IS*Vsa3*).

Notably, in our study, food workers emerged as potential AMR hotspots, though no direct evidence of transmission was established. They not only carried AMR traits conferring resistance to last-resort antibiotics such as colistin (e.g., *mcr-9.1, mcr-1.1_*IS*1A*), carbapenems (*bla*_NDM-1_*, bla*_NDM-5_), and β-lactams (*bla*_CTX-M-55_*, bla*_CTX-M-64_), but also carried significantly higher numbers of high-risk AMR traits than food sources. The analysis of serovar and lineage (PopPUNK clusters) composition showed similarity between food- and non-food workers' isolates, suggesting shared exposures or potential human-to-human transmission. These findings align with our previous works showing genetic similarities between *Escherichia coli* isolates from food animals, their environments, and food workers and their households in China^13^, as well as our recent work demonstrating higher MDR *S. enterica* carriage, greater serovar diversity, and higher resistance rates in food workers to non-food workers^17^. By expanding the analysis across One Health domains, we identified the ARGs driving resistance spreading across sources.

These findings substantially extend our recent landmark study by Lu et al., which demonstrated, across 260,315 asymptomatic workers in Yulin, that food workers carry *Salmonella* at higher rates and with greater serovar diversity than non-food workers, with plasmid-mediated ARGs as the dominant resistance vehicle. While that study provided foundational evidence for food workers as *Salmonella* reservoirs within a single-city, two-group framework, our analysis advances this in four important ways. First, by spanning 15 provinces and the full One Health food chain, including retail meat, livestock, and farm environments alongside human sources, we demonstrate that food workers not only carry more resistance traits than non-food workers but carry significantly more high-risk traits than food sources themselves (*p*-value = 4.289 × 10⁻²¹), and uniquely harbour last-resort resistance genes absent from food sources. Second, by applying a population-structure-corrected machine learning framework with Bayesian mixed effects and PopPUNK stratification, we disentangle true AMR trait associations from lineage confounding, a methodological limitation of prior correlation-based approaches. Third, by incorporating 96 national-level socioeconomic, climate, and health indicators with FDR correction, we identify the structural factors, including poverty, urbanisation, healthcare spending, and antibiotic consumption, associated with resistance trajectories at a scale and resolution not previously reported. Fourth, we project these trajectories to 2050, providing gene-specific AMR forecasts across China’s One Health food chain. Together, these advances transform the descriptive picture of food worker AMR carriage into a predictive, mechanistically grounded framework for targeted intervention.

The AMR traits identified and forecast in this study are predominantly carried on mobile genetic elements capable of horizontal gene transfer across bacterial species. Many of the high-risk traits projected to increase, including *bla*_CTX-M_ variants, plasmid-mediated quinolone resistance genes, and aminoglycoside resistance determinants, have been documented across multiple Gram-negative foodborne pathogens sharing the same ecological niches as *S. enterica* in China’s food chain, including *E. coli*^34–36^, *Campylobacter* spp.^37,38^, *Klebsiella pneumoniae*^39,40^. The socioeconomic, healthcare, and antibiotic consumption pressures identified as correlates of AMR trait prevalence in our study act on the food chain resistome broadly, suggesting that the directional trends forecast here may have relevance beyond *S. enterica*. This is consistent with our previous work demonstrating shared ARGs and mobile genetic elements across *S. enterica* and *E. coli* co-inhabiting the same farm environments and food worker populations in China^15,16^. Nevertheless, direct quantitative extrapolation to other species requires caution, as prevalence projections are calibrated to *S. enterica*-specific genomic and ecological contexts. Applying equivalent surveillance and forecasting frameworks to other priority foodborne pathogens, particularly *E. coli, Campylobacter spp*., *and K. pneumoniae*, represents an important direction for future work and would provide a more complete picture of the trajectory of the food chain resistome under the socioeconomic and environmental pressures projected to 2050.

A potential concern in any study spanning multiple *Salmonella* serovars is whether reported AMR patterns reflect broad species-level trends or are driven primarily by dominant serotype-specific effects. We addressed this at multiple levels of the analysis. First, population structure of which serovar is a major component, given the high correlation between serovar and sequence type in our dataset (Cramer’s *V* = 0.85, *p*-value < 2.2 × 10⁻^16^) was explicitly modelled as a random effect in all GLMM models via PopPUNK cluster membership, which provides higher-resolution lineage classification than serovar designation alone. The odds ratios reported for each genomic feature therefore represent within-lineage effect sizes, conditional on population structure, and cannot be attributed to serovar composition differences between source types. Second, the risk assessment framework explicitly deprioritises traits confined to a single serovar through the inter-serovar mobility score, such that all traits classified as high-risk (Rank IV or V) are by definition present across multiple serovars. Consistent with this, 98% of the 84 traits forecast to increase were found across two or more serogroups. Third, cross-validation stratified by PopPUNK cluster ensures that retained features generalise across lineage boundaries: features selected under this regime are precisely those not confined to dominant lineages. To further address this point, we performed a *post hoc* analysis quantifying the serovar distribution of all ML-selected traits, confirming that selected traits are not disproportionately associated with the dominant serovars relative to their prevalence in the full dataset.

The gene- and indicator-level resolution of our framework provides added value over class-based AMR risk assessments across clinical, public health, and policy dimensions. Clinically, genes within the same antibiotic class can differ fundamentally in their resistance mechanisms, mobility, and fitness costs, with direct consequences for treatment and containment. Our findings illustrate this clearly: within fluoroquinolone resistance, mutation-based resistance (*gyrA* 87) and plasmid-mediated resistance genes (q*nrB17*, *qnrS1*) are projected to follow opposite trajectories. Because plasmid-mediated quinolone resistance (PMQR) genes are transferable horizontally across serovars and hosts at frequencies much higher than chromosomal mutations, their rise represents a qualitatively different and more serious public health threat than an equivalent rise in mutation-based resistance, a distinction entirely obscured by class-level assessment. Similarly, within third-generation cephalosporin resistance, *bla*_CTX-M-64_ and *bla*_TEM-1_ are projected to increase by up to 20%, while *bla*_CTX-M-65_ is projected to remain below 5%, and within tetracycline resistance, *tet(A)* and *tet(M)* are forecast to increase while *tet(B)* decreases, divergences that would partially cancel at the class level, potentially creating false reassurance or masking genuinely rising threats.

For public health surveillance, gene-level forecasting enables evidence-based prioritisation of specific resistance determinants for monitoring, an important practical consideration in resource-limited settings where surveillance capacity cannot target all resistance equally. Our risk stratification framework, integrating clinical relevance, cross-host mobility, and serovar spread into a single score per genomic trait, provides a basis for tiered surveillance responses proportional to actual transmission risk, aligned with the call for strengthened genomic surveillance within the recently updated WHO Global Action Plan on AMR (2026–2036)^41^.

For policy, the indicator-level associations reveal that different genes within the same antibiotic class are driven by different structural factors, and therefore require different interventions. The rise of PMQR genes is linked to urbanisation, healthcare spending, and demographic change alongside antibiotic consumption, suggesting that veterinary stewardship measures alone are insufficient and that complementary interventions targeting horizontal transmission in healthcare and urban food systems are needed. More broadly, the association of specific resistance genes with governance indicators alongside socioeconomic ones suggests that effective AMR policy must engage with health system governance rather than antibiotic use alone, consistent with recent evidence that national action plans have thus far inadequately addressed the structural socioeconomic drivers of AMR. Together, these layers of resolution transform gene-level surveillance data into a mechanistically grounded, policy-actionable framework for targeted AMR intervention.

This work has several limitations that need to be acknowledged. A key limitation concerns the unbalanced sampling design across source categories, geographic regions, and collection periods. Food worker and non-food worker isolates were collected exclusively from Guangxi Province as part of a structured occupational health surveillance programme, whereas retail meat, livestock, farm environment, and symptomatic patient isolates were derived from different provinces and distinct surveillance windows spanning 2006–2022. This structural heterogeneity means that comparisons of AMR trait prevalence across source types are potentially confounded by geographic and temporal co-variation that cannot be fully resolved within the modelling framework.

Province, year, source type, and lineage (PopPUNK cluster) were included as random effects in all Bayesian mixed effects models, and interclass correlation coefficients confirm that province (ICC = 0.149) and year (ICC = 0.164) were the largest sources of model variance, indicating that the analytical framework recognised and partially absorbed geographic and temporal signal. However, random effects only partially absorb variation due to grouping factors, and ICC values reflect variance attribution rather than confound elimination. Because food worker isolates originate exclusively from Guangxi, the data contain no cross-province, within-source-type contrasts to fully disentangle a source-type effect from a province-specific effect, and residual confounding cannot be excluded.

This limitation has specific implications for three aspects of the analysis. First, source-specific AMR trait prevalence estimates, particularly the elevated trait richness and high-risk trait burden observed in food workers, may partly reflect Guangxi’s regional AMR epidemiology, the intensive longitudinal nature of occupational screening, or secular trends during the years of collection, rather than being solely attributable to occupational exposure. Second, while the identification of food workers as potential AMR hotspots is supported by a formal statistical test (proportion Z-test, *p*-value = 4.289×10⁻²¹) and is further supported by the bootstrapped human group comparisons reported in the Results, this test was conducted on raw proportions and does not itself adjust for province or year; the geographic and temporal confounding discussed above therefore applies directly to this comparison and should be considered when interpreting the magnitude, though not necessarily the direction, of this finding. Furthermore, this finding should be interpreted as an epidemiological association rather than a causally attributed source effect. Third, forecasting models are calibrated against national-level socioeconomic and health indicators and may not capture province-specific resistance trajectories, which is of particular relevance for traits disproportionately represented in the Guangxi food worker cohort. Future studies would benefit substantially from prospective, multi-province sampling of all source types within overlapping geographic and temporal windows, which would provide the within-province cross-source contrasts necessary for more rigorous causal inference and regionally stratified forecasting.

The study design also allowed for repeated sampling of food and non-food workers as part of routine occupational health screening. Although this is a potential source of bias in the models, its effects were minimised by ensuring all individuals testing positive were treated until they received a negative test result, reducing the likelihood of strains being sequenced multiple times, and increasing the likelihood of independent infections. However, we acknowledge that the experimental approach does not guarantee independence between isolates from the same individual. Additionally, we acknowledge that reconstructing plasmids from short-read data is inherently challenging; the tools used represent current best practice when long-read data are unavailable, and our pipeline incorporates multiple independent tools to minimise false-positive and false-negative results. Nevertheless, some inaccuracies in plasmid reconstructions are expected.

A further limitation concerns the correlational nature of all indicator-trait associations reported in the forecasting analysis. Despite the application of stationarity testing, cointegration testing, first-order differencing, and permutation-based FDR correction to mitigate spurious associations as a result of shared temporal trends, the pairwise framework cannot establish causality or directionality between individual indicators and AMR trait prevalence. Some indicators reflecting broad economic development trajectories, including healthcare expenditure, urbanisation, and population growth, may act in part as proxies for a shared underlying driver that cannot be fully decomposed through pairwise analyses given the constraints of national-level time series data and the limited number of annual observations available (maximum 17 years of overlap). This is an inherent limitation of ecological surveillance studies at the national level. The indicator-trait associations reported here should therefore be interpreted as identifying candidate structural factors associated with AMR trait prevalence warranting further investigation through prospective, regionally stratified study designs, rather than as evidence of direct causal relationships. Future studies incorporating subnational data, longitudinal cohort designs, or natural experiment frameworks, such as policy changes affecting antibiotic use, would enable stronger causal inference about the structural determinants of AMR spread across China’s food chain.

A further limitation of our forecasting analysis relates to the use of national-level aggregate values for socioeconomic, environmental, and health indicators to represent all 15 provinces included in the study. China exhibits substantial geographic heterogeneity across all indicator categories used: per capita GDP varies more than fourfold across provinces, and Guangxi, from which the majority of food worker isolates in our dataset were collected, had a per capita GDP of approximately 52,399 yuan in 2022, approximately 40% below the national average of 87,385 yuan reported for that year^42^, reflecting its status as one of the economically less developed provinces in our dataset relative to coastal provinces such as Shandong and Guangdong. Province-level variation in healthcare workforce density and health expenditure efficiency is similarly substantial across China^43^, and within-province variation in antibiotic consumption has been documented^44^. We note that province is explicitly included as a random effect in all GLMM models, with province ICC (0.149) among the largest of the four random effects, indicating that province-level variance in AMR trait prevalence is accounted for in the genotype-phenotype associations. Nevertheless, province-level time series for many of the indicators used in our forecasting, particularly antibiotic consumption, governance metrics, and several health indicators, are not currently available in the public databases from which our data were drawn. A supplementary analysis extracting province-level GDP per capita for the 15 study provinces from the NBS China Statistical Yearbook^42^ is provided in Supplementary Data 11, quantifying the geographic heterogeneity that national-level indicators smooth over. Future studies incorporating province-level indicator data, as these become available through expanding national surveillance databases, would enable regionally targeted AMR forecasts of direct relevance to provincial public health policy.

Our forecasts are constrained by national-level data and inherent sampling biases that cannot be completely compensated for within the modelling framework. The forecasting window of 25 years is long relative to the time series data used to calibrate the models, and future recalibration with additional years of data will improve the certainty of the longer-range forecasts. Future studies would benefit from regionally focused sampling and forecasting designs, enabling regionally targeted insights and policy interventions.

We further note that the associations identified between AMR trait prevalence and socioeconomic, health, and climate indicators, while statistically robust and FDR-corrected, are correlational in nature: the forecasting pipeline itself quantifies association, not causal mechanism, between these indicators and AMR trait prevalence. Where we offer biologically-grounded interpretation of specific findings elsewhere in this Discussion, for example regarding the divergence between mutation-based and plasmid-mediated fluoroquinolone resistance, or the genomic-location-dependent trajectories of *tet(A)*, *tet(M)*, and *tet(B)*, these interpretations draw on independent evidence (e.g., known fitness costs of resistance mutations, documented antibiotic stewardship policy timelines, and genomic location data generated in this study) and are presented as plausible explanatory context for the patterns observed, rather than as conclusions established by the forecasting pipeline’s correlational methodology.

While our cross-validation framework, stratified by PopPUNK cluster to evaluate performance across held-out genomic lineages, already provides a rigorous internal assessment of model generalisability, we additionally validated our fitted models against two independent test sets not used in model fitting: a temporal holdout comprising isolates collected in 2021–2022, and an out-of-group dataset of human and food *Salmonella* isolates from Guangdong province^21^. Both analyses demonstrated that model performance was largely maintained, with some expected attenuation in the out-of-group analysis attributable to shifts in baseline resistance prevalence between datasets. A systematic search of the literature and public sequence repositories confirmed that no directly comparable Chinese food worker or broader food-chain cohort with matched genomic and phenotypic data currently exists in the public domain, underscoring that our study represents the first large-scale genomic dataset of this kind. We note that the same analytical pipeline has been independently applied and validated at global scale across multiple pathogens and countries in a companion study^22^, supporting its broader methodological generalisability. Overall, these analyses demonstrate that our fitted models are robust to new data from within the study population, and retain meaningful discriminatory performance when applied to new data from outside it, despite substantial differences in geography, source composition, and resistance prevalence. This supports the validity of the underlying genotype–phenotype associations beyond the specific dataset used for fitting. We would, however, caution against direct clinical or surveillance deployment of the fixed, fitted coefficients in a new population without local recalibration, particularly where baseline resistance prevalence differs substantially, as our precision results illustrate. Our aim is therefore to provide a reusable analytical pipeline that can be re-fitted in different epidemiological contexts as local data accumulate, rather than a single static model intended for direct cross-population deployment. As whole-genome sequencing becomes increasingly integrated into China’s national AMR surveillance infrastructure, prospective validation using independent provincial or temporal cohorts, particularly from food worker or other occupational health screening programmes, remains an important priority for future work.

The insights in this study underscore the importance of targeting high-risk occupational groups in AMR prevention strategies and of integrating genomic, climate, and human health surveillance. In LMICs, food workers often connect farms, markets, households, and communities with limited protections, making them priority targets for surveillance and intervention. Although constrained by national-level data, our forecasts provide a blueprint for integrating genomic and contextual surveillance into policy. As sequencing becomes more accessible, region-specific forecasts will become increasingly vital.

Overall, we demonstrate how combining genomic, climate, and socioeconomic data can shift AMR control from reactive to predictive, enabling data-driven targeted, preventive strategies across One Health food systems.

## Methods

### Study design and bacterial isolates

We analysed separately 2536 MDR^45^ and 306 non-MDR *S. enterica* isolates collected across 15 provinces of China between 2006 and 2022, from One Health settings. Accessions for the DNA sequence data of all isolates are supplied in Supplementary Data 1. The MDR dataset comprised 2536 *S. enterica* isolates from public (*n* = 2257) and new (*n* = 279) sources; of these 2036 were isolated from humans^17,18,46^ (1697 food workers, 202 non-food workers, and 137 symptomatic patients), 366 from retail meat^18,46,47^ (319 pork and 47 chicken), 134 livestock and their environments^15^ (109 poultry and 25 farm environmental samples). The non-MDR dataset comprised 306 *S. enterica isolates* from public (*n* = 168) and new (*n* = 138) sources, of these, 225 were isolated from humans^17,18,46^ (155 food workers, 35 non-food workers, and 35 symptomatic patients), 72 from retail meat^18,46,47^ (31 pork and 41 chicken), and nine from livestock and their environments^15^ (six poultry and three farm environmental samples). Food worker and non-food worker isolates were collected from individuals during occupational health examinations, in one province (Guangxi) between 2013 and 2022^17^. Food worker and non-food worker classification followed the definitions established under Article 45 China’s Food Safety Law (2015), which distinguishes workers based on whether they enter in contact with food destined to direct consumption^48^. Classification was based on the recorded presence or absence of a food handler health certificate. Both groups were drawn from the same occupational health screening programme and therefore underwent equivalent health surveillance, differing specifically in their occupational exposure to food. Amongst these individuals, those presenting with diarrhoea and those who had taken antibiotics were excluded. Individuals may have given multiple samples over the time period covered; but as all data were anonymised, tracking of individuals was not possible. However, individuals testing positive for *Salmonella spp*. were treated with antibiotics until they tested negative; the associated *Salmonella* strains isolated at each timepoint were therefore unique. All other source types were derived from public data sources^15,18,46,47^. In brief, isolates from retail pork meat were collected from two surveillance studies across 14 provinces of China over a 13-year period (2006–2019)^18,47^, retail chicken meat isolates were collected from a surveillance study in one province (Shandong) in 2020^46^, symptomatic patients were drawn from two surveillance studies^46,47^ spanning two provinces of China over a 10-year period (2007–2016); and isolates from livestock and their environments were collected as part of an observational study of ten intensive poultry farms across three provinces, taken over a 3-year period (2019–2021)^15^.

The study was approved by the Ethics Committee of the National Institute for Communicable Disease Control and Prevention, Chinese Center for Disease Control and Prevention (approval number: ICDC-2022001), which waived informed consent and allowed secondary analysis of strains and data.

### Antimicrobial susceptibility testing

A total of 279 newly sequenced MDR and 138 newly sequenced non-MDR *S. enterica* isolates were tested for antimicrobial susceptibility using Phoenix NMIC413/NMIC801 panels (Becton, Dickinson Co., Franklin Lakes, NJ, USA). Pre-2022 NMIC413 panel includes 29 antimicrobial agents [amoxicillin-clavulanate (AMC), amikacin (AMK), ampicillin (AMP), azithromycin (AZI), aztreonam (AZM), ceftazidime (CAZ), cefazolin (CFZ), chloramphenicol (CHL), ciprofloxacin (CIP), colistin (COL), ceftriaxone (CRO), cefotaxime (CTX), cefuroxime (CXM), ceftazidime-avibactam (CZA), doxycycline (DOX), ertapenem (ETP), cefepime (FEP), fosfomycin w/G6P (FOS), cefoxitin (FOX), gentamicin (GEN), imipenem (IPM), kanamycin (KAN), levofloxacin (LVX), meropenem (MEN), minocycline (MIN), moxifloxacin (MXF), nalidixic acid (NAL), nitrofurantoin (NIT), norfloxacin (NOR), ampicillin-sulbactam (SAM), streptomycin (STR), sulfisoxazole (SUL), trimethoprim-sulfamethoxazole (SXT), tetracycline (TET), tobramycin (TOB), piperacillin-tazobactam (TZP), ceftiofur (XNL)]. NMIC801 includes 17 antimicrobial agents [amikacin (AMK), streptomycin (STR), tetracycline (TET), tigecycline (TGC), ampicillin (AMP), ampicillin-sulbactam (SAM), ertapenem (EIP), meropenem (MEM), ciprofloxacin (CIP), nalidixic acid (NAL), azithromycin (AZM), cefotaxime (CTX), ceftazidime (CAZ), ceftazidime-avibactam (CZA), chloramphenicol (CHL), colistin (COL), and trimethoprim-sulfamethoxazole (SXT)]. Minimum inhibitory concentrations (the lowest concentration of antibiotic that prevents visible growth) were interpreted to the Clinical and Laboratory Standards Institute criteria M100, 30th Edition breakpoints to define resistance. MIC values were interpreted as susceptible where the MIC value was ≤ the susceptible threshold, values ≥ the resistance threshold were interpreted as resistant, and values between those were interpreted as intermediate. *Escherichia coli* ATCC 25922 was used as a quality control strain during the antimicrobial susceptibility testing; see Supplementary Data 1. For publicly available isolates, AST data were taken directly from the original published sources. We reviewed the methods of each source publication to confirm the CLSI M100 edition used for breakpoint interpretation (27th, 28th, 30th, or 31st edition, depending on publication date) and cross-checked the relevant breakpoints for each antibiotic tested with our models across these editions (Table S2). Breakpoints for all antibiotics included in our analysis were unchanged across these editions for *Salmonella* spp., confirming interpretive consistency between the publicly available and newly generated AST data. For streptomycin and moxifloxacin no breakpoints are given in any of the CLSI M100 editions, so we relied on the RSI interpretations given in the original source. For both newly sequenced and publicly available isolates, resistance to ≥ 3 antibiotic classes was defined as MDR and isolates resistant to <3 as non-MDR.

### DNA extraction

Genomic DNA was extracted from 279 MDR and 138 non-MDR *Salmonella* isolates using the Wizard Genomic DNA Purification kit (Promega, WI, USA). DNA concentrations were quantified with the Qubit dsDNA HS Assay kit (Thermo Fisher Scientific, MA, USA). Libraries were constructed using the MGIEasy FS DNA Library Prep Set (MGI, China) and were paired-end sequenced on the MGISEQ-200RS and MGISEQ-2000RS platforms.

### Genome assembly and annotation

For newly sequenced data: Raw reads were processed using fastpv1.0.0 (parameters -q 30 -l 50), which filtered low-quality bases (*Q*  <  30) and retained only reads longer than 50 bp in length. Genomes were assembled using SPAdes v3.15.5 with the parameters ‘--careful --cov-cutoff auto’.

Assembly quality was assessed using QUAST v5.0.2 (with default parameters), and genome completeness and contamination were assessed using CheckMv1.2.2 (with default parameters) with the lineage_wf pipeline. Contigs shorter than 500 nucleotides were excluded from the final assemblies.

For newly sequenced genomes, assemblies were retained if they met the following quality thresholds: N50 ≥ 15 kb, genome completeness ≥ 80%, and contamination ≤ 5%; assemblies failing these criteria were excluded from further analysis. For publicly available assemblies: Sequences associated with BioProject PRJNA1082900 were obtained as pre-assembled genome sequences (assembled using SPAdes v3.15.5) from the National Genomics Data Center. These were also assessed against the same completeness and contamination thresholds described above prior to inclusion; assemblies failing these criteria were excluded.

For all other sequenced raw reads were downloaded from NCBI, and the same pipeline used for the newly sequenced data was applied. The same completeness and contamination thresholds were applied to all publicly available sequences; assemblies failing these criteria were excluded from further analysis.

Both publicly available and newly sequenced data were then aggregated, and genomes were annotated with Bakta v1.4.0, with “--genus Salmonella --force --compliant --skip-crispr”.

Sequence types were identified using MLSTv2.23.0 (with --scheme senterica_achtman_2), and serotypes and serogroups were identified in silico using SISTR v1.0.2 with default settings.

### Screening of annotated genes against ARG and MGE databases

Genome assemblies were screened against the CARD database (accessed 01-08-2024) using Abricate v1.0.1 at a stringent threshold of ≥ 95% coverage and ≥ 95% identity (parameters –db card –mincov 95 –minid 95) to identify known AMR-associated genes across the isolate cohort; these thresholds were chosen to minimise false positives and variant uncertainty^49^. AMR-associated genes were then manually curated, and ten genes (*BRP(MBL)*, *golS*, *mdsA*, *mdsB*, *mdsC*, *mdtK*, *mrx*, *qacEdelta1*, *sdiA* and *qacL*) that do not confer AMR in *S. enterica* were removed. Genome assemblies were also screened against the ISfinder database (accessed 01-08-2024) using Abricate v1.0.1 at the same thresholds (≥95% coverage and ≥95% identity) to identify insertion sequences across the isolate cohort.

### Mutation analysis

As mutation-based resistance is a major mechanism of fluoroquinolone resistance in *S. enterica*, sequences were screened for known *S. enterica* fluoroquinolone resistance mutations (*gyrA* codons 83 and 87; *gyrB* codons 464, 426, 466, and 431; *parC* codons 57 and 80). To screen, the gene alignment outputs from the pan-genome analysis for *gyrA*, *gyrB*, and *parC* were translated to protein sequences using Emboss Transeq v6.6.0.0 with default parameters. The animo acid at the known mutation position was extracted, and the susceptible amino acid was encoded as 0, with other amino acids encoded as 1. Only *gyrA* codons 83 and 87 were identified as having mutations in the dataset and were included as additional AMR traits.

### Plasmid reconstruction and mobile ARG cassettes identification

Contigs were screened using plasmidVerify v2.1.6^50^ (default parameters) and Plasmer v23.04.20 (default parameters) to identify putative plasmids, which were subsequently typed using MOBtyper v3.1.7 and PlasmidFinder v2.1.6 (default parameters). Putative plasmids were then reconstructed using two approaches: MOB-suite plasmid reconstruction and plasmid mapping, adapted from Cazares et al.^51^ For MOB-suite reconstruction, the MOB-recon algorithm in the MOB-suite package v3.1.7^52^ was used with default settings. To minimise false positives, putative plasmids shorter than 1.5 kb or lacking both a relaxase and replicon sequence were excluded^53^. For the mapping, putative plasmids were mapped against the PLSDB database using minimap2v2.30, retaining only those putative plasmids mapping with ≥ 70% coverage. The combined set of MOB-suite and mapped plasmids was considered confirmed plasmids in our analysis, and all remaining sequences were classified as chromosomally located. Confirmed plasmids were annotated with Bakta v1.4.0, with settings “--genus *Salmonella* --force --compliant --ski*p-*crispr”.

To identify mobile ARG content across source types, the distance between each identified ARG and MGEs was calculated from their positions^14^ (see above in “Screening of annotated genes against ARG and MGE databases and in silico subtyping”). ARG carrying contigs on which the distance between ARG and MGE exceeded 5 kb were discarded^14,54^, with the remaining contigs classified as mobile ARG cassettes. Where multiple ARGs and insertion sequences were located within 5 kb of each other, they were clustered as single mobile ARG cassettes, see Supplementary Data 2.

As a *post hoc* analysis we identified AMR traits, based on their location, specifically, ARGs on the bacterial chromosome (herein termed ARGs-Chr); ARGs on MGEs integrated into the chromosome near insertion sequences (herein termed ISs-ARGs); ARGs present between insertion sequences on plasmids (herein termed ISs-Plasmid-ARGs); ARGs on plasmids but not within an insertion sequence (herein termed Plasmid-ARGs); or classified as mixed if present in more than one location across isolates (e.g. the gene *fosA7* is present on the chromosome (ARGs-Chr) in some isolates and on a plasmid (Plasmid-ARGs) in others, so is categorised as mixed), across the food chain.

### Pangenome construction and phylogeny

All annotated genomes were used as input for pangenome analysis using Panaroo v1.5.0 with settings ‘-clean-mode strict --remove-invalid-genes -a core --core_threshold 0.99’ to obtain the core genome of genes present in ≥ 99% of the isolates. SNP variants were called from the core gene alignment using SNP*-*sites v2.5.1 (default settings). These core SNPs were used as input into IQ-TREE v2.1.4-beta (parameters –bb 10000) to generate a maximum likelihood phylogeny. In total 219,315 SNP sites were used, with the GTR + F + R7 substitution model selected based on model testing. The ultrafast bootstrap algorithm was used with 10000 replicates to assess branch support. The phylogenetic tree was visualised through iTOLv5^55^; branches with less than 95% bootstrap support were deleted.

### Risk analysis

We developed a risk assessment framework to estimate the transmission potential of AMR traits. The framework uses a standard 5×5 risk matrix combining two dimensions: the likelihood of spread and clinical relevance, resulting in five risk categories (Fig. 1). Final risk scores ranged from 1 to 25 and were classified as: Rank I (1–3), Rank II (4-9), Rank III (10-12), Rank IV (13–20), and Rank V (21–25) (Fig. 1 and Supplementary Data 4).

Likelihood of spread (transmission potential) scores reflected a trait’s capacity to cross host and serovar boundaries, with the highest scores assigned to traits found in humans, livestock, and food sources, and present in multiple serovars.

Likelihood of spread was defined as a combination of inter-host mobility (*c*) and inter-serovar mobility (*d*):

1 $${{{\rm{Likelihood}}}} \, {{{\rm{of }}}} \dot {{{\rm{spread}}}}=\min \left(5,{c}+d\right)$$

calculated based on the distribution of each AMR trait across source types and serovars in our dataset. Specifically, AMR traits were assigned an inter-host mobility score (*c*) of 1–5 based on the following criteria:

1. Present only in livestock sources (i.e., chicken farms or farm environments)
2. Present only in food sources (i.e., retail chicken or pork meat) or in food and livestock sources)
3. Present only in human sources (i.e., food workers, non-food workers, or symptomatic patients)
4. Present in human sources and in either food or livestock sources
5. Present in human sources, food sources, and livestock sources

In addition, AMR traits were assigned a score of 0 or 1 for inter-serovar mobility

2 $$d=\left\{\begin{array}{c}0\\ 1\end{array}\,\begin{array}{c} < 2 \, {\mbox{serovars}}\\ \ge 2 \, {\mbox{serovars}}\end{array}\right.$$

Clinical relevance scores reflected whether a trait conferred resistance to antibiotic classes used in humans and/or animals, their WHO AWARE status, and whether they met Rank I criteria defined by Zhang et al.^25^, which consider human enrichment, mobility, association with ESKAPE pathogens, and their capacity to confer multidrug resistance, with the highest scores assigned to MDR traits affecting critically important antibiotics for human and animal health.

Specifically, within each AMR trait, ARGs were assigned a score (*a*) between 1 and 4, based on the importance of the antibiotic class (Table S2) to which they conferred resistance and WHO AWARE status:

1. Antibiotic class not widely used in humans in China or on the AWARE Access list
2. Antibiotic class used only in humans in China and on the AWARE Watch or Reserve list
3. Antibiotic class used in both humans and animals in China and on the AWARE Watch or Reserve list
4. Rank 1 clinically relevant ARG according to Zhang et al.^25^

AMR traits were then assigned a multidrug resistance (MDR^45^) score (*m*) of 0 or 1 based on the number of antibiotic classes to which they conferred resistance:

3 $${{{\rm{m}}}}=\left\{\begin{array}{c}0\\ 1\end{array}\,\begin{array}{c} < 3\,{\mbox{classes}}\\ \ge 3\,{\mbox{classes}}\end{array}\right.$$

The final clinical relevance score was the sum of the MDR^45^ score (*m*, defined above) and the maximum ARG impact score (*a*, defined above):

4 $${\mbox{Clinical relevance}}=a+m$$

Risk categories (Rank I-V) were assigned based on the risk matrix in Fig. 1.

### Analysis of proportions of high-risk traits by source type

For each human source type-food pair, bootstrap resampling with 10,000 iterations was used to down-sample the larger group to obtain equal sample sizes. At each iteration, the difference in proportion of AMR traits observed in the now equally sized groups was calculated to provide an empirical distribution of differences in the proportion of AMR traits carried between the human source group and pooled food group. Mean difference and 95% confidence intervals were estimated. An empirical *p*-value was calculated as the proportion of bootstrap replicates showing in which the difference in proportions was less than or equal to 0 (given a positive observed effect), consistent with a null hypothesis of no difference. A secondary analysis was conducted using an identical methodology but using the Risk relevance score only. Risk relevance scores of 4 or 5 were included.

### Encoding of ARGs and ARG-carrying MGEs for machine learning

The following encoding pipeline was developed to encode AMR genomic traits as ML features and to capture the co-occurrence of multiple resistance mechanisms, including chromosomal resistance, IS-mediated mobility, and plasmid-associated dissemination, and their additive effects on resistance phenotype. Since the same ARGs often appeared in different isolates within the same pathogen, but within diverse ISs and plasmid configurations (Supplementary Data 2 and Fig. S1), we sought to prevent their correlation with the resistance phenotypes from being diluted by these variations. We therefore set up an encoding strategy to comprehensively account for the contribution of each ARG in the genome, accounting for its possible different genomic configurations. Three types of features were selected as ML inputs: ARG presence (including fluoroquinolone resistance mutations), ARG-carrying ISs, and ARG-carrying plasmids.

For each ARG, all possible configurations, when present, were considered and fed simultaneously to the models to capture additive and synergistic effects.

First, if an ARG was identified in the genome, it was included as an input feature, regardless of its location, encoded as binary presence/absence, with genome location considered separately in a *post hoc* analysis.

Second, if an ARG was present in the genome, we accounted for mobility-associated features by uniquely identifying ARG-carrying ISs in each isolate based on their specific IS-ARG combinations, as described above.

Third, to account for plasmid-associated AMR, ARG-containing plasmids were also included as input features. Given the large number of ARGs and the high diversity of ARG-plasmids combinations, encoding each ARG-plasmid combination separately, would have produced many plasmid-associated features per isolate, potentially diluting the correlation of each ARG with the resistance phenotypes, and introducing a dimensionality issue, possibly leading to ML model instability. To address this, we assessed whether each ARG-containing isolate also carried an associated plasmid harbouring the same ARGs as the feature value.

After annotation of ARGs and ARG- carrying MGEs, genomic features were numerically encoded for downstream analysis (machine learning and statistics), as follows: the list of *m* unique ARGs was encoded as binary absence/presence per isolate; the list of *n* unique ARG-carrying ISs was similarly encoded as binary absence/presence per isolate; and the list of *k* unique ARG-carrying plasmids was encoded as an integer sequence, with each integer representing the number of ARGs per plasmid per isolate. ARG-carrying plasmid features were min-max normalised to rescale them to the 0–1 interval. The three feature types were then concatenated into a single list of encoded feature vector per isolate, represented as an (*n* + *m* + *k)* list of scalars. Features exhibiting the same value across all isolates (invariant features), were excluded, as they lacked discriminating power; this resulted in a shorter final list of genome features.

### Population structure analysis

Population structure was inferred using PopPUNKv2.7.6^56^. Separate MDR and non-MDR sequence databases were built from genome assemblies using default parameters. A PopPUNK model was fitted using Hierarchical Density-Based Spatial Clustering of Applications with Noise (HDBSCAN) (--fit-model dbscan) and refined (--fit-model refine), and isolates were assigned to PopPUNK clusters based on the refined model. Cluster membership was incorporated as a random intercept in all mixed-effects models to explicitly control for lineage structure.

After comparing separate and joint (combining MDR and non-MDR) PopPUNK models, separate PopPUNK models were constructed for the MDR and non-MDR datasets. Combining datasets substantially reduced cluster resolution (MDR: 141–83 clusters; non-MDR: 31–29 clusters), with genuine lineages collapsing into coarser groupings, and resulted in cluster definitions being dominated by the larger MDR dataset (*n* = 2536) relative to the non-MDR dataset (*n* = 306). As PopPUNK cluster membership was used as a random effect in all GLMM models to control for population structure, separate models were retained to preserve fine-scale lineage resolution independently for each dataset. PopPUNK clustering incorporates accessory genome distances in addition to core genome distances, capturing structure not visible in the core-genome phylogeny used for visualisation.

### Machine learning

Input features: For each antibiotic-specific model, the input features comprised encoded AMR genomic traits derived from whole-genome sequence data, including ARG presence/absence, ARG-carrying insertion sequence (IS) patterns, and ARG-carrying plasmid content, as described above (see Encoding of ARGs and ARG-carrying MGEs). The outcome variable for each model was the binary phenotypic resistance or susceptibility of each isolate to the corresponding antibiotic, as determined independently by antimicrobial susceptibility testing (AST) using CLSI M100 30th Edition breakpoints (see Antimicrobial susceptibility testing). Genomic features and phenotypic outcomes therefore represent entirely independent data modalities. In summary, model evaluation followed a stratified group k-fold cross-validation (*k* = 5) framework, with folds grouped by PopPUNK cluster to prevent population structure leakage, repeated across 50 independent trials. Performance was assessed using ROC-AUC, PR-AUC, accuracy, sensitivity, specificity, and precision, reported as mean and standard deviation across folds and trials. Only models achieving AUC > 0.7 were retained. Within retained models, genomic features were selected based on three criteria applied jointly: effect size (OR ≥ 2), statistical significance (lower 95% CI ≥ 1), and cross-validation stability (positive association with resistance in ≥80% of trials). This is detailed below.

Generalised Bayesian mixed-effects logistic regression models (GLMM) were used to predict resistance or susceptibility, incorporating population structure, geography, sampling time, and host as random effects to account for lineage structure and sampling bias, Fig. S2. A Bayesian approach was chosen over standard GLMs as it shows better inference stability when sample sizes in the minority class are small^57^. To exclude the possibility that extreme class imbalance could result in unstable models, antibiotic models where fewer than 10% of samples fell in one class were excluded from modelling. Province, host, collection year and PopPUNK cluster were encoded as categorical random effects. The model was fitted using BinomialBayesMixedGLM from the statsmodels Python package, with genomic features modelled as fixed effects and province, host, year, and PopPUNK cluster modelled as random effects. Interclass correlation coefficients (ICCs) were calculated to quantify the proportion of model variance attributable to each of these. ICC for each random effect *k* were calculated as

5 $${{ICC}}_{k}=\frac{{\sigma }_{k}^{2}}{{\sum }_{j}{\sigma }_{j}^{2}+\frac{{\pi }^{2}}{3}}$$

where ${\sigma }^{2}$ represents the variance, *j* is the set of all random effects, and the fixed logistic residual variance is calculated as $\frac{{\pi }^{2}}{3}$.

To manage the high-dimensionality of the feature space while avoiding introducing selection bias and maintaining biological traceability, we avoided explicit dimensionality reduction or feature selection. Instead, regularisation was implemented via a Gaussian prior on fixed effects (equivalent to an L2/Ridge penalty of 2.0) and a log-normal prior on variance components (penalty of 0.5), limiting large coefficients and compensating for collinearity. This shrinkage approach was specifically chosen over L1/LASSO regularisation to ensure stability in the presence of high collinearity; unlike LASSO, which may arbitrarily remove one of two highly correlated genomic features, our Bayesian Ridge framework shrinks coefficients proportionately, preserving biological context and traceability.

To ensure the robustness of associations in a combinatorial feature space, features were only prioritized for forecasting if they demonstrated stability across 50 trials of stratified cross-validation (positive OR in ≥80% of trials).

No further arbitrary feature selection was applied, ensuring that all retained predictors remain directly traceable to the original genomic data.

Model performance was evaluated using stratified group k-fold cross-validation (*k* = 5), with folds grouped by PopPUNK cluster to prevent population structure leakage. This is a more stringent evaluation than standard random cross-validation^58^, as it assesses performance across lineage boundaries rather than within them. Where fewer than five clusters were present, the number of folds was reduced accordingly. The cross-validation (CV) procedure was repeated across 50 trials to obtain robust estimates of performance and feature importance. Within each fold, models were trained on the training set and evaluated on the held‑out test set. Folds in which both resistant and susceptible cases were absent in either set were excluded.

Performance was assessed using the held-out fold in each trial, using the Receiver Operating Characteristic Area Under the Curve (ROC-AUC), precision-recall AUC (PR-AUC), accuracy, specificity, and precision, and reported as mean and standard deviation across all folds and trials. Confidence intervals were calculated from cross-validation output, and final per feature estimates of confidence intervals were obtained by averaging across folds and trials.

To obtain final stable estimates for genomic features importance, a final model was fitted using all data; genomic feature importance was quantified using odds ratios, ${OR}={e}^{\beta }$ derived from a standard transformation of the fixed-effects coefficients. Traits with a model AUC > 0.7 were retained. Features within retained models were kept only if all of the following criteria were met:

Effect size: an odds ratio (OR) ≥ 2.0 in the final fitted model, ensuring biologically meaningful effect magnitude^59^.

Statistical significance: a lower 95% confidence interval ≥ 1, equivalent to a *p*-value below 0.05.

Cross-validation stability (≥80% CV retention): Features were required to show a positive association with resistance in > 80% of 50 cross-validation trials (averaged over folds per trial), ensuring robustness to sampling variability and preventing sparse or unstable associations from being propagated.

#### External validation analyses

A within-group validation analysis was conducted using isolates from 2021 and 2022 as a hold-out set. Models were fitted on the remaining (pre-2021) isolates, and the fitted coefficient estimates were applied to the hold-out set to obtain predictive performance estimates without refitting. Similarly, an independent out-of-group dataset was retrieved, comprising human and food *Salmonella* isolates collected in Guangdong province between 2017 and 2024^21^, including diarrhoeal patients from sentinel hospitals and food sources comprising pre-packaged ready-to-eat foods, cooked foods, pastry products, baked goods, pre-prepared dishes, and vegetables. Models were fitted on the original (full) dataset, and the fitted coefficient estimates were applied to the out-of-group dataset to obtain predictive performance estimates.

#### Assessment of serotype bias

To assess dominant-serovar bias in the ML-selected features, a dominance enrichment score per trait was calculated as the fraction of trait occurrences in the dominant serovars divided by the corresponding fraction of isolates in the dominant serovars. The median dominance trait score across selected traits was compared to the median dominance trait score across none selected traits. Dominant serovars were defined at those comprising ≥ 5% of isolates in the dataset. Very rare serovars (present in ≤ 2 isolates) that would bias the analysis were filtered out.

#### Forecasting analysis

We developed a pipeline to identify socioeconomic, environmental, and health indicators strongly correlated with the prevalence of AMR traits identified by the ML framework, and to forecast changes in trait prevalence through 2050, Fig. S3. The pipeline comprises seven steps:

1. Indicators were obtained from multiple sources (World Bank Data^60^, ResistanceMap^61^, Meteostat data^62^, Wushouer et al.^63^, National Bureau of Statistics of China^42^ and Global Health Data Exchange^64,65^), including antibiotic consumption, socioeconomic information, mortality, health-related, governance, and climate data collected for China, Supplementary Data 13.
2. Individual antimicrobial susceptibility tests (ASTs) were grouped into antibiotic classes, and AMR traits assigned to each AST were aggregated into a single set. Trait prevalence for each genomic feature selected by ML was calculated as the number of resistant isolates carrying the AMR trait relative to the total number of resistant and susceptible isolates. Notably, prevalence was calculated at the antibiotic class level, by aggregating resistant and susceptible isolates for all the antibiotics within the same class. Years with fewer than six isolates were excluded from the prevalence calculation to avoid instability from small sample size.
3. Pearson correlation coefficients were computed between the prevalence of each AMR trait and each candidate indicator. To ensure reliability, correlations were only calculated where at least five years of overlapping indicator and AMR trait prevalence data were available. Overlapping data spanned a minimum of six and a maximum of 17 years. Only pairs with a Pearson coefficient *r* > │0.5│were retained for further analysis.
4. To address autocorrelation in indicator data, each indicator was subjected to an ADF and Engle-Granger cointegration tests to assess stationarity and cointegration. Indicators showing no co-integration or inconsistent stationarity were first order differenced.
5. For each retained pair, linear regression was fitted to model the relationship between AMR trait prevalence and each indicator value, enabling subsequent prediction of prevalence from forecasted indicator values.
6. To control for false positives arising from large-scale testing of genomic features across multiple indicators, we applied a permutation-based significance testing procedure combined with Benjamini–Hochberg FDR correction. For each indicator–feature regression, the response variable was randomly permuted 1,000 times to generate an empirical null distribution, from which permutation-based *p*-values were derived.
  Permutation-derived *p*-values were adjusted using the Benjamini–Hochberg procedure in the statsmodels Python package, with an FDR threshold of 10% (adjusted *p*-values ≤ 0.1). Only pairs remaining significant after FDR correction were retained. FDR correction was applied within each antibiotic class model rather than globally across all tests, as tests within each model share a common biological context while tests across different antibiotic classes are largely independent.
7. Time-trend models were then fitted to each retained indicator using the available annual data. Curve fitting considered either a linear function or one of three non-linear forms (Hill logistic growth, exponential growth, or Gompertz growth), with the best fit selected by highest *R*² (minimum threshold *R*² > 0.8). Other common non-linear forms such as polynomials or splines were considered unsuitable for long term forecasting. To ensure forecasting robustness to the choice of functional forms, we verified that all plausible functions (*R*^2^ ≥ 0.8) predicted qualitatively consistent behaviour: the direction of the slope was checked at 10th, 50th and 90th percentiles of the time axis (requiring 80% consistency), and the average spread of fitted curves—measured as mean standard deviation normalised by the observed range—was required to be ≤ 0.1. Overall, 99% of indicators showed stable behaviour across functional forms, supporting the robustness of the approach. The best fit model for each indicator was then used as a starting point for a Monte Carlo simulation (10,000 iterations) to project indicator values until 2050, reported as the mean and 5th and 95th percentile range across simulations.
8. Forecasted indicator values then passed to the linear models from step (5) to project AMR trait prevalence through 2050, again using Monte Carlo simulation, and reported as the mean and 5th–95th percentile range.
9. For each indicator-trait both time series were also tested for stationarity using the ADF (Augmented Dickey-Fuller) and KPSS (Kwiatkowski-Phillips-Schmidt-Shin) test statistics^66,67^. The ADF tests rejects the hypothesis of nonstationary, while the KPSS test rejects the hypothesis of stationary. A *p-*value of 0.05 was applied to both tests. Evidence of non-stationarity strengthened confidence in forecasts projecting increasing or decreasing AMR trait prevalence by 2050.

#### Indicator-indicator correlation analysis

To assess the degree of multicollinearity among the 96 indicators used in the forecasting pipeline, pairwise Pearson correlations were calculated between all indicator pairs in Python SciPy v1.15.2. To address autocorrelation, each indicator pair was first subjected to ADF stationarity testing and Engle-Granger cointegration testing. Pairs where both indicators were non-stationary and not cointegrated (*p*-value ≥ 0.05) were first-order differenced prior to correlation calculation; otherwise original data were retained. Pairwise correlations were visualised as a clustered heatmap with hierarchical clustering based on absolute correlation coefficients |*r*| , to identify clusters of co-varying indicators regardless of the direction of association. Indicators were also grouped into indicator clusters based on the strength of their correlation (|*r*| > 0.5).

#### Indicator category breadth analysis

For each forecastable AMR trait, the number of distinct indicator categories (socioeconomic, health-related, antibiotic consumption, climate, governance) represented among its significantly associated indicators was calculated from Supplementary Data 13. Similarly, for each forecastable AMR trait, the number of distinct indicator clusters was calculated. Traits were classified as associated with a single indicator category/cluster or with multiple indicator categories/clusters to assess whether projected trajectories were supported by convergent evidence across independent indicator domains.

#### Sensitivity analysis: representative indicators per cluster

For each correlated indicator cluster identified in the indicator-indicator correlation analysis, a single representative indicator was selected based on the highest absolute Pearson correlation with AMR trait prevalence. The full forecasting pipeline was then re-assessed using only these representative indicators, and the resulting set of forecastable traits and their projected directions were compared with those from the main analysis to assess robustness to indicator redundancy.

#### Principal component analysis of indicator space

To characterise the latent structure of indicator co-variation, principal component analysis (PCA) was conducted across the 96 indicators using time-series preprocessing described above (stationarity testing and first-order differencing where appropriate). The number of components retained was determined by cumulative variance. Component loadings were examined to characterise the underlying socioeconomic, health, and environmental dimensions represented by each component. The association between forecastable AMR-trait indicator pairs and principal component loadings was assessed by mapping the AMR-trait associated indicators onto the PCA loading structure, to calculate the fraction of each PCA component associated to each trait. Permutation testing was used to randomly permute AMR trait labels 5000 times to generate a null distribution, testing whether the observed trait-component profiles showed greater specificity to any particular principal component than would be expected by chance.

#### Visualisation

Maps were created using a public domain map (standard map approval number: GS(2020)4624) from http://bzdt.ch.mnr.gov.cn/, accessed 01-01-25. Undirected graphs were created using NetworkX v2.8.4^68^ and visualised using Gephi v0.10.1. Sankey diagrams were created using Plotly v6.0.0^69^.

#### Statistical analysis

Statistical comparisons within the ML pipeline were performed using the SciPy v1.15.2 unless otherwise stated, implementing: (1) a two-sided Cramér’s *V* test to evaluate serovar and ST correlation (IBM SPSS Statistics v29.0.1.0); (2) a two-sided Kruskal–Wallis test to assess ARG location distribution across source types; (3) a two tailed Kruskal–Wallis test with post hoc FDR corrected Mann–Whitney *U* tests to assess differences in feature Chao1 feature richness across source types - Chao1 richness was calculated both across all source types and restricted to source types with ≥ 100 samples to test robustness; both measures were in agreement; (4) a two-sided chi-squared test to evaluate similarity between AMR features and AMR phenotypic profiles across different antibiotics (*p-*value  <  0.05); (5) a two-sided Kruskal–Wallis test to assess the distribution of high-risk traits across source types; (6) a two-tailed proportion *Z*-test to assess differences in thigh-risk traits in food workers versus food sources; and (7) Pearson correlations were calculated using SciPy in Python.

### Reporting summary

Further information on research design is available in the Nature Portfolio Reporting Summary linked to this article.

## Supplementary information

Supplemetary Information

Description of Additional Supplementary Files

Supplementary Data 1

Supplementary Data 2

Supplementary Data 3

Supplementary Data 4

Supplementary Data 5

Supplementary Data 6

Supplementary Data 7

Supplementary Data 8

Supplementary Data 9

Supplementary Data 10

Supplementary Data 11

Supplementary Data 12

Supplementary Data 13

Reporting Summary

Transparent Peer Review file

## Source data

Source Data

## Data Availability

The data generated for this study have been deposited in NCBI under accession codes PRJNA1172187PRJNA1171133, and PRJNA1171177. Public datasets are available in the National Genomics Data Center under Bioproject CRA012442, and NCBI under Bioprojects PRJNA1082900PRJNA841813, and PRJNA981225. All accessions and metadata are given in Supplementary Data 1. The compiled indicator data are available in Supplementary Data 10. Source data are provided with this paper.

## Code availability

The code used in this study is available in the following GitHub repository: https://github.com/tan0101/SalmonellaChina, and archived on Zenodo https://doi.org/10.5281/zenodo.21931547^70^.
