# Supplementary material for: High-risk antimicrobial resistance genomic traits in multidrug-resistant Salmonella across China’s food chain: projections to 2050
Source: Nat Commun. 2026 Oct 20;17:10744. doi: 10.1038/s41467-026-78346-7 (PMC13683381; doi:10.1038/s41467-026-78346-7)
Supplement: Supplementary file 1 [file 41467_2026_78346_MOESM16_ESM.pdf]

nature portfolio

Corresponding author(s): Tania Dottorini

Last updated by author(s): Aug 14, 2026

Reporting Summary

Nature Portfolio wishes to improve the reproducibility of the work that we publish. This form provides structure for consistency and transparency in reporting. For further information on Nature Portfolio policies, see our Editorial Policies and the Editorial Policy Checklist.

Statistics

For all statistical analyses, confirm that the following items are present in the figure legend, table legend, main text, or Methods section.

n/a	Confirmed	
☐	☒	The exact sample size (*n*) for each experimental group/condition, given as a discrete number and unit of measurement
☐	☒	A statement on whether measurements were taken from distinct samples or whether the same sample was measured repeatedly
☐	☒	The statistical test(s) used AND whether they are one- or two-sided<br>*Only common tests should be described solely by name; describe more complex techniques in the Methods section.*
☒	☐	A description of all covariates tested
☐	☒	A description of any assumptions or corrections, such as tests of normality and adjustment for multiple comparisons
☐	☒	A full description of the statistical parameters including central tendency (e.g. means) or other basic estimates (e.g. regression coefficient) AND variation (e.g. standard deviation) or associated estimates of uncertainty (e.g. confidence intervals)
☐	☒	For null hypothesis testing, the test statistic (e.g. *F*, *t*, *r*) with confidence intervals, effect sizes, degrees of freedom and *P* value noted<br>*Give P values as exact values whenever suitable.*
☒	☐	For Bayesian analysis, information on the choice of priors and Markov chain Monte Carlo settings
☒	☐	For hierarchical and complex designs, identification of the appropriate level for tests and full reporting of outcomes
☒	☐	Estimates of effect sizes (e.g. Cohen's *d*, Pearson's *r*), indicating how they were calculated

Our web collection on statistics for biologists contains articles on many of the points above.

Software and code

Policy information about availability of computer code

Data collection	No software was used for data collection
Data analysis	The code developed and used by us to analyse the data in this study has been made open source and is available on Github:<br>https://github.com/tan0101/SalmonellaChina<br>Fastp<br>Spades v3.15.5<br>Quast v5.0.2<br>CheckM v1.2.2<br>Bakta v1.4.0<br>Panaroo v1.5.0<br>Abricate v1.0.1<br>MLST v2.23.0<br>SISTR v1.0.2<br>Emboss Transeq v6.6.0.0<br>plasmidVerify v2.1.6<br>Plasmer v23.04.20<br>MOB-suite v3.1.7<br>Minimap2 v2.30<br>IQ-TREE v2.1.4

PopPUNK v2.7.6
Statmodels v v0.14.5
NetworkX v2.8.4
Gephi v0.10.1
Plotly v6.0.0
SciPy v1.15.2
IBM SPSS Statistics v29.0.1.0

The software listed above were properly mentioned and cited in the manuscript.

For manuscripts utilizing custom algorithms or software that are central to the research but not yet described in published literature, software must be made available to editors and reviewers. We strongly encourage code deposition in a community repository (e.g. GitHub). See the Nature Portfolio guidelines for submitting code & software for further information.

Data

Policy information about availability of data

All manuscripts must include a data availability statement. This statement should provide the following information, where applicable:

- Accession codes, unique identifiers, or web links for publicly available datasets
- A description of any restrictions on data availability
- For clinical datasets or third party data, please ensure that the statement adheres to our policy

Data availability
The data generated for this study have been deposited in NCBI under accession codes PRJNA1172187 (https://www.ncbi.nlm.nih.gov/bioproject/PRJNA1172187/), PRJNA1171133 (https://www.ncbi.nlm.nih.gov/bioproject/PRJNA1171133) and PRJNA1171177 (https://www.ncbi.nlm.nih.gov/bioproject/PRJNA1171177). Public datasets are available in the National Genomics Data Center under Bioproject CRA012442 (https://ngdc.cncb.ac.cn/gsa/browse/CRA012442), and NCBI under Bioprojects PRJNA1082900 (https://www.ncbi.nlm.nih.gov/bioproject/PRJNA1082900), PRJNA841813 (https://www.ncbi.nlm.nih.gov/bioproject/PRJNA841813) and PRJNA981225 (https://www.ncbi.nlm.nih.gov/bioproject/PRJNA981225). All accessions and metadata are given in Supplementary Data 1. The complied indicator data are available in Supplementary Data 10. Source data are provided with this paper.

Code availability
The code used in this study is available in the following GitHub repository: https://github.com/tan0101/SalmonellaChina and archived on Zenodo https://doi.org/10.5281/zenodo.21931547

Research involving human participants, their data, or biological material

Policy information about studies with human participants or human data. See also policy information about sex, gender (identity/presentation), and sexual orientation and race, ethnicity and racism.

Reporting on sex and gender	For the public and new data in this study, we did not use or report on sex, gender or age. The participants were not compensated, this study was part of an occupational health screening.
Reporting on race, ethnicity, or other socially relevant groupings	For the new data in this study, participants were healthy individuals, including both food workers and non food workers. Food worker and non-food worker classification followed the definitions established under Article 45 China's Food Safety Law (2015), which distinguishes workers based on whether they enter in contact with food destined to direct consumption*. Classification was based on the recorded presence or absence of a food handler health certificate. Both groups were drawn from the same occupational health screening programme and therefore underwent equivalent health surveillance, differing specifically in their occupational exposure to food.<br><br>*US Department of Agriculture Foreign Agricultural Service. English translation of China's Food Safety Law (2015). GAIN Report Number CH15016. Beijing: USDA FAS (2015).
Population characteristics	The only information we have for the participants is that the participants were healthy individuals, including both food workers and non food workers. Food worker and non-food worker classification followed the definitions established under Article 45 China's Food Safety Law (2015), which distinguishes workers based on whether they enter in contact with food destined to direct consumption*. Classification was based on the recorded presence or absence of a food handler health certificate. Both groups were drawn from the same occupational health screening programme and therefore underwent equivalent health surveillance, differing specifically in their occupational exposure to food.<br><br>*US Department of Agriculture Foreign Agricultural Service. English translation of China's Food Safety Law (2015). GAIN Report Number CH15016. Beijing: USDA FAS (2015).
Recruitment	Participants were recruited based on occupational health screening of healthy individuals, including both food workers and non food workers. Food worker and non-food worker classification followed the definitions established under Article 45 China's Food Safety Law (2015), which distinguishes workers based on whether they enter in contact with food destined to direct consumption*. Classification was based on the recorded presence or absence of a food handler health certificate. Both groups were drawn from the same occupational health screening programme and therefore underwent equivalent health surveillance, differing specifically in their occupational exposure to food.

	*US Department of Agriculture Foreign Agricultural Service. English translation of China's Food Safety Law (2015). GAIN Report Number CH15016. Beijing: USDA FAS (2015).
Ethics oversight	This study was approved by The Ethics Committee of the National Institute for Communicable Disease Control and Prevention, Chinese Center for Disease Control and Prevention (Approval Number: ICDC-2022001). A waiver of informed consent was granted due to the retrospective nature of the study

Note that full information on the approval of the study protocol must also be provided in the manuscript.

Field-specific reporting

Please select the one below that is the best fit for your research. If you are not sure, read the appropriate sections before making your selection.

[x] Life sciences [ ] Behavioural & social sciences [ ] Ecological, evolutionary & environmental sciences

For a reference copy of the document with all sections, see nature.com/documents/nr-reporting-summary-flat.pdf

Life sciences study design

All studies must disclose on these points even when the disclosure is negative.

Sample size	No a priori sample size calculation was performed, as this was an observational/exploratory study using retrospectively collected genomic surveillance data. The final dataset (2,536 MDR Salmonella enterica genomes from 15 provinces over 16 years) represents the complete set of genomes available meeting inclusion criteria / all genomes collected through surveillance programmes during this period (2006-2022). This sample size is sufficient to support the machine learning and regression analyses performed, given the number of high-risk traits examined, and stability of the performance metrics and odds ratio confidence intervals achieved.
Data exclusions	For food workers and non food workers individuals younger than 18 years, or older than 65 years, or with diarrhea, or had taken any antibiotics in the week prior to the faecal sampling were excluded.
Replication	n/a - This study was based on observational genomic surveillance data rather than controlled experiments, so biological replication was not applicable. Reproducibility of the ML computational analyses was instead verified through 50 trials of 5-fold grouped cross-validation, and forecasting through 10000 Monte Carlo simulations, ensuring the robustness of the machine learning models and regression findings.
Randomization	There were no experimental groups, as this was an observational study using retrospectively collected genomic surveillance data rather than a controlled experimental design.Covariates (geography, bacterial population structure and collection year) were used as random effected in the models to control for these factors.<br><br>For the data collection: Food worker and non-food worker classification followed the definitions established under Article 45 China's Food Safety Law (2015), which distinguishes workers based on whether they enter in contact with food destined to direct consumption. The health examination is done each year. Classification was based on the recorded presence or absence of a food handler health certificate. Both groups were drawn from the same occupational health screening programme and therefore underwent equivalent health surveillance, differing specifically in their occupational exposure to food.<br><br>For the ML classification: the samples were assigned to training and testing groups using a grouped stratified nested cross validation procedure (50 iterations).
Blinding	There were no allocated groups in this study design, as it is an observational genomic surveillance study rather than a controlled experiment. Blinding to group allocation was therefore not relevant.

Reporting for specific materials, systems and methods

We require information from authors about some types of materials, experimental systems and methods used in many studies. Here, indicate whether each material, system or method listed is relevant to your study. If you are not sure if a list item applies to your research, read the appropriate section before selecting a response.

Materials & experimental systems

n/a	Involved in the study
[x]	[ ] Antibodies
[x]	[ ] Eukaryotic cell lines
[x]	[ ] Palaeontology and archaeology
[x]	[ ] Animals and other organisms
[x]	[ ] Clinical data
[x]	[ ] Dual use research of concern
[x]	[ ] Plants

Methods

n/a	Involved in the study
[x]	[ ] ChIP-seq
[x]	[ ] Flow cytometry
[x]	[ ] MRI-based neuroimaging

Plants

Seed stocks	*Report on the source of all seed stocks or other plant material used. If applicable, state the seed stock centre and catalogue number. If plant specimens were collected from the field, describe the collection location, date and sampling procedures.*
Novel plant genotypes	*Describe the methods by which all novel plant genotypes were produced. This includes those generated by transgenic approaches, gene editing, chemical/radiation-based mutagenesis and hybridization. For transgenic lines, describe the transformation method, the number of independent lines analyzed and the generation upon which experiments were performed. For gene-edited lines, describe the editor used, the endogenous sequence targeted for editing, the targeting guide RNA sequence (if applicable) and how the editor was applied.*
Authentication	*Describe any authentication procedures for each seed stock used or novel genotype generated. Describe any experiments used to assess the effect of a mutation and, where applicable, how potential secondary effects (e.g. second site T-DNA insertions, mosiacism, off-target gene editing) were examined.*
