# Supplementary material for: High-risk antimicrobial resistance genomic traits in multidrug-resistant Salmonella across China’s food chain: projections to 2050
Source: Nat Commun. 2026 Oct 20;17:10744. doi: 10.1038/s41467-026-78346-7 (PMC13683381; doi:10.1038/s41467-026-78346-7)
Supplement: Supplementary file 2 [file 41467_2026_78346_MOESM17_ESM.pdf]

nature portfolio

Peer Review File

High-risk antimicrobial resistance genomic traits in multidrug-resistant *Salmonella* across China's food chain: projections to 2050

Corresponding Author: Professor Tania Dottorini

This file contains all reviewer reports in order by version, followed by all author rebuttals in order by version.

Version 0:

Reviewer comments:

Reviewer #1

(Remarks to the Author)
This manuscript presents a comprehensive and well-executed investigation of the spread of antimicrobial resistance (AMR) in multidrug-resistant Salmonella enterica across China's food chain. In particular, the identification of high-risk AMR traits based on clinical relevance, multidrug resistance potential, and mobility is highly meaningful and provides substantial value for prioritizing surveillance targets. The projection analyses extending to 2050 provide a useful long-term perspective and may help inform future surveillance strategies and public health planning. However, several concerns should be addressed before the conclusions can be fully supported.

1.Although the study includes isolates collected from 15 provinces across China, the sampling design appears highly unbalanced across source categories, geographic regions, and years. For example, food worker isolates constitute the majority of human samples and were collected exclusively from Guangxi Province, whereas other source categories were derived from different provinces and surveillance periods. Consequently, source-specific differences in AMR trait prevalence may be partially confounded by geographic or temporal variation. This limitation should be discussed more explicitly, particularly given its potential impact on AMR prevalence estimates, hotspot identification, and forecasting analyses.
2.The observation that food worker isolates harbour a disproportionately high burden of high-risk AMR traits is one of the most interesting findings of the study. However, the finding that more high-risk traits (Rank IV and V) occurred in food worker isolates, and that their prevalence exceeded that observed in retail meat isolates, may be influenced by differences in sampling intensity and study design across source categories. It therefore remains unclear to what extent the observed enrichment reflects characteristics of food workers specifically, broader human-associated AMR patterns, or regional epidemiological differences.
3.The forecasting framework appears to be based primarily on pairwise associations between individual indicators and AMR trait prevalence. While this approach is transparent and easy to interpret, many of the examined indicators (e.g. healthcare expenditure, urbanisation, population growth, and antimicrobial consumption) are likely to exhibit strong correlations with one another and may reflect broader underlying socioeconomic trends. As a result, some reported associations may be difficult to disentangle and could reflect shared temporal patterns rather than direct relationships with AMR trait prevalence.

(Remarks on code availability)

Reviewer #2

(Remarks to the Author)
This manuscript describes a large genomic study of antimicrobial resistance determinants in Salmonella enterica from multiple sources across the food chain in China, which was leveraged along with socioeconomic, environmental, and health indicator data to develop a predictive model for AMR traits that projects the prevalence of these traits to 2050. The methods are meticulously described and the bioinformatic and modeling parameters chosen are well thought-out and adequately defended and evaluated. The results provide a comprehensive assessment of the current AMR landscape in S. enterica in China, and a detailed forecast of AMR traits in this species in relation to demographic and environmental indicators.

Please see below for specific comments and questions related to this manuscript:

1. The authors might consider re-wording the title to avoid using the acronyms AMR and MDR prior to their definition.

2. Is it known what occupations the non-food workers sampled held? Were all of them at low risk of occupational exposure to Salmonella?

3. In the bioinformatic section of the methods (starting on Line 156), it is unclear which methods in the analysis were applied only to the newly sequenced isolates from this study and which were applied to the previously sequenced publicly available sequences.

4. The annotation of assemblies with Abricate for ARG and IS identification is described 3 times in the Methods section; the authors might consider removing this redundancy.

5. How were antimicrobial susceptibility data from previously sequenced publicly available isolates retrieved? Did the interpretations all use the same M100 30th Edition breakpoints as the new isolates from this study?

6. Given the overlap of MDR and non-MDR isolates in the maximum likelihood phylogenetic tree, why were separate PopPUNK models used for MDR and non-MDR isolates?

7. In line 543, it is stated that a higher proportion of high-risk traits occurred in food worker vs. food isolates. However, AMR traits present only in human sources were by definition assigned a higher inter-host mobility score, and therefore a higher risk score, than traits present only in food sources, so this may be in part an artificially imposed association.

8. There are only single values for the socioeconomic, environmental, and health indicator data used to represent all of China – do the authors have insight into how much these indicators vary geographically within the country, and whether this might affect the results of the study?

9. As many of the AMR traits characterized in this study are transferrable across bacterial species, do the authors think that any of the forecasting results might be applicable to future AMR trends in other foodborne bacteria?

10. What specific added value does the gene- and indicator-level AMR prediction provided in this study have over more generic class-based risk assessments in terms of clinical, public health, or policy decisions?

(Remarks on code availability)
The code appears to fully reproduce the results of the paper, and provides a comprehensive README outlining dependencies and usage instructions.

Reviewer #3

(Remarks to the Author)
This manuscript by Baker et al. investigates high-risk AMR genomic traits in MDR Salmonella across China's food chain. The authors developed an ARG risk-ranking framework and used machine learning to project AMR trends through 2050. Although the study addresses an important topic and uses a unique large-scale dataset, several methodological limitations reduce confidence in the findings. In its current form, the analyses do not fully capture the richness of the dataset or provide the depth of insight needed to support the broader conclusions.

Major comments:
1. The ML framework is difficult to follow. Although many steps are described, the overall structure of the analysis is not sufficiently clear. In particular, the authors should clearly define the input features, outcome variables, and evaluation strategy for each model. At present, it appears that AMR genomic traits may have been included as model features, while resistance or susceptibility was treated as the outcome. If so, the authors need to explain how phenotypic resistance or susceptibility was defined and quantified. Otherwise, the analysis risks circularity, essentially using AMR-associated features to predict AMR status. The authors should clarify whether the model is predicting independent phenotypic resistance outcomes from genomic predictors, or whether it is partly using the same AMR information to predict itself. It is also not clear how training/testing was split. Importantly, a key limitation is the lack of external validation for the ML models. The current training-testing framework may not be sufficient to establish model generalizability, particularly if the same dataset was used for feature selection, model tuning, and performance evaluation.

2. The proposed ARG risk-ranking system is insufficiently justified and appears underdeveloped in relation to existing antimicrobial resistance gene risk-assessment frameworks (e.g., Zhang et al, 3032). The authors should clearly explain how this system aligns with, differs from, or improves upon prior frameworks. At present, it is difficult to determine whether the ranking provides new conceptual or practical value, or whether it largely repackages criteria that have already been established. Without a direct comparison to existing ARG risk frameworks and a transparent explanation of scoring, weighting, and validation, the proposed ranking system risks appearing subjective and difficult to reproduce. This substantially limits confidence in its novelty, rigor, and utility for AMR surveillance or risk prioritization.

3. The authors attempted to project the impact of antibiotic use, population growth, healthcare spending, and urbanization on

future AMR patterns. However, the analysis does not first provide a thorough assessment of how these factors explain the current AMR patterns observed in the dataset. This weakens the basis for long-term projection. Without demonstrating that these variables are meaningfully associated with present-day AMR variation, it is difficult to interpret their projected effects over the next 20 years. As currently presented, the forecast provides limited mechanistic or explanatory insight, because the reader is asked to accept future predictions before the drivers of current patterns have been rigorously established.

4. Although diverse Salmonella serotypes were included in the analyses, the role of serotype was not sufficiently addressed. This is an important limitation because Salmonella serotypes differ substantially in host range, host adaptation, ecological niche, food production chain association, and AMR potential. Therefore, conclusions generalized across all isolates may be biased by the serotypes that dominate the dataset. The study would be strengthened by stratifying AMR profiles by serotype and assessing whether patterns of AMR emergence, persistence, and dissemination differ across serotypes. In addition, the analysis should evaluate how environmental, host-associated, and food production-related factors interact with serotype to shape AMR profiles. Without this level of resolution, it is difficult to determine whether the reported AMR patterns reflect broad species-level trends or are driven primarily by dominant serotype-specific effects.

Other comments:
Lines 128-129: better clarify that these 279 MDR and 138 non-MDR isolates were newly acquired isolates.
Lines 128-148: The authors should clarify how they verified the phenotypic resistance status of MDR and non-MDR isolates obtained from public sources. Specifically, were these classifications based on publicly available antimicrobial susceptibility testing metadata, original source publications, database annotations, or independent reanalysis?
Lines 156-161: what assembly quality metrics (e.g., N50, number of contigs), genome completeness and contamination cutoffs were used to ensure that all genomes (not just for these new isolates but all genomes included in the analysis) have high quality?
Line 163: what is the difference between the phylogeny inferred here different from the one descripted in Lines 219-227?
Lines 163-165: This description seems redundant, as the ARG profiling methods are described in detail below.
Lines 277: what is a and what is m?
Lines 282-283: what is the difference between mobility and HGT? How was HGT detected and quantified? Also, HGT needs to be spelled out. This is the first time it shows up.
Line 502: P values cannot truly be 0, so the reported values may reflect rounding or numerical precision limitations. The authors should revise these values to an appropriate format, such as $P<0.001$, depending on the actual precision.

(Remarks on code availability)
The code for the bioinformatics analyses is largely missing, whereas the code for the machine learning models is provided in detail.

Version 1:

Reviewer comments:

Reviewer #1

(Remarks to the Author)
The authors have satisfactorily addressed the concerns regarding sampling bias and the interpretation of forecasting associations. The revised manuscript appropriately acknowledges the potential impact of unbalanced sampling across source categories, geographic regions, and collection periods. The additional analyses further strengthen the robustness of the findings. Importantly, the authors have appropriately emphasized that the observed relationships are associational in nature and should not be interpreted as evidence of causality, and have revised the discussion accordingly.

(Remarks on code availability)

Reviewer #3

(Remarks to the Author)
The authors have adequately addressed my concerns.

(Remarks on code availability)

Open Access This Peer Review File is licensed under a Creative Commons Attribution 4.0 International License, which permits use, sharing, adaptation, distribution and reproduction in any medium or format, as long as you give appropriate credit to the original author(s) and the source, provide a link to the Creative Commons license, and indicate if changes were made.
In cases where reviewers are anonymous, credit should be given to 'Anonymous Referee' and the source.
The images or other third party material in this Peer Review File are included in the article's Creative Commons license, unless indicated otherwise in a credit line to the material. If material is not included in the article's Creative Commons license and your intended use is not permitted by statutory regulation or exceeds the permitted use, you will need to obtain permission directly from the copyright holder.
To view a copy of this license, visit https://creativecommons.org/licenses/by/4.0/

Dear Editor,

We thank the Editor and Reviewers for their useful comments, which no doubt contributed to make this a better paper. In the following, a point-by-point response to all the questions and comments raised by the Editor and the Reviewers is provided. The original questions are in blue, our replies in black. Lines numbers cited in this document refer to the tracked changes version of the revised manuscript (Manucript_trackedchanges.pdf). Please note that in the revised manuscript the Methods section has been moved to after the Discussion section, aligning to Nature Communications article guidelines, this move was not done is tracked changes to ensure other changes were visible.

Reviewer #1 (Remarks to the Author)

This manuscript presents a comprehensive and well-executed investigation of the spread of antimicrobial resistance (AMR) in multidrug-resistant Salmonella enterica across China's food chain. In particular, the identification of high-risk AMR traits based on clinical relevance, multidrug resistance potential, and mobility is highly meaningful and provides substantial value for prioritizing surveillance targets. The projection analyses extending to 2050 provide a useful long-term perspective and may help inform future surveillance strategies and public health planning. However, several concerns should be addressed before the conclusions can be fully supported.

1.Although the study includes isolates collected from 15 provinces across China, the sampling design appears highly unbalanced across source categories, geographic regions, and years. For example, food worker isolates constitute the majority of human samples and were collected exclusively from Guangxi Province, whereas other source categories were derived from different provinces and surveillance periods. Consequently, source-specific differences in AMR trait prevalence may be partially confounded by geographic or temporal variation. This limitation should be discussed more explicitly, particularly given its potential impact on AMR prevalence estimates, hotspot identification, and forecasting analyses.

We thank the reviewer for this important methodological observation. We agree that the unbalanced sampling design across source categories, provinces, and collection periods represents a genuine limitation that was not sufficiently explicit in the original Discussion, and we have now addressed this directly.

Our mixed-effects modelling framework includes province, year, host/source type, and PopPUNK cluster as random effects in all Bayesian mixed-effects logistic regression models, and the interclass correlation coefficients (ICCs) confirm that province (ICC = 0.149) and year (ICC = 0.164) were the two largest contributors to model variance, indicating that the models recognised and absorbed substantial geographic and temporal signal. However, we acknowledge random effects only partially absorb variation due to grouping factors and that ICC values quantify variance attribution rather than confound elimination.

Specifically, because food worker and non-food worker isolates were collected exclusively from Guangxi Province, there are no cross-province, within-source-type contrasts available to fully anchor the separation between a "food worker effect" and a "Guangxi Province effect." The mixed-effects model partially adjusts for average province-level differences, but residual confounding attributable to the non-overlapping sampling frames across source types cannot be excluded. This has direct implications for three aspects of the analysis the reviewer identifies:

AMR trait prevalence estimates: The elevated trait prevalence and richness observed in food workers may partly reflect Guangxi-specific AMR epidemiology, the intensive longitudinal nature of occupational health surveillance, or secular trends during the collection period, in addition to occupational exposure.

Hotspot identification: The designation of food workers as potential AMR hotspots is supported by a formal statistical comparison (proportion Z-test, *p*-value = 4.289×10^{-21}) conducted within models adjusting for province and year, but should be interpreted as an association rather than a source-attributable causal effect. This is also supported by an additional analysis done for this Reviewer1, please see Q2.

Forecasting: Forecasting models are calibrated against national-level indicators and cannot fully account for province-specific trajectories, which may be particularly relevant for the food worker-derived traits given their geographic concentration.

As correctly suggested by the Reviewer we have revised the Discussion to make these limitations more explicitly, particularly given their potential impact on AMR prevalence estimates, hotspot identification, and forecasting analyses, and we believe this more measured framing appropriately contextualises the food worker findings without undermining the broader analytical contributions of the study. The revised text is provided below.

Discussion Line 580-649:

A key limitation concerns the unbalanced sampling design across source categories, geographic regions, and collection periods. Food worker and non-food worker isolates were collected exclusively from Guangxi Province as part of a structured occupational health surveillance programme, whereas retail meat, livestock, farm environment, and symptomatic patient isolates were derived from different provinces and distinct surveillance windows spanning 2006–2022. This structural heterogeneity means that comparisons of AMR trait prevalence across source types are potentially confounded by geographic and temporal co-variation that cannot be fully resolved within the modelling framework.

Province, year, source type, and lineage (PopPUNK cluster) were included as random effects in all Bayesian mixed effects models and interclass correlation coefficients confirm that province (ICC = 0.149) and year (ICC = 0.164) were the largest sources of model variance, indicating that the analytical framework recognised and partially absorbed geographic and temporal signal. However, random effects only partially absorb variation due to grouping factors and ICC values reflect variance attribution rather than confound elimination. Because food worker isolates originate exclusively from Guangxi, the data contain no cross-province, within-source-type contrasts to fully disentangle a source-type effect from a province-specific effect, and residual confounding cannot be excluded.

This limitation has specific implications for three aspects of the analysis. First, source-specific AMR trait prevalence estimates, particularly the elevated trait richness and high-risk trait burden observed in food workers, may partly reflect Guangxi's regional AMR epidemiology, the intensive longitudinal nature of occupational screening, or secular trends during the years of collection, rather than being solely attributable to occupational exposure. Second, while the identification of food workers as potential AMR hotspots is supported by a formal statistical test (proportion Z-test, *p*-value = 4.289×10^{-21}) and is further supported by the bootstrapped human group comparisons reported in the Results, this test was conducted on raw proportions and does not itself adjust for province or year; the geographic and temporal confounding discussed above therefore applies directly to this comparison and should be considered when interpreting the magnitude, though not necessarily the direction, of this finding. Furthermore, this finding should be interpreted as an epidemiological association rather than a causally attributed source effect. Third, forecasting models are calibrated against national-level socioeconomic and health indicators and may not capture province-specific resistance trajectories, which is of particular relevance for traits disproportionately represented in the Guangxi food worker cohort. Future studies would benefit substantially from prospective, multi-province sampling of all source types within overlapping geographic and temporal windows, which would provide the within-province cross-source contrasts necessary for more rigorous causal inference and regionally stratified forecasting.

The study design also allowed for repeated sampling of food and non-food workers as part of routine occupational health screening. Although this is a potential source of bias in the models, its effects were minimised by ensuring all individuals testing positive were treated until they received a negative test result, reducing the likelihood of strains being sequenced multiple times, and increasing the likelihood of independent infections. However, we acknowledge that the experimental approach does not guarantee independence between isolates from the same individual. Additionally, we acknowledge that reconstructing plasmids from short-read data is inherently challenging; the tools used represent current best practice when long-read data are unavailable, and our pipeline incorporates multiple independent tools to minimise false-positive and false-negative results. Nevertheless, some inaccuracies in plasmid reconstructions are expected.

A further limitation concerns the correlational nature of all indicator-trait associations reported in the forecasting analysis. Despite the application of stationarity testing, cointegration testing, first-order differencing, and permutation-based FDR correction to mitigate spurious associations driven by shared temporal trends, the pairwise framework cannot establish causality or directionality between individual indicators and AMR trait prevalence. Some indicators reflecting broad economic development trajectories, including healthcare expenditure, urbanisation, and population growth may act in part as proxies for a shared underlying driver that cannot be fully decomposed through pairwise analyses given the constraints of national-level time series data and the limited number of annual observations available (maximum 17 years of overlap). This is an inherent limitation of ecological surveillance studies at the national level. The indicator-trait associations reported here should therefore be interpreted as identifying candidate structural factors associated with AMR trait prevalence warranting further investigation through prospective, regionally stratified study designs, rather than as evidence of direct causal relationships. Future studies incorporating subnational data, longitudinal cohort designs, or natural experiment frameworks, such as policy changes affecting antibiotic use, would enable stronger causal inference about the structural determinants of AMR spread across China's food chain.

2.The observation that food worker isolates harbour a disproportionately high burden of high-risk AMR traits is one of the most interesting findings of the study. However, the finding that more high-risk traits (Rank IV and V) occurred in food worker isolates, and that their prevalence exceeded that observed in retail meat isolates, may be influenced by differences in sampling intensity and study design across source categories. It therefore remains unclear to what extent the observed enrichment reflects characteristics of food workers specifically, broader human-associated AMR patterns, or regional epidemiological differences.

We thank the reviewer for this important observation. We agree that differences in sampling intensity and study design across source categories could influence the observed enrichment of high-risk traits in food worker isolates, and we have addressed this through three additional analyses.

Addressing sampling intensity bias: The original analysis tested whether the proportion of high-risk traits in food workers significantly differed from that in food isolates, where a trait was considered present if found in at least one isolate of that source type. This calculation does not explicitly account for sample size, larger sample sizes increase the likelihood of detecting rarer traits, which could bias the comparison. To address this, we conducted a bootstrapping analysis (10,000 iterations) in which food worker isolates were down-sampled to match the sample size of food isolates. This confirmed that food worker isolates carried a small but statistically significant higher proportion of high-risk traits compared to food isolates after sample size correction (mean difference 7%; 95% CI [3.4%, 11.3%], one-tailed bootstrap *p*-value = 0.0004). We therefore conclude that while the magnitude and statistical significance of the originally reported difference were inflated by sample size differences, the enrichment reflects a true difference between groups.

Addressing potential bias in the study design - risk assessment framework: The reviewer correctly raises the possibility that the enrichment might reflect broader human-associated AMR patterns rather than

food worker-specific characteristics, since the risk scoring criteria explicitly incorporate interhost mobility and clinical significance, which could in principle, systematically elevate risk scores for any human-derived isolates. Firstly, we assessed the proportion of high-risk AMR traits exclusively found in food workers vs those found in both food workers and food. Overall, only 35.5% of traits were found in food workers alone, compared to 64.5% that we also found in food and/or livestock. This indicates that most high-risk traits were not found in food workers only but were shared across food and/or livestock but does not account for sample size differences.

Secondly, to test this, we applied the same bootstrapped down-sampling analysis, described above, to the two other human source groups in our dataset, non-food workers and symptomatic patients, testing the hypothesis that if the risk assessment design systematically biases toward higher risk scores in human isolates, all three human groups should show elevated proportions of high-risk traits relative to food isolates. Non-food workers are a particularly informative comparator here because, like food workers, they were recruited from Guangxi Province under the same occupational health surveillance programme, simultaneously controlling for both the human-source effect and the regional epidemiological effect. Non-food worker isolates showed a slightly higher proportion of high-risk traits than food isolates after sample size correction (mean difference 4.3%; 95% CI [1.3%, 7.1%], one-tailed bootstrap *p*-value =0.002), but with a meaningfully smaller effect size than food workers (7%). Symptomatic patient isolates showed a significantly lower proportion of high-risk traits compared to food isolates after sample size correction (mean difference -5.9%; 95% CI [-9.2%, -2.5%], one-tailed bootstrap *p*-value= 0.0002). We note that the symptomatic patient comparison is weaker evidence than the non-food worker comparison, as symptomatic patients differ from food workers in province, collection period, and health/exposure status simultaneously, whereas non-food workers differ only in occupational food contact within an otherwise matched sampling frame. As a further test, food worker isolates were directly compared to non-food worker isolates (all collected as part of the same surveillance programme) removing confounding factors of host, geography, collection and years. This showed that after down-sampling, although not statistically significant, food workers had a slightly higher proportion of high-risk AMR traits compared to non-food workers (mean difference 2.35%, CI [-1.68%, 6.3%], one-tailed bootstrap *p*-value = 0.31).

The gradient of effect sizes across human source groups, food workers 7% > non-food workers 4.3% > symptomatic patients -5.9%, is inconsistent with a uniform artificial inflation of risk scores across all human sources, which would produce a similar enrichment in all three groups. Instead, this pattern is consistent with a genuine occupational exposure gradient, where food-related occupational exposure is associated with the highest burden of high-risk traits, non-food occupational exposure from the same province and surveillance setting is associated with a smaller enrichment, and symptomatic patients from other provinces show no enrichment relative to food isolates.

As third assessment, the decomposed risk score was also analysed, specifically considering the risk severity only and disregarding the risk likelihood that contained the mobility component, which was host dependent. Considering AMR traits with a risk severity score of 4 or 5, (which included only Rank I clinically significant traits and MDR traits conferring resistance to antibiotics used in both animals and humans and on the AWARE Watch or Reserve lists), revealed similar results to the previous analysis. Specifically, food worker isolates vs food isolates: mean difference +7.1% (95% CI [2.8%, 11.3%], one-tailed bootstrap *p*-value=0.0006); non-food worker isolates vs food isolates: mean difference +5.0% (95% CI [2.43%, 7.7%], one-tailed bootstrap *p*-value=0.0002); Symptomatic patient isolates vs food isolates: mean difference -5.9% (95% CI [-8.9%, -2.8%], one-tailed bootstrap *p*-value=0.0001). We therefore conclude that the food worker enrichment is unlikely to be primarily attributable to a systematic bias in the risk scoring criteria or to broader human-associated AMR patterns.

Addressing regional epidemiological differences: We acknowledge that the above analyses only partially resolve the potential contribution of regional epidemiological differences to the observed enrichment. The non-food worker comparison is the strongest available control for geographic

confounding, since non-food workers share the same Guangxi provenance and surveillance setting as food workers yet show a smaller enrichment. However, as food isolates were derived from different provinces, a direct within-province comparison between food workers and food isolates remains unavailable. As noted in our response to Reviewer 1, Q1, province was included as a random effect in all mixed-effects models as partial mitigation, but residual geographic confounding cannot be excluded given the non-overlapping sampling frames.

We have made this explicit in the revised manuscript, and the food worker enrichment is now presented as a robust epidemiological association supported by multiple analytical approaches, while cautioning against causal interpretation. We have updated the Results Lines 169-222, and Methods Lines 954964, and below. Also, please note when re-running the analysis, we identified a transcription error in the original manuscript: the proportions of high-risk traits reported in the text (86% in food workers, 45% in food isolates) did not match the correct underlying values from which the reported test statistic (*p*-value = 4.289×10^{-21}) was derived. The correct values are 95% (food workers) and 59% (food isolates); the statistical test itself was performed correctly on the accurate data, and only the prose-reported percentages have been corrected.

Results Lines 169-222: Notably, 95% of all high-risk traits (Rank IV and V) occurred in food worker isolates, a significantly higher proportion than in food isolates (retail pork and chicken meat) (59%; *p*-value = 4.289×10^{-21}, proportion test, two-tailed). As differences in sample size across source categories can affect the likelihood of observing rarer traits, we checked whether this different was due only to more rare traits being found in the larger group. Firstly, assessing the proportion of high-risk AMR traits exclusively found in food workers vs those found in both food workers and food. Overall, only 35.5% of traits were found in food workers alone, compared to 64.5% that we also found in food and/or livestock, indicating that most high-risk traits were shared across food and/or livestock. Secondly, a bootstrapping analysis (10,000 iterations) in which food worker isolates were down-sampled to match the sample size of food isolates. This confirmed a smaller but statistically significant enrichment of high-risk traits in food worker isolates compared to food isolates after sample size correction (mean difference 7%; 95% CI [3.4%, 11.3%], one-tailed bootstrap *p*-value= 0.0004), indicating that while the magnitude of the difference was is part inflated by sample size, the direction and significance of the enrichment reflect a true biological difference between groups.

To assess whether this enrichment might reflect a systematic bias in the risk assessment framework toward human-derived isolates more broadly, or broader human-associated AMR patterns rather than food worker-specific characteristics, we applied the same bootstrapped down-sampling analysis to the two other human source groups in our dataset. Non-food worker isolates, recruited from the same Guangxi Province under the same occupational health surveillance programme as food workers, providing the strongest available control for both human-source effects and regional epidemiological differences, showed a slightly higher proportion of high-risk traits than food isolates after sample size correction (mean difference 4.3%; 95% CI [1.3%, 7.1%], one-tailed bootstrap *p*-value=0.002), but with a smaller effect size than food workers. Symptomatic patient isolates showed a significantly lower proportion of high-risk traits than food isolates after sample size correction (mean difference -5.9%; 95% CI [-9.2%, -2.5%], one-tailed bootstrap *p*-value = 0.0002). We note that the symptomatic patient comparison is weaker evidence than the non-food worker comparison, as symptomatic patients differ from food workers in province, collection period, and health/exposure status simultaneously, whereas non-food workers differ only in occupational food contact within an otherwise matched sampling frame. As a further test, food worker isolates were directly compared to non-food worker isolates (all collected as part of the same surveillance programme) removing confounding factors of host, geography, collection and years. This showed that after down-sampling, although not statistically significant, food workers had a slightly higher proportion of high-risk AMR traits compared to non-food workers (mean difference 2.35%, CI [-1.68%, 6.3%], one-tailed bootstrap *p*-value=0.31).

The gradient of effect sizes across human source groups, food workers 7% > non-food workers 4.3% > symptomatic patients -5.9%, is inconsistent with a uniform artificial inflation of risk scores across

human sources and instead supports a genuine occupational exposure gradient specific to food-related work. Nevertheless, as food workers and food isolates were derived from different provinces, residual geographic confounding cannot be fully excluded (see limitations in the Discussion), and the enrichment should be interpreted as an epidemiological association rather than a causally attributed source effect.

As an additional assessment the decomposed risk score was also analysed, specifically considering the risk severity only and disregarding the risk likelihood that contained the mobility component, which was host dependent. Considering AMR traits with a risk severity score of 4 or 5, (which included only Rank I clinically significant traits and MDR traits conferring resistance to antibiotics used in both animals and humans and on the AWARE Watch or Reserve lists), revealed similar results to the previous analysis. Specifically, food worker isolates vs food isolates: mean difference +7.1% (95% CI [2.8%, 11.3%], one-tailed bootstrap *p*-value=0.0006); non-food worker isolates vs food isolates: mean difference +5.0% (95% CI [2.43%, 7.7%], one-tailed bootstrap *p*-value=0.0002); Symptomatic patient isolates vs food isolates: mean difference -5.9% (95% CI [-8.9%, -2.8%], one-tailed bootstrap *p*-value=0.0001).

Methods Lines 954-964: *Analysis of proportions of high-risk traits by source type* For each human source type-food pair, bootstrap resampling with 10,000 iterations was used to down-sample the larger group to obtain equal sample sizes. At each iteration, the difference in proportion of AMR traits observed in the now equally sized groups was calculated to provide an empirical distribution of differences in the proportion of AMR traits carried between the human source group and pooled food group. Mean difference and 95% confidence intervals were estimated. An empirical *p*-value was calculated as the proportion of bootstrap replicates showing in which the difference in proportions was less than or equal to 0 (given a positive observed effect), consistent with a null hypothesis of no difference. A secondary analysis was conducted using an identical methodology but using the Risk severity score only. Risk severity scores of 4 or 5 were included.

3.The forecasting framework appears to be based primarily on pairwise associations between individual indicators and AMR trait prevalence. While this approach is transparent and easy to interpret, many of the examined indicators (e.g. healthcare expenditure, urbanisation, population growth, and antimicrobial consumption) are likely to exhibit strong correlations with one another and may reflect broader underlying socioeconomic trends. As a result, some reported associations may be difficult to disentangle and could reflect shared temporal patterns rather than direct relationships with AMR trait prevalence.

We thank the reviewer for this relevant methodological observation, which we have addressed through a combination of existing analytical safeguards, new analyses, and explicit acknowledgement of inherent limitations.

Existing safeguards against spurious temporal associations

Our pipeline already incorporated several steps specifically designed to mitigate associations driven purely by shared temporal trends rather than genuine relationships with AMR trait prevalence. First, each indicator-prevalence pair was subjected to ADF stationarity testing and Johansen cointegration testing. For pairs where both series were non-stationary and not cointegrated, first-order differencing was applied before correlation calculation. Critically, first-order differencing removes shared linear trends from both time series prior to correlation, meaning that associations retained after this step reflect co-movement in year-on-year changes rather than shared upward trajectories. For cointegrated pairs, original levels were retained as the shared trend in this case reflects a genuine long-run relationship rather than spurious co-trending. Second, permutation-based significance testing with Benjamini-

Hochberg FDR correction (FDR threshold 10%) was applied to guard against false positives arising from large-scale pairwise testing. Together, these steps substantially reduce but do not eliminate the possibility that some retained associations reflect shared temporal patterns.

New analysis 1: Indicator-indicator correlation structure

To directly assess multicollinearity among the 96 indicators, we calculated pairwise Pearson correlations between all indicator pairs following the same time-series preprocessing framework described above, using Engle-Granger cointegration testing for pairwise assessment (in contrast to the Johansen test used in the main pipeline, which is designed for multivariate settings; Engle-Granger is the appropriate choice for pairwise cointegration assessment). This showed that indicator-indicator associations are on average weak (mean $|r| = 0.237$; range: -0.999-1 with most indicator pairs showing low correlation (Figure S31). Strong correlations ($|r| > 0.5$) were limited and generally confined to indicators within the same category. The two largest correlated clusters comprised antibiotic usage indicators and socioeconomic poverty gap indicators respectively, which is expected given their shared measurement domain. Importantly, cross-category correlations were generally weak, supporting the appropriateness of treating indicators from different categories as largely independent in the pairwise modelling framework.

New analysis 2: Indicator category breadth of AMR trait associations

To further characterise whether AMR trait associations were associated with a single shared indicator cluster or reflected genuine associations across multiple independent indicator groups and clusters, we examined the distribution of indicator categories associated with each forecastable AMR trait (Supplementary Data 13 (was Supplementary Data 9)). 78.2% of forecastable traits were associated with indicators from two or more distinct categories, suggesting that their projected trajectories are supported by convergent evidence across independent indicator domains rather than a single shared trend. Similarly, 81.2% of forecastable traits were associated to two or more indicator clusters (based on the clustering above). This cross-category convergence strengthens confidence in the specificity of the gene-level associations reported.

New analysis 3: Sensitivity analysis using representative indicators per cluster

To assess whether the results were materially influenced by redundancy within correlated indicator clusters, we repeated the forecasting analysis using one representative indicator per identified cluster, specifically the indicator showing the highest absolute Pearson correlation with AMR trait prevalence within each cluster. The set of traits identified as forecastable and the direction of their projected trajectories were broadly consistent with those reported in the main analysis, with 95% of traits retained, confirming that the results are robust to indicator redundancy within correlated clusters, with only a small number of differences observed. The small proportion of traits (5%) reflect cases where the forecasting association was linked to specific indicators. Collapsing these indicators into a single representative indicator can cause the trait-indicator pair to fall below the applied regression and FDR thresholds in the regression analysis, hence traits only associated to these indicators were lost. For example, *qnrB5* was associated to the indicator J01A: tetracyclines. This indicator however was replaced with the cluster representative indicator "*Labor force participation rate*", which due to low R^2, did not have a fitted temporal trend, hence was filtered out in the regression analysis. Under stricter clustering of $|r| \geq 0.8$, such that only very strongly correlated indicators are clustered, 100% of traits would have been retained.

New analysis 4: PCA of indicator space

To characterise the broader latent structure of indicator co-variation and assess whether AMR traits map onto distinct underlying drivers, we conducted a principal component analysis (PCA) across the 96 indicators following time-series preprocessing. The first two principal components together explained

only 31.97% of total indicator variance, and eight components were required to explain over 80% of variance, indicating that indicator co-variation is not dominated by a small number of latent factors. PC1 reflected broad economic development (loading strongly on poverty indicators, healthcare expenditure, and urbanisation) while PC2 was dominated by antibiotic consumption patterns with a small number of poverty indicators, other components were mixed. AMR traits were associated with indicators distributed across PCA components, supporting the gene-specific interpretation of the associations and demonstrating that different resistance determinants are driven by different underlying socioeconomic dimensions rather than a single shared trend. Specifically, across all AMR traits, the average top PC fraction (proportion of the total trait-associated absolute PCA loading score attributable to a single PCA component) was only 10.6%, which was not greater than would have been expected by chance (tested with a random trait-label permutation test with 5000 permutations, *p*-value = 0.999). Similarly, the mean top-versus-second PC margin was also small (0.0126; *p*-value = 0.990). These high *p*-values indicate that the observed trait-PC specificity was no greater than expected under random trait-label assignment, consistent with AMR traits being associated with multiple independent components rather than concentrated on a single dominant axis of variation.

Inherent limitation: correlational nature of associations

We acknowledge that despite all of the above precautions, all indicator-trait associations remain correlational and cannot establish causality or directionality. Indicators reflecting broad economic development trajectories, such as healthcare expenditure, urbanisation, and population growth, may in part act as proxies for a shared underlying driver that cannot be fully decomposed through pairwise analyses given the constraints of national-level time series data with a maximum of 17 years of overlap. This is an inherent limitation of ecological surveillance studies at the national level and is not resolvable with the available data. The associations reported here should therefore be interpreted as identifying candidate structural drivers of AMR trait prevalence that warrant further investigation through prospective, regionally stratified, and ideally interventional study designs, rather than as evidence of direct causal relationships. We have added an explicit statement of this limitation to the revised manuscript.

We have edited the manuscript to include Supplementary Figure S31, and these additional analyses and discussion of limitations in the Methods Lines 1194-1232, Results Lines 366-395 and Discussion Lines 627-649 and below.

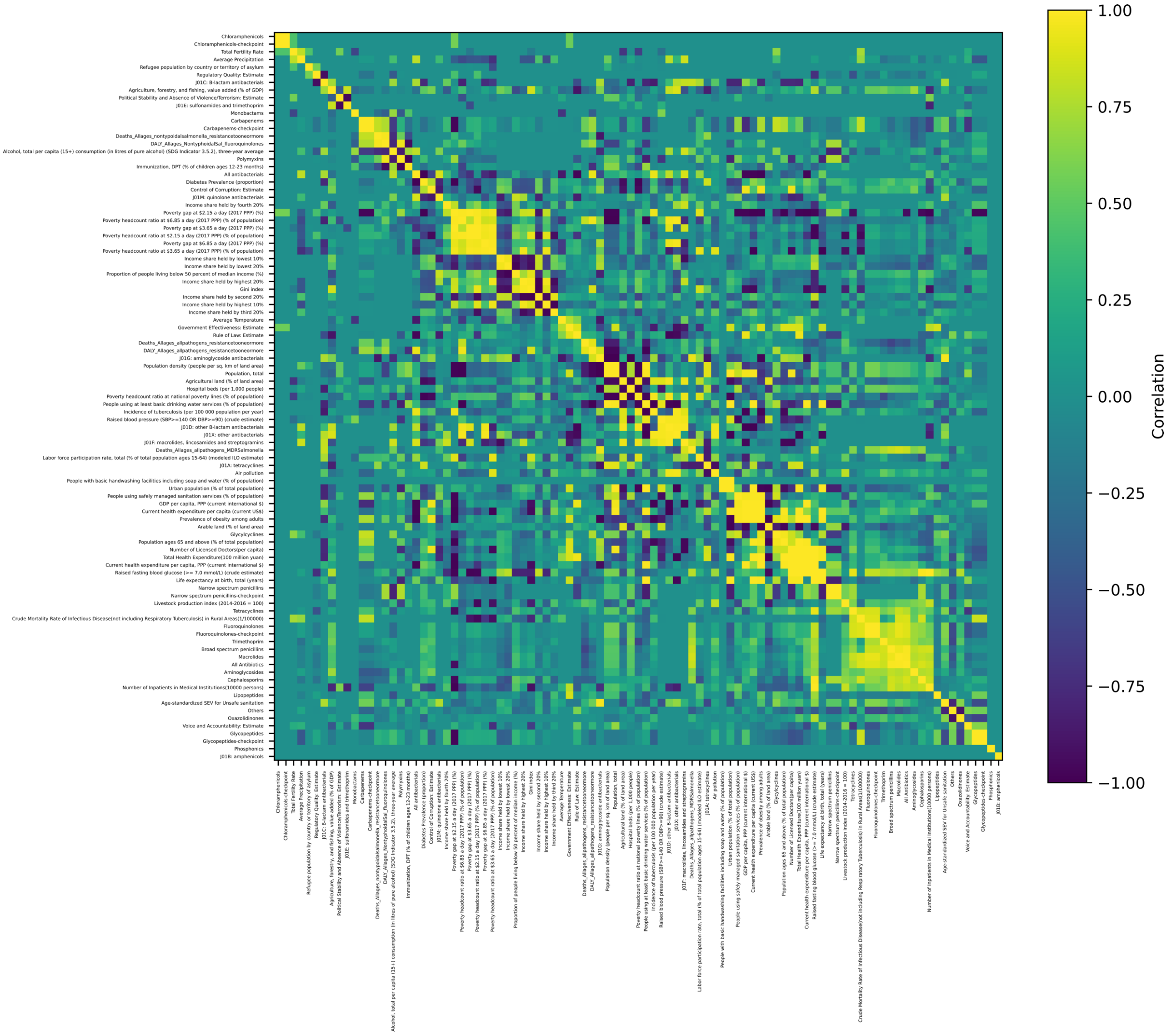

Supplementary Figure S31: **Indicator-indicator correlation.** Clustered heatmap showing the amount of correlation between the indicators used in this study. Colours of the heatmap indicator the Pearson *r* value of each pair of indicators. Indicators were clustered based on |r| include positive and negative associations in the clustering.

Methods Line 1194-1232:

Indicator-indicator correlation analysis

To assess the degree of multicollinearity among the 96 indicators used in the forecasting pipeline, pairwise Pearson correlations were calculated between all indicator pairs in Python SciPy v1.15.2 To address autocorrelation, each indicator pair was first subjected to ADF stationarity testing and Engle-Granger cointegration testing. Pairs where both indicators were non-stationary and not cointegrated (*p*-value ≥ 0.05) were first-order differenced prior to correlation calculation; otherwise original data were retained. Pairwise correlations were visualised as a clustered heatmap with hierarchical clustering based on absolute correlation coefficients |r|, to identify clusters of co-varying indicators regardless of the direction of association. Indicators were also grouped into indicator clusters based on the strength of their correlation (|r| > 0.5).

Indicator category breadth analysis

For each forecastable AMR trait, the number of distinct indicator categories (socioeconomic, health-related, antibiotic consumption, climate, governance) represented among its significantly associated indicators was calculated from Supplementary Data 13. Similarly for each forecastable AMR trait, the

number of distinct indicator clusters was calculated. Traits were classified as associated with a single indicator category/cluster or with multiple indicator categories/clusters to assess whether projected trajectories were supported by convergent evidence across independent indicator domains.

Sensitivity analysis: representative indicators per cluster
For each correlated indicator cluster identified in the indicator-indicator correlation analysis, a single representative indicator was selected based on the highest absolute Pearson correlation with AMR trait prevalence. The full forecasting pipeline was then re-assessed using only these representative indicators, and the resulting set of forecastable traits and their projected directions were compared with those from the main analysis to assess robustness to indicator redundancy.

Principal component analysis of indicator space
To characterise the latent structure of indicator co-variation, principal component analysis (PCA) was conducted across the 96 indicators using time-series preprocessing described above (stationarity testing and first-order differencing where appropriate). The number of components retained was determined by cumulative variance. Component loadings were examined to characterise the underlying socioeconomic, health, and environmental dimensions represented by each component. The association between forecastable AMR-trait indicator pairs and principal component loadings was assessed by mapping the AMR-trait associated indicators onto the PCA loading structure, to calculate the fraction of each PCA component associated to each trait. Permutation testing was used to randomly permute AMR trait labels 5000 times, to generate a null distribution, testing whether the observed trait-component profiles showed greater specificity to any particular principal component than would be expected by chance.

Results Line 366-396:
Indicator-indicator correlation and robustness analyses

To assess multicollinearity among the 96 indicators used in the forecasting pipeline, pairwise Pearson correlations were calculated between all indicator pairs following time-series preprocessing (see Methods). Indicator-indicator associations were on average weak (mean $|r| = 0.237$; range: (-0.999 to 1), with the majority of indicator pairs showing low correlation (**Fig. S31**). Strong associations ($|r| > 0.5$) were limited and generally confined to indicators within the same category, with the two largest correlated clusters comprising antibiotic usage indicators and socioeconomic poverty gap indicators respectively. Cross-category correlations were generally weak, supporting the independence of associations across indicator domains.

Principal component analysis identified two major components explaining 31.97% of total indicator variance, with 8 components needed to explain 80%. Principal component (PC) 1 reflected broad economic development (loading strongly on poverty indicators, healthcare expenditure, and urbanisation) while PC2 was dominated by antibiotic consumption patterns with a small number of poverty indicators, other components were mixed. The average top PC fraction (proportion of the total trait-associated absolute PCA loading score attributable to a single PCA component) was only 10.6%, which was not larger than would have been expected by chance (one-tailed permutation test, *p*-value = 0.999, see Methods). Similarly, the mean top-versus-second PC margin was also small (0.0126; one-tailed permutation test, *p*-value = 0.990). These high *p*-values indicate that the observed trait-PC specificity was no greater than expected under random trait-label assignment, consistent with AMR traits being associated with multiple independent components rather than concentrated on a single dominant axis of variation, supporting the gene-specific interpretation of the indicator associations.

A sensitivity analysis repeating the forecasting pipeline with one representative indicator per correlated cluster confirmed that the set of forecastable traits and the direction of their projected trajectories were consistent with the main analysis (95% of AMR traits retained), demonstrating robustness to indicator redundancy within correlated clusters.

Examination of indicator category breadth showed that 78.2% of forecastable AMR traits were associated with indicators from two or more distinct indicator groups and 81.2% were associated with two or more distinct indicator clusters, indicating that their projected trajectories are supported by convergent evidence across independent indicator domains.

Discussion Lines 627-649:
A further limitation concerns the correlational nature of all indicator-trait associations reported in the forecasting analysis. Despite the application of stationarity testing, cointegration testing, first-order differencing, and permutation-based FDR correction to mitigate spurious associations as a result of shared temporal trends, the pairwise framework cannot establish causality or directionality between individual indicators and AMR trait prevalence. Some indicators reflecting broad economic development trajectories, including healthcare expenditure, urbanisation, and population growth may act in part as proxies for a shared underlying driver that cannot be fully decomposed through pairwise analyses given the constraints of national-level time series data and the limited number of annual observations available (maximum 17 years of overlap). This is an inherent limitation of ecological surveillance studies at the national level. The indicator-trait associations reported here should therefore be interpreted as identifying candidate structural factors associated with AMR trait prevalence warranting further investigation through prospective, regionally stratified study designs, rather than as evidence of direct causal relationships. Future studies incorporating subnational data, longitudinal cohort designs, or natural experiment frameworks, such as policy changes affecting antibiotic use, would enable stronger causal inference about the structural determinants of AMR spread across China's food chain.

Reviewer #2 (Remarks to the Author)

This manuscript describes a large genomic study of antimicrobial resistance determinants in Salmonella enterica from multiple sources across the food chain in China, which was leveraged along with socioeconomic, environmental, and health indicator data to develop a predictive model for AMR traits that projects the prevalence of these traits to 2050. The methods are meticulously described and the bioinformatic and modeling parameters chosen are well thought-out and adequately defended and evaluated. The results provide a comprehensive assessment of the current AMR landscape in S. enterica in China, and a detailed forecast of AMR traits in this species in relation to demographic and environmental indicators.

Please see below for specific comments and questions related to this manuscript:

1. The authors might consider re-wording the title to avoid using the acronyms AMR and MDR prior to their definition.

We thank the reviewer for this comment, we have removed the abbreviations from the title, also shortening the title length to meet the journal requirements. The revised title is:

High-risk antimicrobial resistance genomic traits in multidrug-resistant *Salmonella* across China's food chain: projections to 2050

2. Is it known what occupations the non-food workers sampled held? Were all of them at low risk of occupational exposure to Salmonella?

We thank the reviewer for raising this important point. Classification of individuals as food workers or non-food workers was based on the definitions established under China's Food Safety Law (2015), Article 45, which legally distinguishes workers who enter into contact with food for direct consumption from those whose roles involve no such contact (Food Safety Law of the People's Republic of China

(2015), Article 45; English translation and summary available via USDA Foreign Agricultural Service, GAIN Report CH15016). The Food Safety Law, mandates annual health examinations and a valid health certificate for food workers. Non-food workers were defined as individuals working in sectors such as leisure, entertainment, beauty and cosmetics services, and food retail settings *without direct food contact*. Both groups were drawn from the same occupational health screening programme and therefore underwent equivalent health surveillance, differing specifically in their occupational exposure to food.

We have information on the occupations of the all-sampled non-food worker individuals., but in some cases this would be unreliable due to inaccurate free-text self-reported job descriptions recorded at the point of occupational health screening. To avoid this source of error, classification was based on the rigorous definition, which we applied, based on the recorded presence or absence of a food handler health certificate, consistent with the legal definition under Article 45, which we consider the more reliable classification criterion. This approach was confirmed with our collaborators at the Chinese Center for Disease Control and Prevention, who advised that certificate status, rather than job title, is the operative criterion under Chinese regulation on whether there is contact or not with food. We have added a definition of food worker and non-food worker classification to the Methods.

The list of occupation of the non-food workers is given in the Supplementary Data 1.

Regarding occupational risk of *Salmonella* exposure specifically: by definition, non-food workers had no direct contact with unprocessed or ready-to-eat food, which is the principal transmission route for *Salmonella* in occupational settings, and therefore represent a substantially lower-exposure occupational group than food workers. We cannot, however, exclude the possibility of incidental or non-occupational exposure to *Salmonella* among non-food workers (e.g., through their own food handling or consumption outside the workplace), as is the case for any member of the general population, and our classification reflects relative occupational risk rather than absolute risk of zero exposure. This is consistent with our use of non-food workers as a lower-exposure comparator group rather than a zero-risk control group, and we have clarified this distinction in the Methods Lines 741-747.

Methods Line 741-747: Food worker and non-food worker classification followed the definitions established under Article 45 China's Food Safety Law (2015), which distinguishes workers based on whether they enter in contact with food destined to direct consumption (Food Safety Law of the People's Republic of China (2015), Article 45; English translation and summary available via USDA Foreign Agricultural Service, GAIN Report CH15016). Classification was based on the recorded presence or absence of a food handler health certificate. Both groups were drawn from the same occupational health screening programme and therefore underwent equivalent health surveillance, differing specifically in their occupational exposure to food.

3. In the bioinformatic section of the methods (starting on Line 156), it is unclear which methods in the analysis were applied only to the newly sequenced isolates from this study and which were applied to the previously sequenced publicly available sequences.

We thank the reviewer for identifying this lack of clarity and have now revised the Methods to explicitly distinguish between analyses applied only to the newly sequenced isolates and those applied to all isolates. The revised text is provided below and on Lines 798-835 of the revised manuscript

Methods Lines 798-835:
DNA Extraction
Genomic DNA was extracted from 279 MDR and 138 non-MDR Salmonella isolates using the Wizard Genomic DNA Purification kit (Promega, WI, USA). DNA concentrations were quantified with the Qubit dsDNA HS Assay kit (Thermo Fisher Scientific, MA, USA). Libraries were constructed using

the MGIEasy FS DNA Library Prep Set (MGI, China) and were paired-end sequenced on the MGISEQ-200RS and MGISEQ-2000RS platforms.

Genome assembly and annotation

For newly sequenced data: Raw reads were processed using fastpv1.0.0 (parameters -q 30 -l 50), which filtered low-quality bases ($Q < 30$) and retained only reads longer than 50 bp in length. Genomes were assembled using Spades v3.15.5 with the parameters '--careful --cov-cutoff auto'.

Assembly quality was assessed using QUAST v5.0.2 (with default parameters) and genome completeness and contamination were assessed using CheckMv1.2.2 (with default parameters) with the lineage_wf pipeline. Contigs shorter than 500 nucleotides were excluded from the final assemblies. For newly sequenced genomes, assemblies were retained if they met the following quality thresholds: N50 ⩾ 15kb, genome completeness ⩾ 80% and contamination ⩽ 5%; assemblies failing these criteria were excluded from further analysis.

For publicly available assemblies: Sequences associated to Bioproject PRJNA1082900 were obtained as pre-assembled genome sequences (assembled using Spades v3.15.5) from the National Genomics Data Center. These were also assessed against the same completeness and contamination thresholds described above prior to inclusion; assemblies failing these criteria were excluded.

For all other sequenced raw reads were downloaded from NCBI and the same pipeline used for the newly sequenced data was applied. The same completeness and contamination thresholds were applied to all publicly available sequences; assemblies failing these criteria were excluded from further analysis.

Both publicly available and newly sequenced data were then aggregated, and genomes were annotated with Bakta v1.4.0, with '--genus Salmonella --force --compliant --skip-crispr'.

Sequence types were identified using MLSTv2.23.0 (with --scheme senterica_achtman_2), and serotypes and serogroups were identified *in silico* using SISTR v1.0.2 with default settings.

4. The annotation of assemblies with Abricate for ARG and IS identification is described 3 times in the Methods section; the authors might consider removing this redundancy.

Correctly the Reviewer found that Abricate-based screening against the CARD and ISfinder databases was described in three separate locations in the original Methods (original manuscript lines 163-165; Lines 169-174; and within the Mobile ARG cassettes identification section, original manuscript lines 200-203). We have now consolidated this methodology into a single, complete description within the "Screening of annotated genes against ARG and MGE databases" section, and replaced the other two instances with brief cross-references to this section, removing duplicated parameter descriptions while preserving full methodological detail in one place. The revised text is provided below.

Methods Line 837-846 (consolidated section):

Screening of annotated genes against ARG and MGE databases

Genome assemblies were screened against the CARD database (accessed 01-08-2024) using Abricate v1.0.1 at a stringent threshold of ⩾ 95% coverage and ⩾ 95% identity (parameters –db card –mincov 95 –minid 95) to identify known AMR-associated genes across the isolate cohort; these thresholds were chosen to minimise false positives and variant uncertainty (Schmidt et al. J Antimicrob Chemother 72, 104-114, 2017, DOI: 10.1093/jac/dkw397). AMR-associated genes were then manually curated and ten genes (*BRP(MBL)*, *golS*, *mdsA*, *mdsB*, *mdsC*, *mdtK*, *mrx*, *qacEdelta1*, *sdiA* and *qacL*) that do not confer AMR in S. enterica were removed. Genome assemblies were also screened against the ISfinder database (accessed 01-08-2024) using Abricate v1.0.1 at the same thresholds (⩾95% coverage and ⩾95% identity) to identify insertion sequences across the isolate cohort.

Methods Lines 871-878: (Mobile ARG cassettes identification section, revised)

Original text: Filtered contigs (> 500bp) were searched for ARGs and insertion sequences using Abricate against CARD and ISfinder databases at high identity (95%) and coverage (95%) thresholds to minimise false positives and variant uncertainty.

Revised text:To identify mobile ARG content across source types, the distance between each identified ARG and MGEs were calculated from their positions (see above in '*Screening of annotated genes against ARG and MGE databases and in silico subtypin*g').

5. How were antimicrobial susceptibility data from previously sequenced publicly available isolates retrieved? Did the interpretations all use the same M100 30th Edition breakpoints as the new isolates from this study?

For the publicly available isolates, AST data were taken directly from the original published sources, which used equivalent CLSI breakpoints; this is now stated explicitly in the revised Methods Lines 787-796. Specifically, M100 CLSI breakpoints from the 27th, 28th 30th or 31st editions were used (depending on the study), which all contained the same breakpoints for the isolates tested.

Methods Lines 787-796: For publicly available isolates, AST data were taken directly from the original published sources. We reviewed the methods of each source publication to confirm the CLSI M100 edition used for breakpoint interpretation (27th, 28th, 30th, or 31st edition, depending on publication date) and cross-checked the relevant breakpoints for each antibiotic tested with our models across these editions (Table S2). Breakpoints for all antibiotics included in our analysis were unchanged across these editions for *Salmonella* spp., confirming interpretive consistency between the publicly available and newly generated AST data. For streptomycin and moxifloxacin no breakpoints are given in any of the CLSI M100 editions, so we relied on the RSI interpretations given in the original source.

6. Given the overlap of MDR and non-MDR isolates in the maximum likelihood phylogenetic tree, why were separate PopPUNK models used for MDR and non-MDR isolates?

We thank the reviewer for this question. Although the maximum likelihood phylogeny shows a high degree of intermixing between MDR and non-MDR isolates at the core genome level, we used separate PopPUNK models for the following reasons.

First, PopPUNK clusters were used as random effects in the GLMM framework and therefore needed to accurately reflect the population structure of the specific dataset being modelled, rather than a shared population structure derived from both datasets combined. We compared clustering under separate versus joint PopPUNK model and found that combining non-MDR isolates with the MDR dataset substantially reduced the number of resolved clusters in the MDR data (from 141 to 83) and to a smaller extent in the non-MDR data (from 31 to 29), with previously distinct clusters merging together. This reduction reflects genuine lineages being collapsed into coarser groupings under the joint model, rather than a simplification with no analytical cost: since PopPUNK cluster membership is used directly as a random effect to control for population structure in our GLMM framework, this loss of resolution would reduce the model's ability to correctly attribute variance to fine-scale lineage effects. Additionally, under a joint model, cluster definitions would be dominated by much larger MDR datasets (n=2,536 isolates), with non-MDR isolates (n=306) assigned to clusters defined primarily by MDR population structure rather than having their own lineage structure independently resolved. As the change in clustering was significant under merged clustering model, we did not run the GLMM under this constraint and retained the separated clustering approach.

Second, PopPUNK clustering is based on both core and accessory genome distances, not solely the core genome used to construct the maximum likelihood phylogeny. The accessory genome can capture fine-scale population structure and recent evolutionary divergence not visible in core genome-based phylogenies, which may be particularly relevant for AMR-associated mobile genetic elements, and this structure may differ meaningfully between MDR and non-MDR datasets independently of their core genome relationships. The intermixing observed in the core-genome phylogeny therefore does not

preclude meaningful independent population structure at the accessory genome level captured separately by each PopPUNK model.

We have added a brief justification of this choice to the Methods section of the revised manuscript.

Methods Lines 1015-1024:
After comparing separate and joint (combining MDR and non-MDR) PopPUNK models, separate PopPUNK models were constructed for the MDR and non-MDR datasets. Combining datasets substantially reduced cluster resolution (MDR: 141 to 83 clusters; non-MDR: 31 to 29 clusters), with genuine lineages collapsing into coarser groupings, and resulted in cluster definitions being dominated by the larger MDR dataset (n=2,536) relative to the non-MDR dataset (n=306). As PopPUNK cluster membership was used as a random effect in all GLMM models to control for population structure, separate models were retained to preserve fine-scale lineage resolution independently for each dataset. PopPUNK clustering incorporates accessory genome distances in addition to core genome distances, capturing structure not visible in the core-genome phylogeny used for visualisation.

7. In line 543, it is stated that a higher proportion of high-risk traits occurred in food worker vs. food isolates. However, AMR traits present only in human sources were by definition assigned a higher inter-host mobility score, and therefore a higher risk score, than traits present only in food sources, so this may be in part an artificially imposed association.

We thank the reviewer for this important methodological observation. We agree that the inter-host mobility component of the risk assessment framework creates a potential circular relationship between source type and risk score: traits found exclusively in human sources receive higher mobility scores by definition, which could in principle artificially inflate the proportion of high-risk traits observed in food workers relative to food isolates. We note first that inter-host mobility is a genuine biological property of AMR traits: traits found across human, food, and livestock sources have demonstrated capacity for cross-host spread, and their higher risk scores reflect this epidemiological reality. Nevertheless, we agree that the structural relationship between source type assignment and mobility scoring warrants explicit testing, and we have now done so.

To test this directly, we decomposed the risk score and analysed the risk severity component alone, disregarding the risk likelihood score, which contains the host-dependent mobility component that is the specific focus of the reviewer's concern. Considering only AMR traits with a risk severity score of 4 or 5 (comprising Rank I clinically significant traits and MDR traits conferring resistance to antibiotics used in both animals and humans and on the AWARE Watch or Reserve lists), we repeated the bootstrapped down-sampling comparison (10,000 iterations) between food workers, non-food workers, symptomatic patients, and food isolates. This severity-only score removes the mobility component entirely, so any enrichment observed cannot be attributed to the inter-host mobility scoring criterion the reviewer has identified. The results were as follows: food worker isolates vs. food isolates, mean difference +7.1% (95% CI [2.8%, 11.3%], one-tailed bootstrap *p*-value = 0.0006); non-food worker isolates vs. food isolates, mean difference +5.0% (95% CI [2.43%, 7.7%], one-tailed bootstrap *p*-value = 0.0002); symptomatic patient isolates vs. food isolates, mean difference −5.9% (95% CI [−8.9%, −2.8%], one-tailed bootstrap *p*-value = 0.0001). These results closely mirror those obtained using the full risk score, indicating that the food worker enrichment persists even when the mobility component is excluded entirely, and is therefore not attributable to the inter-host mobility scoring criterion alone.

To further test whether the risk assessment framework systematically inflates risk scores for human-derived isolates more broadly, we used the two other human source groups in our dataset, non-food workers and symptomatic patients, as internal controls (see also our response to Reviewer 1, Question 2, where this analysis is described in full). We reasoned that if the risk assessment framework artificially biases toward higher risk scores in human isolates as a class, all three human source groups should show

a similarly elevated proportion of high-risk traits relative to food isolates. Using the full risk score and the same bootstrapped down-sampling approach, the results were: food worker isolates vs. food isolates, mean difference +7% (95% CI [3.4%, 11.3%], one-tailed bootstrap *p*-value = 0.0004); non-food worker isolates vs. food isolates, mean difference +4.3% (95% CI [1.3%, 7.1%], one-tailed bootstrap *p*-value = 0.002); symptomatic patient isolates vs. food isolates, mean difference −5.9% (95% CI [−9.2%, −2.5%], one-tailed bootstrap *p*-value = 0.0001); and food worker isolates vs. non-food worker isolates, mean difference +2.35% (95% CI [−1.68%, 6.3%], one-tailed bootstrap *p*-value = 0.31, non-significant). The gradient of effect sizes across human source groups, food workers (+7%) > non-food workers (+4.3%) > symptomatic patients (−5.9%), is inconsistent with a uniform artificial inflation of risk scores applied equally across all human sources, though it does not rule out a smaller, partial contribution from the mobility scoring component, which we have already addressed directly above using the severity-only decomposition. A systematic scoring bias affecting all human-derived isolates equally would be expected to produce similar enrichment across all three groups, since all three receive the same higher inter-host mobility score relative to food-only traits under the scoring logic. Instead, the divergent direction of the symptomatic patient result and the graded pattern across groups support the interpretation that the food worker enrichment reflects a genuine biological signal, specifically an occupational exposure gradient, rather than a scoring artefact.

We also assessed the proportion of high-risk AMR traits found exclusively in food workers versus those shared with food and/or livestock sources. Overall, only 35.5% of high-risk traits were found in food workers alone, while 64.5% were also found in food and/or livestock sources, indicating that the great majority of high-risk traits identified in food workers are not unique to that source but are shared across the food chain.

We acknowledge that the non-food worker result, which also shows a modest but significant enrichment relative to food isolates under both the full and severity-only scores, could be interpreted as partial evidence of a residual human-source effect in the risk scoring. We therefore do not claim to have entirely excluded a partial contribution of the mobility scoring criterion to the observed enrichment, but conclude, on the basis of the severity-only decomposition and the graded pattern across human source groups, that the scoring framework is not the primary explanation for the food worker-specific pattern.

We have updated the Results section accordingly and have added an explicit note on the interpretation of the risk scoring framework in the context of this analysis to the Discussion/Limitations section. The full revised Results text reporting these bootstrapped comparisons across all three human source groups is provided in our response to Reviewer 1, Questions 1 and 2 (Results Lines 169-222, Methods Lines 954-964, and Discussion Lines 580-649) and is not duplicated here to avoid repetition; we refer the editor and reviewers to that response for the complete text as it appears in the revised manuscript.

8. There are only single values for the socioeconomic, environmental, and health indicator data used to represent all of China – do the authors have insight into how much these indicators vary geographically within the country, and whether this might affect the results of the study?

We thank the reviewer for this important and insightful question. The use of national-level aggregate values for socioeconomic, environmental, and health indicators is a genuine limitation of our study, and one we wished to address as concretely as possible in our response.

China exhibits substantial and well-documented geographic heterogeneity across all the indicator categories used in our analysis. According to the National Bureau of Statistics of China (NBS) (National Bureau of Statistics of China. China Statistical Yearbook. Beijing: NBS; 2022), per capita GDP across Chinese provinces in our dataset varies more than fourfold, from approximately 206,826 yuan in Beijing to approximately 52,399 yuan/person in Guangxi in 2022, with the national average at approximately

87,385 yuan/person in 2022. This variation is directly relevant to our dataset, with substantial heterogeneity observed between provinces (average coefficient of variation per year across provinces 0.5, range 0.45-0.64). Importantly, Guangxi, from which the majority of food worker isolates in our study were collected (n=1,972 MDR isolates), consistently had one of the lowest per capita GDP of approximately across all years our data covered (Supplementary Data 11, approximately 40% (range 38% - 44%) below the national GDP (National Bureau of Statistics of China. China Statistical Yearbook. Beijing: NBS; 2022), and roughly one-quarter that of Beijing or Shanghai. Guangxi is therefore one of the economically least developed provinces in our dataset, in stark contrast to more economically developed provinces such as Shandong and Guangdong, which are also represented. Province-level variation in health expenditure efficiency and healthcare workforce density is similarly substantial: a study drawing on NBS and China Health Statistical Yearbook data documented nearly threefold differences in health workers per thousand citizens between the best and least served provinces across China (Beijing: 9.48 per thousand; lowest recorded: 3.58 per thousand), with the efficiency gap in health expenditure particularly pronounced in eastern provinces (Shi Y, *et al. Frontiers in Public Health.* 2022;10:879698. doi:10.3389/fpubh.2022.879698). Within-province variation in antibiotic consumption has also been documented: a panel data analysis of Shandong province showed substantially different consumption patterns between high-income and upper-middle-income areas within the same province, with watch antibiotics dominating consumption in high-income areas while access antibiotics predominated in upper-middle-income areas, demonstrating that national-level antibiotic consumption figures mask considerable subnational heterogeneity (Yin J et al. Front Pharmacol. 2021 Nov 22;12:790817. doi: 10.3389/fphar.2021.790817).

This heterogeneity is directly relevant to our study and we acknowledge it as a genuine limitation. However, our analytical framework addresses it in two important ways. First, province is explicitly included as a categorical random effect in all GLMM models. The province interclass correlation coefficient (ICC = 0.149) was among the largest of the four random effects, indicating that the models captured a substantial proportion of province-level variance in AMR trait prevalence even without province-specific indicator time series. This means that province-level differences in AMR trait distributions are accounted for in the genotype-phenotype associations, even though the forecasting indicators operate at national scale. Second, the associations between AMR trait prevalence and national-level indicators identified in the forecasting pipeline reflect broad temporal trends that are consistent at national scale across the study period and are presented and interpreted as such throughout.

To fully address the Reviewer's question, we have conducted a supplementary analysis extracting province-level GDP per capita time series from the NBS China Statistical Yearbook (National Bureau of Statistics of China. China Statistical Yearbook. Beijing: NBS; 2022) for the 15 provinces represented in our dataset, calculating the coefficient of variation across provinces over the study period 2006–2022 (Supplementary Data 11). This confirms the substantial geographic heterogeneity flagged by the reviewer and quantifies the degree to which the national-level indicator approach smooths over provincial differences. Province-level time series for many of the other indicators used in our forecasting, particularly antibiotic consumption, governance metrics, and several health indicators, are not currently available in the public databases from which our data were drawn, limiting our ability to extend this analysis to all indicators. We have strengthened the limitations paragraph of the Discussion, accordingly, noting this explicitly and identifying province-level indicator integration as a key priority for future work that would enable regionally targeted AMR forecasts of direct relevance to provincial surveillance and policy. The manuscript has been edited in the Result Lines 319-322, Supplementary Legends, and Discussion lines 650-671.

Results Lines 319-322: ...quantified using 96 measurable indicators, see Supplementary Data 10. We note that these indicators were collected at national level; province-level GDP per capita data for the 15 study provinces over the study period are provided in Supplementary Data 11 to contextualise the geographic heterogeneity that national-level indicators smooth over.

Supplementary Data legend: **Supplementary Data 11.** Province-level GDP per capita for the 15 Chinese provinces represented in the study dataset, 2006–2022. Data extracted from the National Bureau of Statistics of China Statistical Yearbook (National Bureau of Statistics of China. China Statistical Yearbook. Beijing: NBS; 2022). The coefficient of variation (CV) across provinces is reported for each year to quantify the degree of geographic heterogeneity in this socioeconomic indicator over the study period. National level GDP per capita is also included for comparison but not included in the CV calculation.

Discussion Lines 650-671:

A further limitation of our forecasting analysis relates to the use of national-level aggregate values for socioeconomic, environmental, and health indicators to represent all 15 provinces included in the study. China exhibits substantial geographic heterogeneity across all indicator categories used: per capita GDP varies more than fourfold across provinces, and Guangxi, from which the majority of food worker isolates in our dataset were collected, had a per capita GDP of approximately 52,399 yuan in 2022, approximately 40% below the national average of 87,385 yuan reported for that year (National Bureau of Statistics of China. China Statistical Yearbook. Beijing: NBS; 2022), reflecting its status as one of the economically less developed provinces in our dataset relative to coastal provinces such as Shandong and Guangdong. Province-level variation in healthcare workforce density and health expenditure efficiency is similarly substantial across China (Shi Y, *et al*. *Frontiers in Public Health*. 2022;10:879698. doi:10.3389/fpubh.2022.879698), and within-province variation in antibiotic consumption has been documented even within single provinces (Yin J et al. Front Pharmacol. 2021 Nov 22;12:790817. doi: 10.3389/fphar.2021.790817). We note that province is explicitly included as a random effect in all GLMM models, with province ICC (0.149) among the largest of the four random effects, indicating that province-level variance in AMR trait prevalence is accounted for in the genotype-phenotype associations. Nevertheless, province-level time series for many of the indicators used in our forecasting, particularly antibiotic consumption, governance metrics, and several health indicators, are not currently available in the public databases from which our data were drawn. A supplementary analysis extracting province-level GDP per capita for the 15 study provinces from the NBS China Statistical Yearbook (National Bureau of Statistics of China. China Statistical Yearbook. Beijing: NBS; 2022) is provided in Supplementary Data 11, quantifying the geographic heterogeneity that national-level indicators smooth over. Future studies incorporating province-level indicator data, as these become available through expanding national surveillance databases, would enable regionally targeted AMR forecasts of direct relevance to provincial public health policy.

9. As many of the AMR traits characterized in this study are transferrable across bacterial species, do the authors think that any of the forecasting results might be applicable to future AMR trends in other foodborne bacteria?

We thank the reviewer for this forward-looking question, which touches on one of the most important broader implications of our findings. Many of the high-risk AMR traits identified and forecast in this study, including $bla_{CTX\text{-}M}$ variants, fluoroquinolone resistance genes (*qnrS1*, *qnrB17*), aminoglycoside resistance determinants, and phenicol resistance genes such as *floR*, are not exclusive to *Salmonella enterica*. They are carried on mobile genetic elements, particularly plasmids and insertion sequences, that are known to transfer horizontally across a wide range of Gram-negative foodborne pathogens, including *Escherichia* coli (Wang, Z. et al. Animals 12, 3191 DOI: 10.3390/ani12223191, 2022; Yang, L. et al. Nature Food 3, 197-205, DOI:10.1038/s43016-022-00470-6, 2022; Jiang, H. et al. BMC Microbiology 25, 76, DOI:10.1186/s12866-025-03794-y, 2025), *Campylobacter* spp. (Shah, V et al., Microbial Pathogenesis 206, 107796, DOI: 10.1016/j.micpath.2025.107796, 2025), *Klebsiella pneumoniae* (Tang, F. et al. American Journal of Infection Control 52, 1043-1051, DOI: 10.1016/j.ajic.2024.05.011, 2024; Sun, W. et al. Microbiology Spectrum 14, e02269-02225,

DOI:10.1128/spectrum.02269-25, 2025)., and other Enterobacteriaceae commonly associated with the food chain.

We therefore believe that several of our forecasting findings are likely to have relevance beyond *S. enterica*, with important caveats. The projected increase in plasmid-mediated quinolone resistance genes (*qnrB17*, *qnrS1*) and extended-spectrum beta-lactamase genes ($bla_{CTX-M-55}$, $bla_{CTX-M-65}$) in association with socioeconomic and healthcare indicators reflects broad ecological pressures, antibiotic consumption, urbanisation, healthcare expansion, that act on shared ecological and selective pressures across the food chain resistome, rather than being specific to the genomic background of any single bacterial species. Indeed, our previous work has demonstrated that clinically relevant ARGs and their associated mobile genetic elements are shared across *E. coli* and *S. enterica* co-inhabiting the same farm environments and food workers in China (Baker, M. et al. Nature Communications 15, 206, DOI: 10.1038/s41467-023-44272-1, 2024); Baker, M. et al. Nature Food 4, 707-720, DOI: 10.1038/s43016-023-00814-w, 2023), supporting the plausibility of cross-species applicability of these trends.

However, we urge caution in direct extrapolation for several reasons. First, the quantitative prevalence projections are calibrated specifically to MDR *S. enterica* in China's food chain and will reflect species-specific genomic backgrounds, ecological niches, and selective pressures that may differ substantially in other organisms. Second, *Campylobacter* spp., which are among the most clinically important foodborne pathogens globally, have a distinct resistome and different mechanisms of fluoroquinolone resistance (primarily chromosomal mutations in *gyrA* rather than plasmid-mediated genes), limiting the direct transferability of our fluoroquinolone forecasts to that species. Third, the indicator-trait associations identified here were derived from *S. enterica* prevalence data and may not hold quantitatively for other species even where the same traits are present.

We have added a paragraph to the Discussion acknowledging these broader implications while noting these caveats, as we believe this represents an important and underexplored dimension of food chain AMR surveillance that warrants dedicated future investigation applying equivalent frameworks to other priority foodborne pathogens.

Discussion Lines 511-527

The AMR traits identified and forecast in this study are predominantly carried on mobile genetic elements capable of HGT across bacterial species. Many of the high-risk traits projected to increase, including bla_{CTX-M} variants, plasmid-mediated quinolone resistance genes, and aminoglycoside resistance determinants, have been documented across multiple Gram-negative foodborne pathogens sharing the same ecological niches as *S. enterica* in China's food chain, including *Escherichia* coli (Wang, Z. et al. Animals 12, 3191 DOI: 10.3390/ani12223191, 2022; Yang, L. et al. Nature Food 3, 197-205, DOI:10.1038/s43016-022-00470-6, 2022; Jiang, H. et al. BMC Microbiology 25, 76, DOI:10.1186/s12866-025-03794-y, 2025), *Campylobacter* spp. (Shah, V et al., Microbial Pathogenesis 206, 107796, DOI: 10.1016/j.micpath.2025.107796, 2025), *Klebsiella* pneumoniae (Tang, F. et al. American Journal of Infection Control 52, 1043-1051, DOI: 10.1016/j.ajic.2024.05.011, 2024; Sun, W. et al. Microbiology Spectrum 14, e02269-02225, DOI:10.1128/spectrum.02269-25, 2025). The socioeconomic, healthcare, and antibiotic consumption pressures identified as correlates of AMR trait prevalence in our study act on the food chain resistome broadly, suggesting that the directional trends forecast here may have relevance beyond *S. enterica*. This is consistent with our previous work demonstrating shared ARGs and mobile genetic elements across *S. enterica* and *E. coli* co-inhabiting the same farm environments and food worker populations in China (Baker, M. et al. Nature Communications 15, 206, DOI: 10.1038/s41467-023-44272-1, 2024; Baker, M. et al. Nature Food 4, 707-720, DOI: 10.1038/s43016-023-00814-w, 2023). Nevertheless, direct quantitative extrapolation to other species requires caution, as prevalence projections are calibrated to *S. enterica*-specific genomic and ecological contexts. Applying equivalent surveillance and forecasting frameworks to other priority foodborne pathogens particularly *E. coli, Campylobacter spp., and K. pneumoniae*, represents an

important direction for future work and would provide a more complete picture of the trajectory of the food chain resistome under the socioeconomic and environmental pressures projected to 2050.

10. What specific added value does the gene- and indicator-level AMR prediction provided in this study have over more generic class-based risk assessments in terms of clinical, public health, or policy decisions?(Remarks on code availability)

We thank the reviewer for this important question, which allows us to articulate the translational value of our gene- and indicator-level framework more explicitly than in the original text. We would argue the added value operates across four distinct and interconnected levels, each grounded directly in the findings of this study and aligned with current international frameworks.

1. Clinical: mechanistic shifts invisible at class level

Genes within the same antibiotic class can have fundamentally different resistance mechanisms, mobility profiles, and fitness costs, with direct clinical consequences that class-level assessments cannot distinguish. Our data provide a concrete example: within fluoroquinolone resistance, mutation-based resistance (*gyrA* 87) is projected to decline over the next 25 years, while plasmid-mediated genes (*qnrB17*, *qnrS1*) are projected to rise. These two mechanisms differ profoundly in their clinical implications. Chromosomal mutation-based resistance is transmitted vertically and is therefore lineage-dependent, while plasmid-mediated quinolone resistance (PMQR) genes are horizontally transferable across serovars, hosts, and settings at frequencies much higher than chromosomal mutations, enabling spread across distantly related bacterial lineages independently of clonal expansion. Given that fluoroquinolones remain first-line treatments for invasive Salmonella infections, a class-level assessment suggesting stable or declining overall fluoroquinolone resistance could create false reassurance while the more dangerous, horizontally transmissible component grows. Similarly, within third-generation cephalosporin resistance, $bla_{\text{CTX-M-64}}$ and $bla_{\text{TEM-1}}$ are projected to rise by up to 20% by 2050, while $bla_{\text{CTX-M-65}}$, a closely related gene in the same class, is projected to remain below 5%. These genes differ in their associated plasmid contexts, co-resistance profiles, and capacity for spread, distinctions that are clinically relevant for empirical treatment guidance and infection control but completely obscured by class-level aggregation.

2. Public health surveillance: prioritisation of specific resistance determinants

Gene-level forecasting enables evidence-based prioritisation of specific resistance determinants for monitoring, rather than treating entire antibiotic classes as homogeneous surveillance targets. This matters because surveillance resources in most settings, and particularly in LMICs, are finite. Our framework identifies which specific genes are likely to increase in prevalence and are linked to rising socioeconomic and health indicators, enabling surveillance systems to focus on the highest-risk determinants. For example, the divergent trajectories of *tet(A)* and *tet(M)* versus *tet(B)*, reflecting differences in chromosomal integration versus mobile element carriage, would be invisible at the class level but are directly relevant to distinguishing clonal spread from horizontal dissemination in food chain surveillance. This distinction has operational consequences: clonal spread requires source control and infection containment, while horizontal spread requires monitoring of mobile element dynamics across multiple hosts and settings. Our risk stratification framework further adds value by integrating clinical relevance, mobility, and cross-host spread into a single score per gene, enabling tiered surveillance responses proportional to actual transmission risk, an approach aligned with the recently updated WHO Global Action Plan on AMR (2026–2036)(World Health Organization. Draft updated global action plan on AMR (2026–2036), Adopted at the Seventy-ninth World Health Assembly, 23 May 2026, A79/5 Add. 2, https://apps.who.int/gb/ebwha/pdf_files/WHA79/A79_5Add2-en.pdf, 2026)., which explicitly calls for strengthened digital technologies, including artificial intelligence, next-generation sequencing use in surveillance within a One Health framework.

3. Policy: identifying the specific structural drivers of specific resistance genes

The indicator-level associations provide policy-actionable specificity that class-based assessments cannot deliver. By linking individual resistance genes to specific socioeconomic, health, and antibiotic

consumption drivers, our framework identifies which structural levers are relevant to which resistance threats, and, critically, reveals when different genes within the same class require different policy responses. The rise of PMQR genes (*qnrB17, qnrS1*) is associated with urbanisation, healthcare spending, aging population, and total antibiotic consumption, suggesting that China's 2020 ban on quinolone premixes as veterinary feed additives, a stewardship measure targeting agricultural use, may be insufficient on its own, because the drivers of plasmid-mediated resistance extend beyond the veterinary sector into healthcare infrastructure and urban demographic change. A class-level framework would indicate fluoroquinolone resistance as a policy target without revealing this mechanistic and driver-level complexity. Similarly, the gene-specific associations with governance indicators (e.g. government effectiveness) alongside socioeconomic indicators suggest that AMR policy cannot be reduced to antibiotic stewardship alone but must engage with broader health system governance, a finding consistent with recent umbrella review evidence (Ljungqvist G. *et al. One Health* **20**, 100986, DOI:10.1016/j.onehlt.2025.100986, 2025) that national action plans have thus far failed to adequately address socioeconomic drivers of AMR. Furthermore, our finding that food workers carry significantly more high-risk traits than food sources, including last-resort resistance genes absent from food itself, points to a specific occupational group requiring targeted intervention, an insight that aggregate class-level foodborne AMR surveillance would not yield.

4. Forecasting: the necessity of gene-level resolution for meaningful projections

Finally, class-level forecasting conflates genes with different trajectories into a single trend, which can be misleading in both direction and magnitude. Our data show that within tetracycline resistance, *tet(A)* and *tet(M)* are forecast to increase while *tet(B)* is forecast to decrease, a divergence explained by differences in chromosomal versus mobile element carriage in the food chain. Aggregated at the class level, these opposing trends would partially cancel, potentially suggesting stable tetracycline resistance when in fact the mobile, more transferable component is rising. The same pattern is observed across aminoglycoside, β-lactam combination agent, folate, macrolide, and phenicol resistance classes. Gene-level forecasting linked to specific indicators therefore provides both the directionality and the mechanistic explanation needed for intervention planning.

We have expanded the Discussion to make these points more explicit, as detailed below.

Discussion lines 546-579:

The gene- and indicator-level resolution of our framework provides added value over class-based AMR risk assessments across clinical, public health, and policy dimensions. Clinically, genes within the same antibiotic class can differ fundamentally in their resistance mechanisms, mobility, and fitness costs, with direct consequences for treatment and containment. Our findings illustrate this clearly: within fluoroquinolone resistance, mutation-based resistance (*gyrA* 87) and plasmid-mediated resistance genes (q*nrB17, qnrS1*) are projected to follow opposite trajectories. Because plasmid-mediated quinolone resistance (PMQR) genes are transferable horizontally across serovars and hosts at frequencies much higher than chromosomal mutations, their rise represents a qualitatively different and more serious public health threat than an equivalent rise in mutation-based resistance, a distinction entirely obscured by class-level assessment. Similarly, within third-generation cephalosporin resistance, $bla_{\text{CTX-M-64}}$ and $bla_{\text{TEM-1}}$ are projected to increase by up to 20%, while $bla_{\text{CTX-M-65}}$ is projected to remain below 5%, and within tetracycline resistance, *tet(A)* and *tet(M)* are forecast to increase while *tet(B)* decreases, divergences that would partially cancel at the class level, potentially creating false reassurance or masking genuinely rising threats.

For public health surveillance, gene-level forecasting enables evidence-based prioritisation of specific resistance determinants for monitoring, an important practical consideration in resource-limited settings where surveillance capacity cannot target all resistance equally. Our risk stratification framework, integrating clinical relevance, cross-host mobility, and serovar spread into a single score per genomic trait, provides a basis for tiered surveillance responses proportional to actual transmission risk, aligned with the call for strengthened genomic surveillance within the recently updated WHO Global Action Plan on AMR (2026–2036) (World Health Organization. Draft updated global action plan on AMR

(2026–2036), Adopted at the Seventy-ninth World Health Assembly, 23 May 2026, A79/5 Add. 2, https://apps.who.int/gb/ebwha/pdf_files/WHA79/A79_5Add2-en.pdf, 2026).

For policy, the indicator-level associations reveal that different genes within the same antibiotic class are driven by different structural factors, and therefore require different interventions. The rise of PMQR genes is linked to urbanisation, healthcare spending, and demographic change alongside antibiotic consumption, suggesting that veterinary stewardship measures alone are insufficient and that complementary interventions targeting horizontal transmission in healthcare and urban food systems are needed. More broadly, the association of specific resistance genes with governance indicators alongside socioeconomic ones suggests that effective AMR policy must engage with health system governance rather than antibiotic use alone, consistent with recent evidence that national action plans have thus far inadequately addressed the structural socioeconomic drivers of AMR. Together, these layers of resolution transform gene-level surveillance data into a mechanistically grounded, policy-actionable framework for targeted AMR intervention.

The code appears to fully reproduce the results of the paper, and provides a comprehensive README outlining dependencies and usage instructions.
We thank the reviewer for taking the time to check this

Reviewer #3 (Remarks to the Author):

This manuscript by Baker et al. investigates high-risk AMR genomic traits in MDR Salmonella across China's food chain. The authors developed an ARG risk-ranking framework and used machine learning to project AMR trends through 2050.Werne Although the study addresses an important topic and uses a unique large-scale dataset, several methodological limitations reduce confidence in the findings. In its current form, the analyses do not fully capture the richness of the dataset or provide the depth of insight needed to support the broader conclusions.

We thank Reviewer 3 for their careful reading of the manuscript and for raising points that allow us to clarify and strengthen the presentation of our methods and findings. We address each comment in turn below. Where the reviewer has identified genuine opportunities to improve clarity, we have made corresponding revisions to the manuscript. Where comments reflect misunderstandings of the methodology as already described, we provide detailed clarification and direct the reviewer to the relevant sections of the original submission.

Major comments:
Question 1. The ML framework is difficult to follow. Although many steps are described, the overall structure of the analysis is not sufficiently clear. In particular, the authors should clearly define the input features, outcome variables, and evaluation strategy for each model. At present, it appears that AMR genomic traits may have been included as model features, while resistance or susceptibility was treated as the outcome. If so, the authors need to explain how phenotypic resistance or susceptibility was defined and quantified. Otherwise, the analysis risks circularity, essentially using AMR-associated features to predict AMR status. The authors should clarify whether the model is predicting independent phenotypic resistance outcomes from genomic predictors, or whether it is partly using the same AMR information to predict itself. It is also not clear how training/testing was split. Importantly, a key limitation is the lack of external validation for the ML models. The current training-testing framework may not be sufficient to establish model generalizability, particularly if the same dataset was used for feature selection, model tuning, and performance evaluation.

1A. ML framework clarity. Regarding the Reviewer's comment on the ML framework being difficult to follow.
We appreciate this feedback and have reviewed the Methods section carefully, we have taken the following steps to further improve clarity:

We have revised the Methods section (Lines 1027-1042, and below) to ensure that input features, outcome variables, and evaluation strategy are explicitly stated at the outset of the ML subsection. We have also updated Figures S1-S3, which provide detailed flowcharts of the complete pipeline, to ensured that the terminology in the text is fully consistent with that used in the Methods.

Methods Line 1027-1042:
Input features: For each antibiotic-specific model, the input features comprised encoded AMR genomic traits derived from whole-genome sequence data, including ARG presence/absence, ARG-carrying insertion sequence (IS) patterns, and ARG-carrying plasmid content, as described above (see Encoding of ARGs and ARG-carrying MGEs). The outcome variable for each model was the binary phenotypic resistance or susceptibility of each isolate to the corresponding antibiotic, as determined independently by antimicrobial susceptibility testing (AST) using CLSI M100 30th Edition breakpoints (see Antimicrobial susceptibility testing). Genomic features and phenotypic outcomes therefore represent entirely independent data modalities. In summary, model evaluation followed a stratified group k-fold cross-validation (k=5) framework, with folds grouped by PopPUNK cluster to prevent population structure leakage, repeated across 50 independent trials. Performance was assessed using ROC-AUC, PR-AUC, accuracy, sensitivity, specificity, and precision, reported as mean and standard deviation across folds and trials. Only models achieving AUC >0.7 were retained. Within retained models, genomic features were selected based on three criteria applied jointly: effect size (OR ⩾2), statistical significance (lower 95% CI ⩾1), and cross-validation stability (positive association with resistance in ⩾80% of trials). This is detailed below.

1B. Regarding the Reviewer's comment "the authors need to explain how phenotypic resistance or susceptibility was defined and quantified"

We respectfully note that this information was present in the original manuscript (Lines 781–783, Antimicrobial susceptibility testing section), where we state explicitly: *"Minimum inhibitory concentrations were interpreted to the Clinical and Laboratory Standards Institute criteria M100, 30th Edition breakpoints to define resistance."* The specific antimicrobial panels used (NMIC413 and NMIC801) and their constituent agents are listed in full. For the publicly available isolates, AST data were taken directly from the original published sources, which used equivalent CLSI breakpoints; this is now stated explicitly in the revised Methods. To further aid the reader, we have added a sentence clarifying how MIC values were converted to binary resistant/susceptible classifications for model input. We have now edited the Methods Lines 781-796.

Methods: 781-796: Minimum inhibitory concentrations (the lowest concentration of antibiotic that prevents visible growth) were interpreted to the Clinical and Laboratory Standards Institute criteria M100, 30th Edition breakpoints to define resistance. MIC values were interpreted as susceptible where the MIC value was ≤ the susceptible threshold, values ≥ the resistance threshold were interpreted as resistant and values between those were interpreted as intermediate. *Escherichia coli* ATCC 25922 was used as a quality control strain during the antimicrobial susceptibility testing; see **Supplementary Data 1.** For publicly available isolates, AST data were taken directly from the original published sources. We reviewed the methods of each source publication to confirm the CLSI M100 edition used for breakpoint interpretation (27th, 28th, 30th, or 31st edition, depending on publication date) and cross-checked the relevant breakpoints for each antibiotic tested with our models across these editions (Table S2). Breakpoints for all antibiotics included in our analysis were unchanged across these editions for

Salmonella spp., confirming interpretive consistency between the publicly available and newly generated AST data. For streptomycin and moxifloxacin no breakpoints are given in any of the CLSI M100 editions, so we relied on the RSI interpretations given in the original source. For both newly sequenced and publicly available isolates resistant to ≥ 3 antibiotic classes were defined as MDR, and isolates resistant to < 3 as non-MDR.

1C. Regarding the Reviewer's comment on "The analysis risks circularity, using AMR-associated features to predict AMR status.

We must respectfully but firmly correct this mischaracterisation of our methodology, as it reflects a misunderstanding of the study design rather than a methodological flaw.

The input features to our ML models are AMR genomic traits (the presence or absence of specific ARGs, ARG-carrying insertion sequences, and ARG-carrying plasmids, identified from whole-genome sequence data). The outcome variable is the observed phenotypic resistance or susceptibility of each isolate, as determined independently by antimicrobial susceptibility testing (AST) using standardised CLSI breakpoints on cultured bacteria. These are entirely independent data modalities: one is derived from genomic sequencing, the other from culture-based laboratory testing. No phenotypic data were used as model inputs, and no genomic features were derived from phenotypic outcomes.

This is precisely the design of genotype-to-phenotype association studies, a well-established and widely published analytical framework in AMR genomics (see, for example, Baker M. et al. Nature Communications 15, 206, DOI: 10.1038/s41467-023-44272-1, 2024;

Baker M. et al., Nature Food 4, 707-720, DOI:10.1038/s43016-023-00814-w, 2023, and the broader WGS-based AMR prediction literature, Baker M. et al., Cell Genomics, online 101273, DOI:10.1016/j.xgen.2026.101273, 2026). The explicit purpose of such models is to quantify the strength of genotype–phenotype associations that is, to identify which specific genomic configurations are most predictive.

We have added a clarifying statement to the Methods section (Lines 1027-1034) making the independence of genomic features and phenotypic outcomes explicit and note that this is stated clearly in the original text: *" to identify genomic traits that are not only present across all sources but are also strongly predictive of observed resistance profiles"* (Lines 230–232).

Methods Lines 1027-1034: Input features: For each antibiotic-specific model, the input features comprised encoded AMR genomic traits derived from whole-genome sequence data, including ARG presence/absence, ARG-carrying insertion sequence (IS) patterns, and ARG-carrying plasmid content, as described above (see Encoding of ARGs and ARG-carrying MGEs). The outcome variable for each model was the binary phenotypic resistance or susceptibility of each isolate to the corresponding antibiotic, as determined independently by antimicrobial susceptibility testing (AST) using CLSI M100 30th Edition breakpoints (see *Antimicrobial susceptibility testing*). Genomic features and phenotypic outcomes therefore represent entirely independent data modalities.

1D. Regarding the Reviewer's comment that it is not clear how training/testing was split.

We note that the cross-validation procedure is described in detail in the original Methods (Lines 1072–1099), including: stratified group k-fold cross-validation (k=5), grouping by PopPUNK cluster to prevent population structure leakage, 50 repeated trials, and evaluation metrics computed on held-out test folds. A final model was then fitted on all data to obtain stable feature importance estimates (OR and confidence intervals), as is standard practice in this type of analysis. We have revised the Methods to ensure this sequence is presented more clearly and sequentially and have updated Figure S2 to label training and test sets explicitly at each stage.

1E. A key limitation is the lack of external validation for the ML models. The current training-testing framework may not be sufficient to establish model generalizability, particularly if the same dataset was used for feature selection, model tuning, and performance evaluation.

We thank the reviewer for raising this important point. We agree that external validation is a critical test of model generalisability, and we have addressed this through two new validation analyses described below, in addition to the cross-validation framework already employed.

We conducted two complementary validation analyses beyond the existing cross-validation pipeline.

Within-group temporal holdout. We partitioned the full MDR dataset into a training set comprising all pre-2021 isolates (n=2,286) and a held-out validation set comprising all isolates collected in 2021–2022 (n=250). The original GLMM methodology was applied unchanged to the training set only, including cross-validation and final model fitting. The resulting fixed-effect coefficients were then applied directly to the held-out 2021–2022 isolates, with no refitting, to obtain genuinely prospective predictive performance estimates. Models with poor performance in the original analysis (AUC < 0.7; MXF and NAL) were excluded from summary reporting, though their results are included in Supplementary Data 8 for completeness. This validation demonstrated consistently high AUC values for the majority of models (Supplementary Data 8). Excluding DOX, for which the small holdout sample size (n=36) precluded stable estimation (see below), the mean AUC was 0.94 ± 0.04 (sensitivity 0.86 ± 0.14, specificity 0.85 ± 0.15), confirming that model performance remains high when applied to data not used during model fitting. External AUC values were generally higher than those obtained from cross-validation, which we attribute to the cross-validation framework's deliberately more stringent design, testing across held-out genomic lineages via PopPUNK-grouped folds, rather than a temporal split alone. A small number of models showed reduced performance on specific metrics. STR and CHL showed reduced sensitivity or specificity attributable to extreme class imbalance (≥95% resistant) within the validation set, while still retaining AUC ≥0.76. For KAN, the within-group holdout set (n=39) contained no susceptible isolates (100% resistant), meaning AUC and precision could not be calculated and are reported as undefined rather than reduced. The corresponding accuracy, PR-AUC, sensitivity and precision metrics of 1.0 should be interpreted with this caveat: in the absence of any negative class in the test set, this value does not constitute evidence of strong discriminatory performance and is not directly comparable to values calculated on holdout sets containing both classes. DOX showed a markedly reduced AUC (0.51) in this validation set, close to that expected by chance; given the very small number of available isolates for this antibiotic in the holdout period (n=36, with only 6 susceptible isolates), we consider this most likely attributable to insufficient sample size for stable estimation in this specific subgroup rather than to a failure of the underlying model, which achieved high training-set and original full-model AUCs (0.997 and 0.978, respectively) for this antibiotic.

Out-of-group external validation. We additionally applied the final fitted models, without refitting, to an independent published dataset of human and food *Salmonella* isolates collected in Guangdong province between 2017 and 2024, comprising diarrhoeal patients from sentinel hospitals and food sources including pre-packaged ready-to-eat foods, cooked foods, pastry products, baked goods, pre-prepared dishes, and vegetables (Su et al., *Microbiology Spectrum*, 2026). We note that although all patients in this dataset had diarrhoeal *Salmonella* infections, the original study did not establish a direct epidemiological link to the food chain, and the dataset differs substantially from ours in geography, source composition, and baseline resistance prevalence. We therefore present this analysis as a stress-test of model robustness under conditions of genuine dataset shift, rather than as a like-for-like external validation. Despite these differences, the fitted models retained meaningful discriminatory performance: of five comparable antibiotic models, three achieved AUCs equal to or exceeding the original model's performance (relative AUC: AZM 1.05, CAZ 1.04, SXT 0.98), and two showed modestly reduced but still within-range performance (relative AUC: CIP 0.84, CTX 0.89), Supplementary Data 9. Sensitivity and specificity followed a similar pattern. A reduction in precision was observed for AZM and CTX, which we attribute to substantial shifts in baseline resistance

prevalence between datasets (AZM: 26% to 5%; CTX: 43% to 16%) rather than to a failure of the underlying genotype–phenotype associations; this illustrates the kind of dataset shift expected when any fitted model is applied outside its original epidemiological context, and underscores the importance of local recalibration when applying the pipeline to new settings.

Context: availability of a directly comparable Chinese food-chain cohort. To determine whether a more directly comparable cohort was available, we performed a systematic search of the literature and public sequence repositories for an independent dataset of *Salmonella* sequences from food workers or handlers in China. Despite extensive searching, no such dataset has been published. In PubMed, the search terms "(*Salmonella enterica*) AND (China) AND (Whole genome sequencing) AND ('Food workers' OR 'food handlers' OR 'asymptomatic')" retrieved only four results: one study of reptiles, one study of a single serovar (Newport) without AST data, and two case reports each with only a single food worker/handler sample. A global search adding susceptibility testing terms retrieved no results, and a looser search without the AST requirement produced 32 results, none with linked WGS and AST data. As our study, and our previously linked study (Lu X. et al., Nature Communications 15, 10238, DOI: 10.1038/s41467-024-54405-9, 2024). represents the first large-scale genomic study of food workers in China, a directly comparable control dataset could not be identified. We therefore designed the within-group temporal holdout and out-of-group stress-test described above as the most rigorous validation analyses achievable given current data availability, rather than as a substitute for what we agree would be the ideal test.

Why a non-Chinese cohort would not have been the more appropriate test. We also note that there are two distinct, complementary questions one might ask about generalisability, and a non-Chinese cohort and a Chinese cohort each answer a different one. A non-Chinese dataset would test whether the genomic feature-to-phenotype associations we report reflect general microbial biology versus China-specific population structure or sampling artefacts, a valid and important question in its own right. A Chinese cohort, by contrast, tests whether the models are fit for their stated surveillance purpose within China's food chain, where *Salmonella* AMR epidemiology, antibiotic usage patterns, and the occupational structure underlying food worker surveillance are highly context-specific and vary substantially even at the provincial level (see our response to Reviewer 2, Question 8). Given that our study's explicit aim is the latter, we prioritised the Chinese-context stress-test (Su et al. dataset) as the most relevant available test of fitness for purpose, while agreeing that a non-Chinese comparison would be a valuable, complementary line of future enquiry into the general biological transferability of the underlying associations. A non-Chinese dataset, drawn from a setting with very different antibiotic stewardship regimes, food production systems, and regulatory frameworks, would test a different and, we would argue, less directly relevant question: whether genomic feature-to-phenotype associations generalise across fundamentally different selective pressures, rather than whether our models are robust for their intended surveillance purpose within China's food chain. The Su et al. dataset, while not a food worker cohort, shares the same national regulatory, epidemiological, and antibiotic-use context, making it a considerably more relevant stress-test than a non-Chinese cohort would have been.

Supporting evidence from the existing cross-validation framework and companion pipeline. Beyond the two new analyses above, we note that the cross-validation framework already employed, stratified group k-fold with grouping by PopPUNK cluster, is specifically designed to evaluate performance on held-out genomic lineages not seen during training, providing a further, complementary internal test of generalisability across population structure. The ML pipeline used here is also directly adapted from Baker et al. (Baker M. et al., Cell Genomics, Online 101273, DOI:10.1016/j.xgen.2026.101273, 2026), a global study applying an identical framework across 16 bacterial species, approximately 400,000 AST phenotypes, and approximately 46,000 genomes, providing independent evidence of the pipeline's broader methodological generalisability.

Taken together, we believe the temporal holdout validation, the out-of-group stress-test against an independent published Chinese dataset, the existing lineage-stratified cross-validation, and the validated companion pipeline collectively provide strong evidence of model robustness. We continue

to recognise, and have stated explicitly in the revised Discussion, that prospective validation using a directly comparable food worker cohort from an independent province or time period remains a priority for future work as such data become available.

We have updated the manuscript accordingly: Methods Lines 1101-1110, Results Lines 271-309, Discussion Lines 689-715, and added Supplementary Data 8 and 9.

Methods, Lines 1101-1110:

External validation analyses. A within-group validation analysis was conducted using isolates from 2021 and 2022 as a hold-out set. Models were fitted on the remaining (pre-2021) isolates, and the fitted coefficient estimates were applied to the hold-out set to obtain predictive performance estimates without refitting. Similarly, an independent out-of-group dataset was retrieved, comprising human and food *Salmonella* isolates collected in Guangdong province between 2017 and 2024 (Su et al., *Microbiology Spectrum*, 2026), including diarrhoeal patients from sentinel hospitals and food sources comprising pre-packaged ready-to-eat foods, cooked foods, pastry products, baked goods, pre-prepared dishes, and vegetables. Models were fitted on the original (full) dataset, and the fitted coefficient estimates were applied to the out-of-group dataset to obtain predictive performance estimates.

Results, Lines 271-309:

We sought to test the predictive performance of the models beyond the cross-validation already employed within the pipeline. The full dataset was partitioned into a training set comprising all pre-2021 isolates (n=2,286) and a within-group validation set comprising all 2021–2022 isolates (n=250). The original GLMM methodology was applied unchanged to the training set only, including cross-validation and final model fitting. The resulting fixed-effect coefficients were applied to the within-group validation set to calculate predictive performance metrics. Models with poor performance in the original analysis (AUC < 0.7; MXF and NAL) were excluded from summary reporting, though their results are included in Supplementary Data 8 for completeness. Excluding DOX, for which the small holdout sample size (n=36) precluded stable estimation (see below), the mean AUC was 0.94 ± 0.04 (sensitivity 0.86 ± 0.14, specificity 0.85 ± 0.15), confirming that model performance remains high when applied to data not used during model fitting. External AUC values were generally higher than those obtained from cross-validation, reflecting the more stringent design of the original pipeline, which used stratified grouped k-fold cross-validation to assess performance generalised to novel genomic lineages. STR and CHL showed poorer performance on some metrics, attributable to extreme class imbalance (≥95% resistant) within the validation set rather than to deficiencies in the underlying model. DOX showed reduced performance attributable to small sample size (n=36) within the validation set. For KAN, the within-group holdout set (n=39) contained no susceptible isolates (100% resistant), meaning AUC and precision could not be calculated and are reported as undefined rather than reduced; the corresponding accuracy, PR-AUC, precision and sensitivity metrics of 1.0 should be interpreted with this caveat rather than as evidence of strong discriminatory performance

As a further stress-test, we applied the final fitted models to an independent dataset of human and food *Salmonella* isolates collected in Guangdong province between 2017 and 2024 (Su et al., *Microbiology Spectrum*, 2026), comprising diarrhoeal patients from sentinel hospitals and food sources including pre-packaged ready-to-eat foods, cooked foods, pastry products, baked goods, pre-prepared dishes, and vegetables. We note that although all patients had diarrhoeal *Salmonella* infections, the original study did not present evidence of a direct link to the food chain. While this external dataset differs substantially from ours in geography, source composition, and resistance prevalence, it provides a useful stress-test of the modelling approach under conditions of dataset shift. Despite these differences, the fitted models retained discriminatory performance in this altered epidemiological context. Of five comparable antibiotic models, three achieved AUCs comparable to or exceeding the full model AUC (relative AUC: AZM 1.05, CAZ 1.04, SXT 0.98), and the remaining two showed slightly reduced AUCs that nonetheless remained within the standard deviation of the original model performance (relative

AUC: CIP 0.84, CTX 0.89). Sensitivity and specificity were similarly maintained or improved in three models and reduced in the other two (Supplementary Data 6 and 9). Some models showed reduced precision (AZM, CTX), driven by large shifts in the proportion of resistance between datasets (AZM: 26% to 5%; CTX: 43% to 16%), illustrating the effect of dataset shift and the importance of recalibration when applying the pipeline in altered epidemiological contexts.

Discussion, Lines 689-715:

While our cross-validation framework, stratified by PopPUNK cluster to evaluate performance across held-out genomic lineages, already provides a rigorous internal assessment of model generalisability, we additionally validated our fitted models against two independent test sets not used in model fitting: a temporal holdout comprising isolates collected in 2021–2022, and an out-of-group dataset of human and food *Salmonella* isolates from Guangdong province (Su et al., *Microbiology Spectrum*, 2026). Both analyses demonstrated that model performance was largely maintained, with some expected attenuation in the out-of-group analysis attributable to shifts in baseline resistance prevalence between datasets. A systematic search of the literature and public sequence repositories confirmed that no directly comparable Chinese food worker or broader food-chain cohort with matched genomic and phenotypic data currently exists in the public domain, underscoring that our study represents the first large-scale genomic dataset of this kind. We note that the same analytical pipeline has been independently applied and validated at global scale across multiple pathogens and countries in a companion study (Baker M. et al., Cell Genomics, online 101273, DOI:10.1016/j.xgen.2026.101273, 2026), supporting its broader methodological generalisability. Overall, these analyses demonstrate that our fitted models are robust to new data from within the study population, and retain meaningful discriminatory performance when applied to new data from outside it, despite substantial differences in geography, source composition, and resistance prevalence. This supports the validity of the underlying genotype–phenotype associations beyond the specific dataset used for fitting. We would, however, caution against direct clinical or surveillance deployment of the fixed, fitted coefficients in a new population without local recalibration, particularly where baseline resistance prevalence differs substantially, as our precision results illustrate. Our aim is therefore to provide a reusable analytical pipeline that can be re-fitted in different epidemiological contexts as local data accumulate, rather than a single static model intended for direct cross-population deployment. As whole-genome sequencing becomes increasingly integrated into China's national AMR surveillance infrastructure, prospective validation using independent provincial or temporal cohorts, particularly from food worker or other occupational health screening programmes, remains an important priority for future work.

Supplementary Data 8:

Antibiotic model	Within-group Validation Dataset (Isolates from 2021,2022; n=250) - Performance Metrics						Training dataset (n=2286) AUC	Original model (n=2536) AUC	Antibiotic specific training sample size (R/S phenotypes only included)	Antibiotic specific within -group validation sample size (R/S phenotypes only included)	Resistance prevalence - training dataset	Resistance prevalence - within-group validation dataset
	AUC	PR_AUC	Accuracy	Sensitivity	Specificity	Precision						
AZI	0.94548148	0.87010451	0.85641026	0.86666667	0.85333333	0.63934426	0.751±0.250	0.815±0.210	483	195	27%	23%
AZM	0.98090815	0.97732426	0.83516484	0.94117647	0.77192982	0.71111111	0.813±0.069	0.802±0.116	1736	91	25%	37%
CAZ	0.96157442	0.95946372	0.97165992	0.89285714	0.9947644	0.98039216	0.820±0.086	0.826±0.126	1900	247	23%	18%
CFZ	0.94661458	0.9849241	0.890625	0.9375	0.75	0.91836735	0.780±0.148	0.790±0.125	1401	64	29%	75%
CHL	0.98285048	0.99905852	0.96311475	0.97835498	0.69230769	0.9826087	0.825±0.113	0.840±0.089	2232	244	86%	95%
CIP	0.9093254	0.95031856	0.84027778	0.75	0.96666667	0.96923077	0.711±0.118	0.757±0.100	2214	144	31%	58%
CRO	0.93111111	0.91380952	0.94545455	0.7	1	1	0.719±0.094	0.727±0.096	2062	55	20%	18%
CTX	0.87655626	0.82450682	0.81538462	0.68181818	0.88372093	0.75	0.556±0.252	0.702±0.217	266	195	49%	33%
CXM	0.87380952	0.82870236	0.88461538	0.5	0.97619048	0.83333333	0.758±0.074	0.760±0.085	1562	52	27%	19%
DOX	0.50735294	0.9582296	0.94444444	1	0	0.94444444	0.997±0.013	0.978±0.033	95	36	83%	94%
FEP	0.95245245	0.94370045	0.89010989	0.78378378	0.96296296	0.93548387	0.782±0.087	0.774±0.166	1722	91	21%	41%
GEN	0.98123782	0.98929387	0.93548387	0.9122807	0.97222222	0.98113208	0.867±0.120	0.884±0.076	2206	93	40%	61%
KAN		1	1	1		1	0.690±0.390	0.716±0.394	153	39	83%	100%
LVX	0.9321513	0.95209498	0.86956522	0.86666667	0.87234043	0.86666667	0.646±0.155	0.642±0.186	1774	92	16%	49%
MIN	0.94805195	0.98630593	0.91304348	0.96363636	0.71428571	0.92982456	0.715±0.093	0.717±0.096	1331	69	34%	80%
MXF	0.57738095	0.59278499	0.90909091	0.28571429	1	1	0.630±0.135	0.634±0.135	1626	55	14%	13%
NAL	0.96127804	0.7964083	0.79487179	0.94117647	0.77697842	0.34042553	0.605±0.260	0.643±0.223	503	156	61%	11%
SAM	0.86645963	0.98818351	0.94949495	0.98913043	0.42857143	0.95789474	0.747±0.102	0.754±0.094	952	99	65%	93%
STR	0.76190476	0.99665481	0.66492147	0.66666667	0.5	0.99212598	0.608±0.185	0.707±0.182	364	191	76%	99%
SXT	0.97219771	0.99413674	0.956	0.95631068	0.95454545	0.98994975	0.786±0.143	0.805±0.130	2286	250	72%	82%
TOB	0.99897119	0.99707602	0.97777778	1	0.96296296	0.94736842	0.840±0.111	0.848±0.087	1324	45	39%	40%

Supplementary Data 8 Legend: Within-group external validation predictive performance metrics. Predictive performance for the within-group dataset, using coefficient estimated from the fitted training dataset (pre 2021 data) *S. enterica* models. The performance metrics were AUC, PR-AUC, accuracy (TP+TN/(P+N)), sensitivity (true positive rate: TP/P), specificity (true negative rate: TN/N), AUC and precision. Also given is the training data set AUC mean and standard deviation; the full model AUC mean and standard deviation, the antibiotic specific number of isolates included in the training and within-group validation datasets (after intermediate phenotypes and missing phenotypes were removed); and the proportion of resistant isolates (R/(R+S)) in the training and within-group validation datasets. For KAN, the within-group validation set contained no susceptible isolates (100% resistant, n=39); specificity and AUC are therefore undefined and reported as blank cells, and the corresponding accuracy, precision, PR-AUC and sensitivity values should be interpreted accordingly.

Supplementary Data 9:

Antibiotic model	Out-of-group Validation Dataset (n=319) - Performance Metrics						Original model (n=2536)	Antibiotic specific training sample size (R/S phenotypes only included)	Antibiotic specific out-of-group validation sample size (R/S phenotypes only included)	Resistance prevalence - training dataset	Resistance prevalence - Out-of-group validation dataset
	AUC	PR_AUC	Accuracy	Sensitivity	Specificity	Precision	AUC				
AZI	NOT IN OUT-OF-GROUP DATASET										
AZM	0.84276316	0.20726933	0.79937304	0.86666667	0.79605263	0.17333333	#N/A	1827	319	0.259	0.047
CAZ	0.85710643	0.75758827	0.93589744	0.79245283	0.96525097	0.82352941	#N/A	2147	312	0.182	0.17
CFZ	NOT IN OUT-OF-GROUP DATASET										
CHL	NOT IN OUT-OF-GROUP DATASET										
CIP	0.63369627	0.53881898	0.7606383	0.30769231	0.93382353	0.64	0.757±0.100	2358	188	0.324	0.277
CRO	NOT IN OUT-OF-GROUP DATASET										
CTX	0.63060295	0.36156029	0.83647799	0.53846154	0.89473684	0.5	0.702±0.217	461	318	0.427	0.164
CXM	NOT IN OUT-OF-GROUP DATASET										
DOX	NOT IN OUT-OF-GROUP DATASET										
FEP	NOT IN OUT-OF-GROUP DATASET										
GEN	NOT IN OUT-OF-GROUP DATASET										
KAN	NOT IN OUT-OF-GROUP DATASET										
LVX	NOT IN OUT-OF-GROUP DATASET										
MIN	NOT IN OUT-OF-GROUP DATASET										
MXF	NOT IN OUT-OF-GROUP DATASET										
NAL	NOT IN OUT-OF-GROUP DATASET										
SAM	NOT IN OUT-OF-GROUP DATASET										
STR	NOT IN OUT-OF-GROUP DATASET										
SXT	0.78848331	0.72355567	0.73354232	0.78947368	0.69354839	0.64814815	0.805±0.130	2536	319	0.728	0.417
TOB	NOT IN OUT-OF-GROUP DATASET										

Supplementary Data 9 Legend: Out-of-group external validation predictive performance metrics. Predictive performance for the out-of-group dataset, using coefficient estimated from the fitted MDR S. enterica models. The performance metrics were AUC, PR-AUC, accuracy (TP+TN/(P+N)), sensitivity (true positive rate: TP/P), specificity (true negative rate: TN/N), AUC and precision. Also given is the full model AUC mean and standard deviation, the antibiotic specific number of isolates included in the training (original MDR dataset) dataset and out-of-group validation datasets (after intermediate phenotypes and missing phenotypes were removed); and the proportion of resistant isolates (R/(R+S)) in the training and out-of-group-group validation datasets.

Question 2. The proposed ARG risk-ranking system is insufficiently justified and appears underdeveloped in relation to existing antimicrobial resistance gene risk-assessment frameworks (e.g., Zhang et al, 3032). The authors should clearly explain how this system aligns with, differs from, or improves upon prior frameworks. At present, it is difficult to determine whether the ranking provides new conceptual or practical value, or whether it largely repackages criteria that have already been established. Without a direct comparison to existing ARG risk frameworks and a transparent explanation of scoring, weighting, and validation, the proposed ranking system risks appearing subjective and difficult to reproduce. This substantially limits confidence in its novelty, rigor, and utility for AMR surveillance or risk prioritization.

We thank the reviewer for this comment, which has prompted us to make explicit a comparison to existing frameworks that was previously only implicit in the manuscript. We address each sub-point below. Regarding novelty and relationship to existing frameworks: the reviewer references "Zhang et al., 2032" we assume this refers to Zhang et al., Nature Communications, 2021 (Zhang AN *et al*. Nature Communications 12, 4765, DOI:https://doi.org/10.1038/s41467-021-25096-3, 2021). Our framework explicitly incorporates the Zhang et al. Rank I criteria as one component of the clinical relevance score (Methods, Lines 934-942) and also incorporates the WHO AWARE classification (Methods Lines 934-942). However, our framework extends these prior approaches in three important respects that we now make explicit in a revised Discussion paragraph: (i) it operates at the level of individual AMR traits (specific ARG–MGE combinations) rather than ARGs alone, capturing the mobility context of each resistance element; (ii) it integrates both inter-host and inter-serovar mobility as dimensions of transmission likelihood, neither of which is captured in the Zhang et al. framework; and (iii) it is specifically calibrated to the One Health food chain context in China, incorporating antibiotic usage patterns specific to that setting (Table S1).

To our knowledge, based on review of the major published ARG risk-ranking frameworks (including Zhang et al., 2021, and the WHO AWARE classification), this combination of MGE-context, empirical cross-host mobility, and setting-specific calibration is not commonly integrated into a single scoring framework, though we cannot exclude the existence of more specialised, less widely cited frameworks doing so in adjacent fields such as environmental or wastewater AMR surveillance. Regarding subjectivity and reproducibility: the scoring criteria are fully defined in the Methods (Lines 230–278) and are fully pre-specified and rule-based in their application: once defined, every trait is scored identically according to fixed, objective, and reproducible criteria with no post hoc adjustment or case-by-case judgement. We acknowledge that, as with any scoring framework, the initial choice of thresholds (e.g., ≥2 serovars for inter-serovar mobility, ≥3 antibiotic classes for MDR) reflects a designed methodological decision; however, these thresholds are consistent with established conventions in the AMR literature (MDR definition, Magiorakos AP. et al., Clin Microbiol Infect 18, 268-281, DOI:10.1111/j.1469-0691.2011.03570.x, 2012). . The inter-host mobility score (c) is assigned based on the documented presence of each trait across defined source categories in our dataset. The inter-serovar mobility score (d) is binary, based on presence in ≥2 serovars. The clinical relevance score is the sum of the antibiotic class usage score (*a*, defined by Table S1 and the AWARE classification) and the MDR score (*m*, binary based on ≥3 antibiotic classes) (Lines 949-950). All thresholds and criteria are pre-specified and applied uniformly across all traits, with no *post hoc* adjustment. We have now additionally transferred the risk scoring calculations to Python and deposited them in the Github repository, ensuring complete computational reproducibility for this component of the analysis.

We have added a paragraph to the Discussion comparing our framework to existing ARG risk assessment approaches, clarifying how it relates to and extends prior work, including the Zhang et al. (2021) framework which is incorporated as one component of our clinical relevance score.

Discussion Lines 406-424:
Several frameworks have been proposed to assess the risk posed by AMR genes, most notably the omics-based framework of Zhang et al. (2021), which ranks ARGs according to human enrichment, mobility, and association with ESKAPE pathogens, and the WHO AWARE classification, which stratifies antibiotic classes by their importance for human medicine and their role in driving resistance. Our risk assessment framework explicitly incorporates both of these established approaches as components of the clinical relevance dimension of our scoring matrix: the Zhang et al. Rank I criteria contribute directly to the ARG impact score (a), and the WHO AWARE classification informs the antibiotic class usage score across all five tiers. However, our framework extends these prior approaches in three important respects. First, it operates at the level of AMR traits specifically including ARG–MGE combinations rather than ARGs in isolation, capturing the genomic mobility context of each resistance element, which is critical for assessing transmission potential. Second, it explicitly quantifies inter-host and inter-serovar mobility as dimensions of likelihood of spread, using empirical distribution data across One Health source types and serovars within our dataset, neither of which is captured in

existing frameworks. Third, it is calibrated to the specific antibiotic usage patterns and One Health food chain context of China, incorporating source-type distribution data from human, animal, food, and environmental isolates collected over 16 years. To our knowledge, no existing ARG risk framework simultaneously integrates MGE context, empirical cross-host mobility, and setting-specific antibiotic usage in this way.

Question 3. The authors attempted to project the impact of antibiotic use, population growth, healthcare spending, and urbanization on future AMR patterns. However, the analysis does not first provide a thorough assessment of how these factors explain the current AMR patterns observed in the dataset. This weakens the basis for long-term projection. Without demonstrating that these variables are meaningfully associated with present-day AMR variation, it is difficult to interpret their projected effects over the next 20 years. As currently presented, the forecast provides limited mechanistic or explanatory insight, because the reader is asked to accept future predictions before the drivers of current patterns have been rigorously established.

We thank the reviewer for this comment, but we must respectfully clarify that the manuscript already provides exactly the assessment the reviewer describes as missing, and we are concerned this may reflect an incomplete reading of the Methods and Results rather than a genuine gap in our analysis.

We do provide a thorough assessment of how these factors explain current AMR patterns, and we demonstrate that these variables are meaningfully associated with present-day AMR variation, before any forecasting is performed. This is the explicit purpose of Steps 1–6 of our forecasting pipeline (Methods, Lines 1126-1167): for each of the 205 ML-selected, high-risk AMR traits, we calculate historical trait prevalence from 2006–2022 (the "current AMR patterns observed in the dataset"), and test its association with each of 96 socioeconomic, health, antibiotic-consumption, governance, and climate indicators using Pearson correlation, retaining only pairs with $|r| > 0.5$. These associations are then validated against a permutation-based null distribution (1,000 permutations per pair) with Benjamini–Hochberg FDR correction (threshold 10%), specifically to guard against spurious historical associations driven by chance or shared temporal trends. Of the 205 high-risk traits, 165 were found to be significantly and robustly associated with at least one present-day indicator under this framework (Results, Lines 328–334), and we report the specific indicator categories most strongly associated with current AMR trait prevalence (Figure 2A; Results, Lines 338–340). This is, precisely, a rigorous, present-day, indicator-by-indicator assessment of association with current AMR variation, established before any projection is made. Only once this present-day association is established and validated does the pipeline proceed to forecast indicator trends and, in turn, project trait prevalence forward (Steps 7–8, Methods Lines 1169–1185).

We have additionally strengthened confidence in the specificity and robustness of these present-day associations through four new analyses conducted in response to a related comment from Reviewer 1, demonstrating that: indicator-indicator collinearity is generally low (mean $|r| = 0.237$) and does not undermine the independence of the reported associations; 78.2% of forecastable traits are supported by indicators from two or more independent categories, rather than a single shared trend; results are robust to indicator redundancy when re-run using one representative indicator per correlated cluster (95% of traits retained); and a principal component analysis confirms that AMR trait associations are not concentrated on a single dominant axis of indicator variation (permutation $p = 0.999$). Full details are provided in our response to Reviewer 1, Question 3.

We would also note that while our manuscript is primarily a large-scale association and forecasting study, it provides mechanistic insight grounded directly in these present-day associations, which we believe directly addresses the Reviewer's concern that the forecast offers "limited mechanistic or explanatory insight." For example, our finding that mutation-based fluoroquinolone resistance (*gyrA* 87) and plasmid-mediated resistance genes (*qnrB17*, *qnrS1*) are associated with present-day indicators in opposing directions leads us to propose a specific, biologically-grounded mechanistic interpretation:

namely, that the known fitness costs of *gyrA* mutations under reduced selective pressure, combined with the differential transmissibility of horizontally mobile plasmid-borne resistance, together explain the projected divergence between these two resistance mechanisms (Discussion, Lines 668–687). Similarly, our analysis of the genomic location of *tet(A)*, *tet(M)*, and *tet(B)* provides a mechanistic explanation, grounded in present-day genomic data, for their divergent projected trajectories (Discussion, Lines 656–664). These are not post hoc speculations detached from the data; they are interpretations directly motivated by the present-day, indicator-validated associations described above. We have now clarified this in the Discussion Lines 678-688.

Discussion Lines 678-688: We further note that the associations identified between AMR trait prevalence and socioeconomic, health, and climate indicators, while statistically robust and FDR-corrected, are correlational in nature: the forecasting pipeline itself quantifies association, not causal mechanism, between these indicators and AMR trait prevalence. Where we offer biologically-grounded interpretation of specific findings elsewhere in this Discussion, for example regarding the divergence between mutation-based and plasmid-mediated fluoroquinolone resistance, or the genomic-location-dependent trajectories of *tet(A)*, *tet(M)*, and *tet(B)*, these interpretations draw on independent evidence (e.g., known fitness costs of resistance mutations, documented antibiotic stewardship policy timelines, and genomic location data generated in this study) and are presented as plausible explanatory context for the patterns observed, rather than as conclusions established by the forecasting pipeline's correlational methodology.

Question: 4. Although diverse Salmonella serotypes were included in the analyses, the role of serotype was not sufficiently addressed. This is an important limitation because Salmonella serotypes differ substantially in host range, host adaptation, ecological niche, food production chain association, and AMR potential. Therefore, conclusions generalized across all isolates may be biased by the serotypes that dominate the dataset. B The study would be strengthened by stratifying AMR profiles by serotype and assessing whether patterns of AMR emergence, persistence, and dissemination differ across serotypes. In addition, the analysis should evaluate how environmental, host-associated, and food production-related factors interact with serotype to shape AMR profiles. C Without this level of resolution, it is difficult to determine whether the reported AMR patterns reflect broad species-level trends or are driven primarily by dominant serotype-specific effects.

We thank the reviewer for raising this point. We would like to clarify how serotype (serovar)-related confounding was addressed in the original submission, and we have additionally conducted a new quantitative analysis directly testing for dominant-serotype bias, descry
described below:
First, PopPUNK clusters (Methods, Lines 1049-1050) were used as random effects in all mixed-effects models, explicitly accounting for lineage structure. PopPUNK clusters provide higher-resolution population structure than serotype designation: serovar and ST were highly correlated, (Cramér's V,=0.85, *p*-value=2.2×10^{-16} Line 108-109), and PopPUNK clusters were confirmed not to mix serovars. The odds ratios reported for each genomic feature therefore represent within-lineage effect sizes, conditional on population structure. This is the appropriate and most powerful approach to control for lineage confounding more rigorous than stratifying by serotype directly, as it operates at finer resolution.
Second, the risk assessment framework explicitly deprioritises traits present in only one serovar (inter-serovar mobility score, Methods Lines 925–928). Traits classified as high-risk (Rank IV or V) are by definition those present across multiple serovars, and 98% of the 84 traits forecast to increase were found across two or more serogroups (Results, Lines 539-540). The concern that findings are "driven primarily by dominant serotype-specific effects" is therefore directly addressed by design.
Third, the cross-validation was stratified by PopPUNK cluster, meaning that model performance was evaluated on held-out lineages. Features that are retained under this regime are precisely those that generalise across lineage boundaries including across serovars.

We have now quantified the distribution of selected ML traits across serotypes (relative to the overall serotype distribution in the dataset) and confirmed that selected features are not disproportionately associated with the dominant serovars. Specifically, after normalising be the serotype frequency, the median trait enrichment in the ML selected features was 1.02, i.e. consistent with the baseline serotype distribution. In contrast, the non-selected traits had a median enrichment of 0.12, indicating a strong skew towards rare serotypes. This analysis has been added to the Methods Lines 1113-1119 and Results Lines 261-270.

We accept the reviewer's point that a dedicated analysis of how serotype interacts with source type and AMR profile would be an interesting extension of this work but note that this is beyond the stated scope of the study. We have added a sentence to the Discussion acknowledging this as a direction for future work.

Methods Lines 1113-1119: *Assessment of serotype bias*. To assess dominant-serovar bias in the ML-selected features, a dominance enrichment score per trait was calculated as the fraction of trait occurrences in the dominant serovars divided by the corresponding fraction of isolates in the dominant serovars. The median dominance trait score across selected traits was compared to the median dominance trait score across none selected traits. Dominant serovars were defined at those comprising ≥ 5% of isolates in the dataset. Very rare serovars (present in ≤ 2 isolates) that would bias the analysis were filtered out.

Results Lines 261-270: ML-selected features were not disproportionately associated with the dominant serovars. Specifically, after normalising by the serovar frequency, the median trait enrichment in the ML selected features was 1.02, i.e. consistent with the baseline serovar distribution. In contrast, the non-selected traits had a median enrichment of 0.12, indicating a strong skew towards rare serovars. This indicates that ML-selected, high-risk traits are distributed across serotypes in proportion to their overall prevalence in the dataset, providing no evidence of dominant-serovar bias. The lower enrichment score observed for non-selected traits reflects the fact that many such traits are rare, isolate-specific findings restricted to less common serovars, which were appropriately excluded by the cross-validation stability criterion (⩾80% of CV trials) rather than by any serovar-related selection bias.

Discussion Lines 528-545:

A potential concern in any study spanning multiple Salmonella serovars is whether reported AMR patterns reflect broad species-level trends or are driven primarily by dominant serotype-specific effects. We addressed this at multiple levels of the analysis. First, population structure of which serovar is a major component, given the high correlation between serovar and sequence type in our dataset (Cramer's V=0.85, p-value $< 2.2 \times 10^{-16}$) was explicitly modelled as a random effect in all GLMM models via PopPUNK cluster membership, which provides higher-resolution lineage classification than serovar designation alone. The odds ratios reported for each genomic feature therefore represent within-lineage effect sizes, conditional on population structure, and cannot be attributed to serovar composition differences between source types. Second, the risk assessment framework explicitly deprioritises traits confined to a single serovar through the inter-serovar mobility score, such that all traits classified as high-risk (Rank IV or V) are by definition present across multiple serovars. Consistent with this, 98% of the 84 traits forecast to increase were found across two or more serogroups. Third, cross-validation stratified by PopPUNK cluster ensures that retained features generalise across lineage boundaries: features selected under this regime are precisely those not confined to dominant lineages. To further address this point, we performed a *post hoc* analysis quantifying the serovar distribution of all ML-selected traits, confirming that selected traits are not disproportionately associated with the dominant serovars relative to their prevalence in the full dataset.

Other comments:

Line 128-129: Clarify that 279 MDR and 138 non-MDR are newly acquired isolates.

We thank the reviewer for this suggestion. We have revised Lines 765-766 to read: "A total of 279 newly sequenced MDR and 138 newly sequenced non-MDR S. enterica isolates were tested for antimicrobial susceptibility..." This clarifies that these isolates represent the new sequences generated for this study, distinct from the publicly available isolates described above.

Methods Lines 765-766: A total of 279 newly sequenced MDR and 138 newly sequenced non-MDR S. enterica isolates were tested…

Lines 128-148: Clarification of MDR/non-MDR classification for public isolates.

We thank the reviewer for raising this important point of clarification. For the 279 newly sequenced MDR and 138 newly sequenced non-MDR isolates, MDR status was determined directly from antimicrobial susceptibility testing performed in this study using CLSI M100 30th Edition breakpoints. For publicly available isolates, MDR classification was based on antimicrobial susceptibility testing metadata reported in the original source publications and associated database records, using the same MDR definition. We have added the following sentence to the Methods Lines 795-796:

Methods Lines 795-796: For both newly sequenced and publicly available isolates resistant to ≥3 antibiotic classes were defined as MDR and isolates resistant to < 3 as non-MDR.

Lines 156-161: Assembly quality metrics and completeness/contamination cutoffs.

We thank the reviewer for highlighting this gap. Assembly quality metrics and completeness/contamination thresholds were applied to all genomes included in the analysis, including publicly available assemblies downloaded from NCBI and NGDC. We have added the following to the Methods.

Methods lines 815-821: For newly sequenced genomes, assemblies were retained if they met the following quality thresholds: N50 ≥ 15kb, genome completeness ≥ 80% and contamination ≤ 5% as assessed by CheckM v1.2.2. For publicly available assemblies: Sequences associated to Bioproject PRJNA1082900 were obtained as pre-assembled genome sequences (assembled using Spades v3.15.5) from the National Genomics Data Center. These were also assessed against the same completeness and contamination thresholds described above prior to inclusion; assemblies failing these criteria were excluded.

Line 163: Difference between phylogeny here and Lines 219-227.

We apologise for this inconsistency, we ran only one phylogeny, this was a redundancy within the Methods. We thank the reviewer for identifying this, the first reference to phylogeny at original manuscript line 163 has now been removed.

Lines 163-165: Redundant description of ARG profiling.

We agree with the reviewer that this description is redundant. The sentence on original manuscript lines 163-165 referring to screening against CARD and ISfinder databases has been removed, as this is described in full detail in the subsequent dedicated section (Screening of annotated genes against ARG and MGE databases, Lines 837-846).

Line 277: What is a and what is m?

We thank the reviewer for identifying this lack of clarity. The variables a and m are defined earlier in the Methods (Lines 934-947) but we acknowledge that the final equation at Line 949 appears without

immediate reference to these definitions, which may cause confusion. We have revised Line 949 to read: "The final clinical relevance score was the sum of the maximum ARG impact score (a, defined above) and the multidrug resistance score (m, defined above): Clinical Relevance = a + m." This ensures the reader can immediately locate the definitions of both variables.

Lines 282-283: Difference between mobility and HGT; how HGT was detected; HGT not spelled out.

We thank the reviewer for these points. We have made the following corrections and clarifications:

We acknowledge that the terms "mobility" and "HGT" were used imprecisely in this sentence. In our framework, "mobility" refers to the genomic context of AMR traits, specifically their association with mobile genetic elements (insertion sequences and plasmids), which is inferred from the genomic location of ARGs relative to IS elements and plasmid sequences, as described in the plasmid reconstruction and mobile ARG cassette identification sections. We do not directly detect or quantify HGT events, and the reference to HGT has been removed from Lines 968-970 and replaced with: "...to capture the co-occurrence of multiple resistance mechanisms, including chromosomal resistance, IS-mediated mobility, and plasmid-associated dissemination, and their additive effects on resistance phenotype.".

As the term HGT has now been removed here and was the only instance of its use it has not been spelled out in the revised manuscript.

Line 502: P-values reported as 0.

Response:
We thank the reviewer for this important correction. P-values that appear as 0 reflect the numerical precision limits of the statistical implementation rather than true zero probability values. We have revised all such instances throughout the manuscript to report these as $p = 2.2 \times 10^{-16}$ (the minimum value representable in double-precision floating point arithmetic in Python/SciPy) or for bootstrap *p*-values, 0.0001 (the minimum value representable for the estimated bootstrap *p* values, with 10000 iterations).

Code availability: Bioinformatics code largely missing.

Response:
We thank the reviewer for this observation. The bioinformatics pipeline described in the Methods (genome assembly, quality control, ARG and IS identification, plasmid reconstruction, pangenome analysis, and phylogenetic inference) relied on established, publicly available tools (fastp, SPAdes, QUAST, CheckM, Bakta, Panaroo, IQ-TREE, Abricate, MLST, SISTR, plasmidVerify, Plasmer, MOB-suite, minimap2), each of which is individually cited with version numbers and parameter settings reported in full in the Methods. These software packages, although largely open source, were created and published by others, and so cannot be added to our code repository. However, the with version numbers and parameters, the outputs are now fully reproducible.
