# Supplementary material for: High-risk antimicrobial resistance genomic traits in multidrug-resistant Salmonella across China’s food chain: projections to 2050
Source: Nat Commun. 2026 Oct 20;17:10744. doi: 10.1038/s41467-026-78346-7 (PMC13683381; doi:10.1038/s41467-026-78346-7)
Supplement: Supplementary file 3 [file 41467_2026_78346_MOESM1_ESM.pdf]

**Supplementary Information for High-risk antimicrobial resistance genomic traits in multidrug-resistant *Salmonella* across China's food chain: projections to 2050**

**Supplementary Tables S1 to S3**

**Supplementary Figures S1 to S31**

**Supplementary Results**

**Table S1.** *Antibiotic class usage – China*. List of antibiotic classes used in China and their respective usage: not widely used in humans or animals (1), only used in humans (2), and widely used in humans and animals (3).

| Antibiotic class | Usage type |
|---|---|
| **aminoglycosides** | 3 |
| **Carbapenems** | 2 |
| **Cephalosporins** | 3 |
| **Cephamycins** | 3 |
| **diaminopyrimidine** | 1 |
| **fluoroquinolones** | 3 |
| **Glycopeptides** | 2 |
| **Glycylcyclines** | 2 |
| **lincosamides** | 2 |
| **macrolides** | 3 |
| **monobactams** | 3 |
| **nitrofurans** | 2 |
| **Penems** | 2 |
| **Peptides** | 3 |
| **phenicols** | 3 |
| **phosphonic acid** | 2 |
| **rifamycin** | 1 |
| **streptogramin A** | 1 |
| **streptogramins** | 1 |
| **streptogramin B** | 1 |
| **sulfonamides** | 3 |
| **Sulfones** | 2 |
| **Tetracyclines** | 3 |

**Table S2:** *Clinical breakpoints for antibiotics used in GLM antibiotic models.* Clinical breakpoints for Salmonella all antibiotics used in the GLMMs, taken from the CLSI editions used for the interpretation of MICs in each of the studies used in our datasets, specifically, CLSI M100 editions 27,28,30,and 31.

| | **M100 27th Ed. Breakpoints** | | | **M100 28th Ed. Breakpoints** | | | **M100 30th Ed. Breakpoints** | | | **M100 31st Ed. Breakpoints** | | |
|---|---|---|---|---|---|---|---|---|---|---|---|---|
| | S | I | R | S | I | R | S | I | R | S | I | R |
| *AZI* | ≤16 | | ≥32 | ≤16 | | ≥32 | ≤16 | | ≥32 | ≤16 | | ≥32 |
| *AZM* | ≤4 | 8 | ≥16 | ≤4 | 8 | ≥16 | ≤4 | 8 | ≥16 | ≤4 | 8 | ≥16 |
| *CAZ* | ≤4 | 8 | ≥16 | ≤4 | 8 | ≥16 | ≤4 | 8 | ≥16 | ≤4 | 8 | ≥16 |
| *CFZ* | ≤2 | 4 | ≥8 | ≤2 | 4 | ≥8 | ≤2 | 4 | ≥8 | ≤2 | 4 | ≥8 |
| *CHL* | ≤8 | 16 | ≥32 | ≤8 | 16 | ≥32 | ≤8 | 16 | ≥32 | ≤8 | 16 | ≥32 |
| *CIP* | ≤0.06 | 0.12-0.5 | ≥1 | ≤0.06 | 0.12-0.5 | ≥1 | ≤0.06 | 0.12-0.5 | ≥1 | ≤0.06 | 0.12-0.5 | ≥1 |
| *CRO* | ≤1 | 2 | ≥4 | ≤1 | 2 | ≥4 | ≤1 | 2 | ≥4 | ≤1 | 2 | ≥4 |
| *CTX* | ≤1 | 2 | ≥4 | ≤1 | 2 | ≥4 | ≤1 | 2 | ≥4 | ≤1 | 2 | ≥4 |
| *CXM* | ≤8 | 16 | ≥32 | ≤8 | 16 | ≥32 | ≤8 | 16 | ≥32 | ≤8 | 16 | ≥32 |
| *DOX* | ≤4 | 8 | ≥16 | ≤4 | 8 | ≥16 | ≤4 | 8 | ≥16 | ≤4 | 8 | ≥16 |
| *FEP* | ≤2 | 4-8 | ≥16 | ≤2 | 4-8 | ≥16 | ≤2 | 4-8 | ≥16 | ≤2 | 4-8 | ≥16 |
| *GEN* | ≤4 | 8 | ≥16 | ≤4 | 8 | ≥16 | ≤4 | 8 | ≥16 | ≤4 | 8 | ≥16 |
| *KAN* | ≤16 | 32 | ≥64 | ≤16 | 32 | ≥64 | ≤16 | 32 | ≥64 | ≤16 | 32 | ≥64 |
| *LVX* | ≤0.12 | 0.25-1 | ≥2 | ≤0.12 | 0.25-1 | ≥2 | ≤0.12 | 0.25-1 | ≥2 | ≤0.12 | 0.25-1 | ≥2 |
| *MIN* | ≤4 | 8 | ≥16 | ≤4 | 8 | ≥16 | ≤4 | 8 | ≥16 | ≤4 | 8 | ≥16 |
| *MXF* | | | | | | | | | | | | |
| *NAL* | ≤16 | | ≥32 | ≤16 | | ≥32 | ≤16 | | ≥32 | ≤16 | | ≥32 |
| *SAM* | ≤8/4 | 16/8 | ≥32/16 | ≤8/4 | 16/8 | ≥32/16 | ≤8/4 | 16/8 | ≥32/16 | ≤8/4 | 16/8 | ≥32/16 |
| *STR* | | | | | | | | | | | | |
| *SXT* | ≤2/38 | | ≥4/76 | ≤2/38 | | ≥4/76 | ≤2/38 | | ≥4/76 | ≤2/38 | | ≥4/76 |
| *TOB* | ≤4 | 8 | ≥16 | ≤4 | 8 | ≥16 | ≤4 | 8 | ≥16 | ≤4 | 8 | ≥16 |

**Table S3.** *ML-selected AMR traits in models with performance over 70% AUC based on the non-MDR S. enterica isolates.* AMR traits correlated to resistant-susceptibility profiles selected by the machine learning on models with an AUC over 70% for the non-MDR *S. enterica* isolates. For each trait, antibitoc model in which it was selected, the risk assessment category, the model coefficient, odds ratio, and 95% confidence intervals are given

| **Antibiotic model** | **AMR Trait** | **Risk** | **Coefficient** | **Odds_Ratio** | **OR_95CI_Lower** | **OR_95CI_Upper** |
|---|---|---|---|---|---|---|
| **TET** | *tet(B)* | Rank IV | 5.45 | 233.49 | 77.88 | 125.58 |
| **TET** | *tet(A)* | Rank IV | 5.21 | 183.51 | 111.29 | 134.65 |
| **TET** | *aph(3')-Ia* | Rank IV | 2.42 | 11.27 | 8.26 | 9.29 |
| **TET** | *tet(B)*_IS*10L*_IS*Kpn72*_IS*Vsa5*_IS*10R* | Rank IV | 2.19 | 8.97 | 4.95 | 6.38 |
| **TET** | *tet(M)* | Rank IV | 2.15 | 8.61 | 4.60 | 5.86 |
| **TET** | *sul2* | Rank IV | 1.78 | 5.94 | 5.10 | 5.49 |
| **TET** | *tet(B)*_IS*10L*_IS*Kpn72*_ISVsa5_IS*10R*_IS*1R*_IS*1B*_IS*1D*_IS*1A*_IS*1G*_IS*1SD*_IS*1S* | Rank IV | 1.67 | 5.33 | 3.36 | 4.07 |
| **TET** | *floR*_IS*5075*_IS*1006* | Rank IV | 1.43 | 4.19 | 2.74 | 3.12 |
| **TET** | *aadA7* | Rank IV | 1.43 | 4.17 | 2.89 | 3.42 |
| **TET** | *aadA7_sul1*_IS*6100* | Rank IV | 1.43 | 4.17 | 2.89 | 3.42 |
| **TET** | bla*CTX-M-65* | Rank IV | 1.39 | 4.02 | 2.57 | 2.89 |
| **TET** | bla*CTX-M-65*_IS*903B*_IS*903* | Rank IV | 1.39 | 4.02 | 2.57 | 2.89 |
| **TET** | *aac(6')-Ib-cr6* | Rank IV | 1.24 | 3.46 | 2.12 | 2.39 |
| **TET** | aph(3")-Ib_aph(6)-Id_sul2_IS*26*_IS*15DII*_IS*15DIV*_IS*15DI* | Rank IV | 1.16 | 3.20 | 1.99 | 2.28 |
| **TET** | *bla*TEM-1_Tn*2* | Rank V | 1.16 | 3.20 | 1.99 | 2.28 |
| **TET** | *aph(3")-Ib* | Rank IV | 1.16 | 3.18 | 3.24 | 3.54 |
| **TET** | *qnrS1*_IS*Kpn19* | Rank IV | 1.05 | 2.85 | 2.02 | 2.31 |
| **TET** | *aadA2_sul1*_IS*1326* | Rank II | 0.96 | 2.60 | 1.85 | 2.07 |

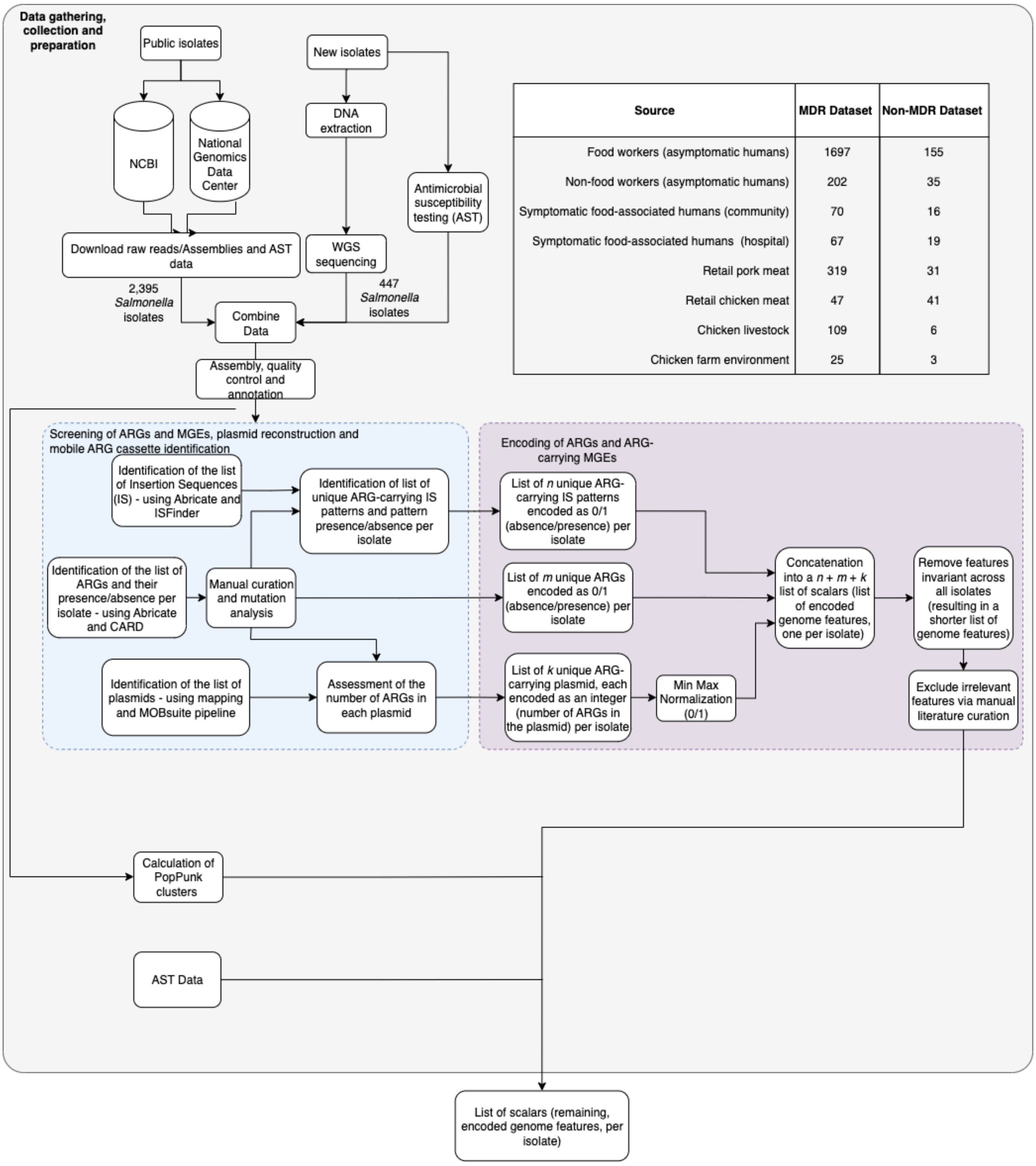


| Source | MDR Dataset | Non-MDR Dataset |
|---|---|---|
| Food workers (asymptomatic humans) | 1697 | 155 |
| Non-food workers (asymptomatic humans) | 202 | 35 |
| Symptomatic food-associated humans (community) | 70 | 16 |
| Symptomatic food-associated humans (hospital) | 67 | 19 |
| Retail pork meat | 319 | 31 |
| Retail chicken meat | 47 | 41 |
| Chicken livestock | 109 | 6 |
| Chicken farm environment | 25 | 3 |

**Figure S1. Data analysis and processing part 1**: Data gathering, collection and preparation. Activities are grouped into modules, represented as panels of different colours; the arrows represent the data flow. The following abbreviations are used National Center for Biotechnology Information (NCBI), whole genome sequencing (WGS), antimicrobial susceptibility testing (AST) antimicrobial resistance genes (ARGs), mobile genetic elements (MGEs).

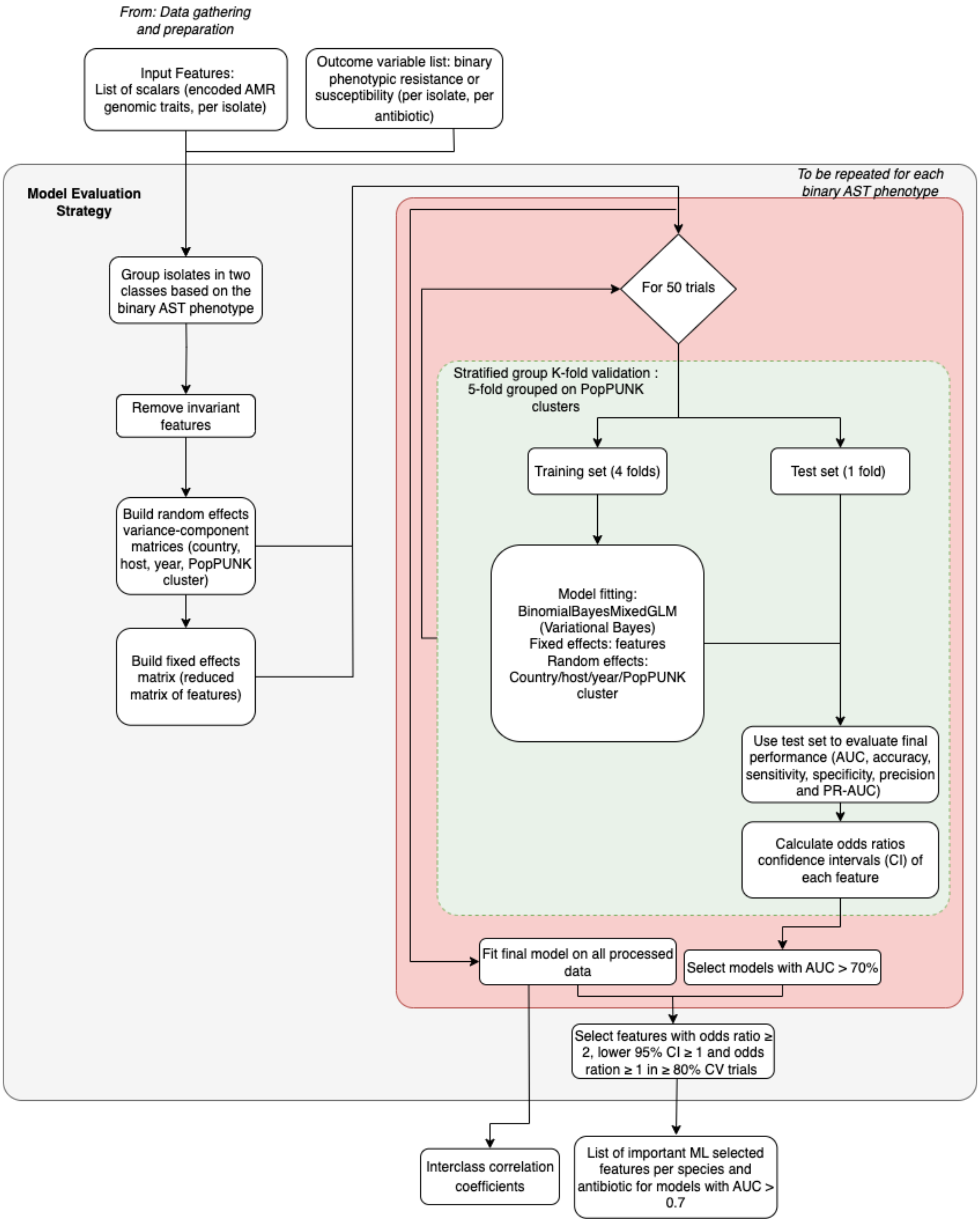


**Figure S2. Data analysis and processing part 2**: Machine learning classification of AMR phenotype. Activities are grouped into modules, represented as panels of different colours; the arrows represent the data flow. The following abbreviations are used antimicrobial resistance (AMR), area-under-the-curve (AUC), precision recall AUC (PR-AUC), cross validation (CV) confidence intervals (CI), machine learning (ML).

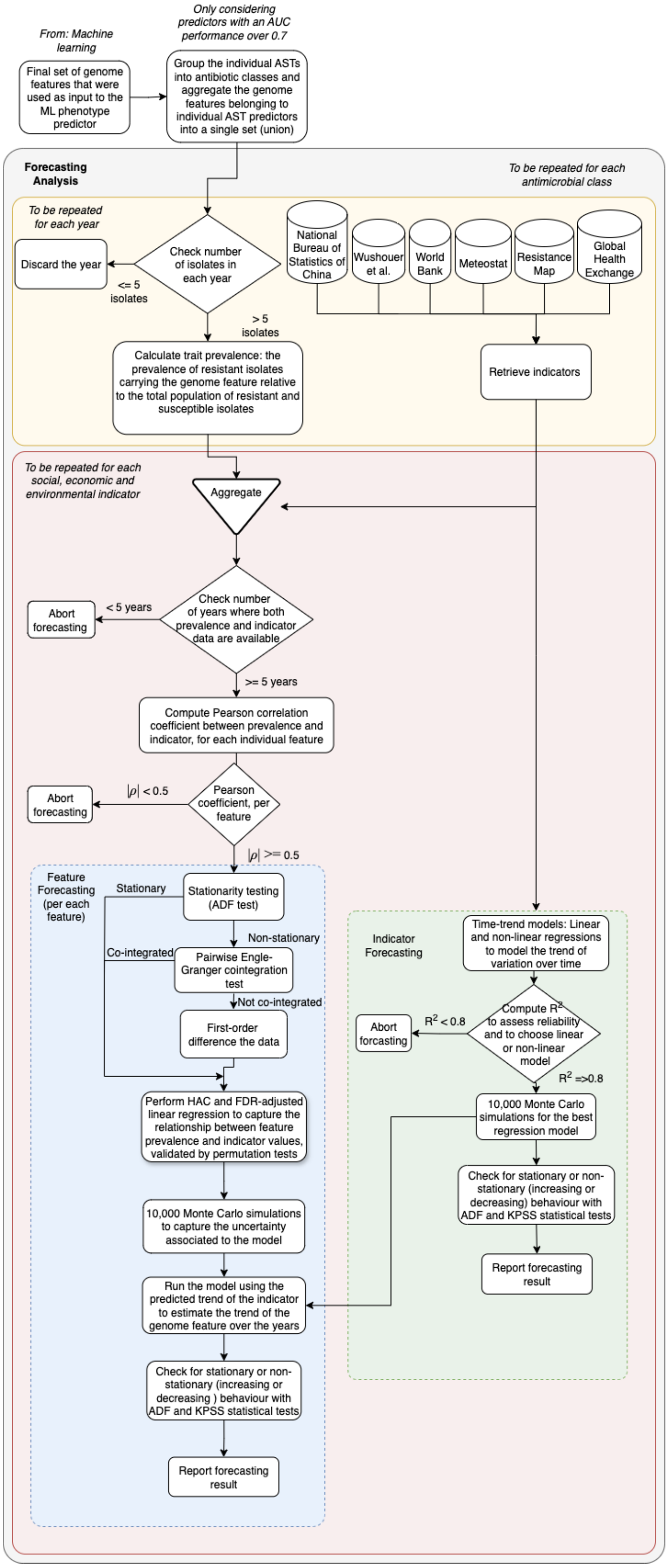


**Figure S3. Data analysis and processing part 3**: forecasting analysis. Activities are grouped into modules, represented as panels of different colours; the arrows represent the data flow. The following abbreviations are used area-under-the-curve (AUC), machine learning (ML), augmented Dickey Fuller (ADF), Kwiatkowski-Phillips-Schmidt-Shin (KPSS), heteroskedasticity and autocorrelation consistent (HAC).

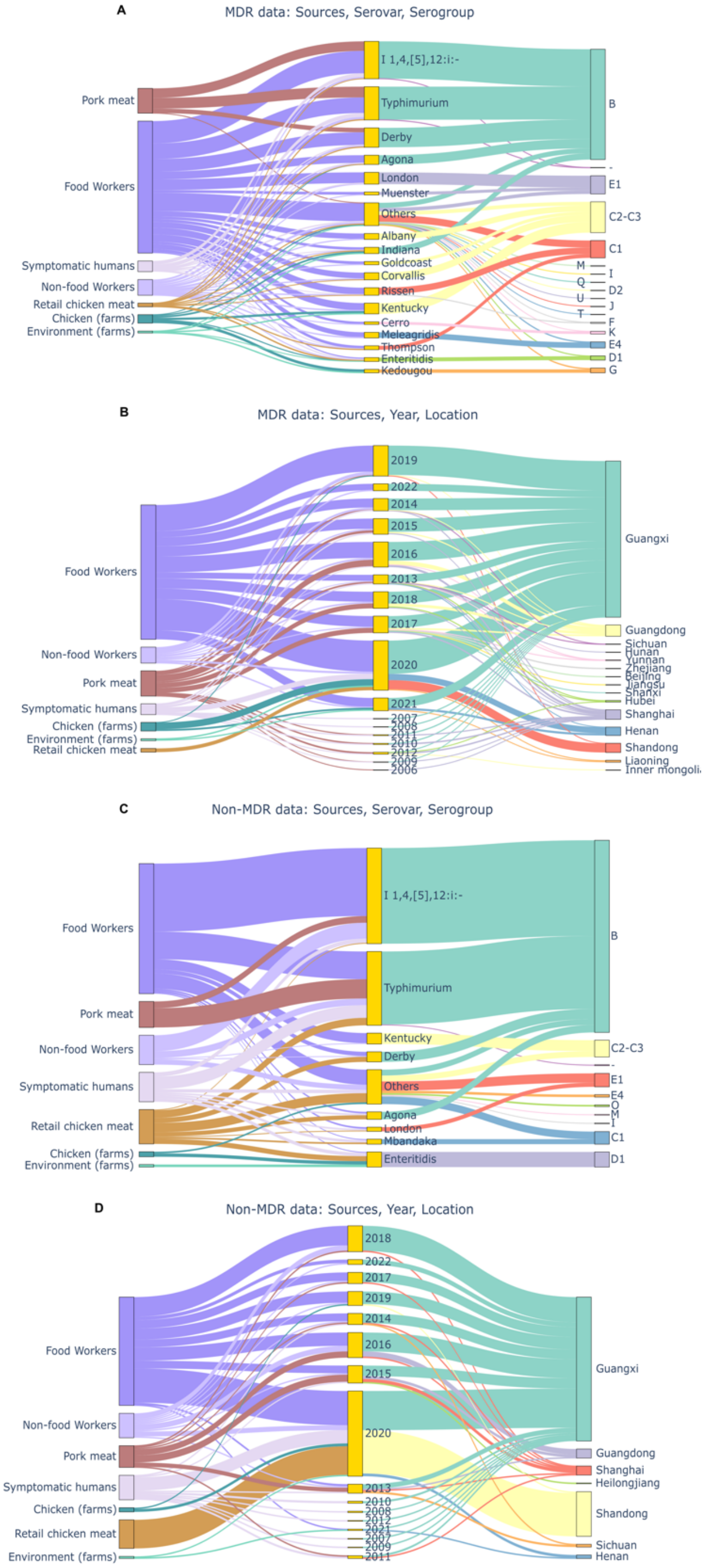


**Figure S4. Isolate information of MDR and non MDR datasets.** (A) Breakdown of MDR isolates by source type, serovar and phylogroup. (B) Breakdown of MDR isolates by source type, collection year and collection location. (C) Breakdown of non -MDR isolates by source type, serovar and phylogroup. (D) Breakdown of non-MDR isolates by source type, collection year and collection location. Source data are provided as a Source Data file.

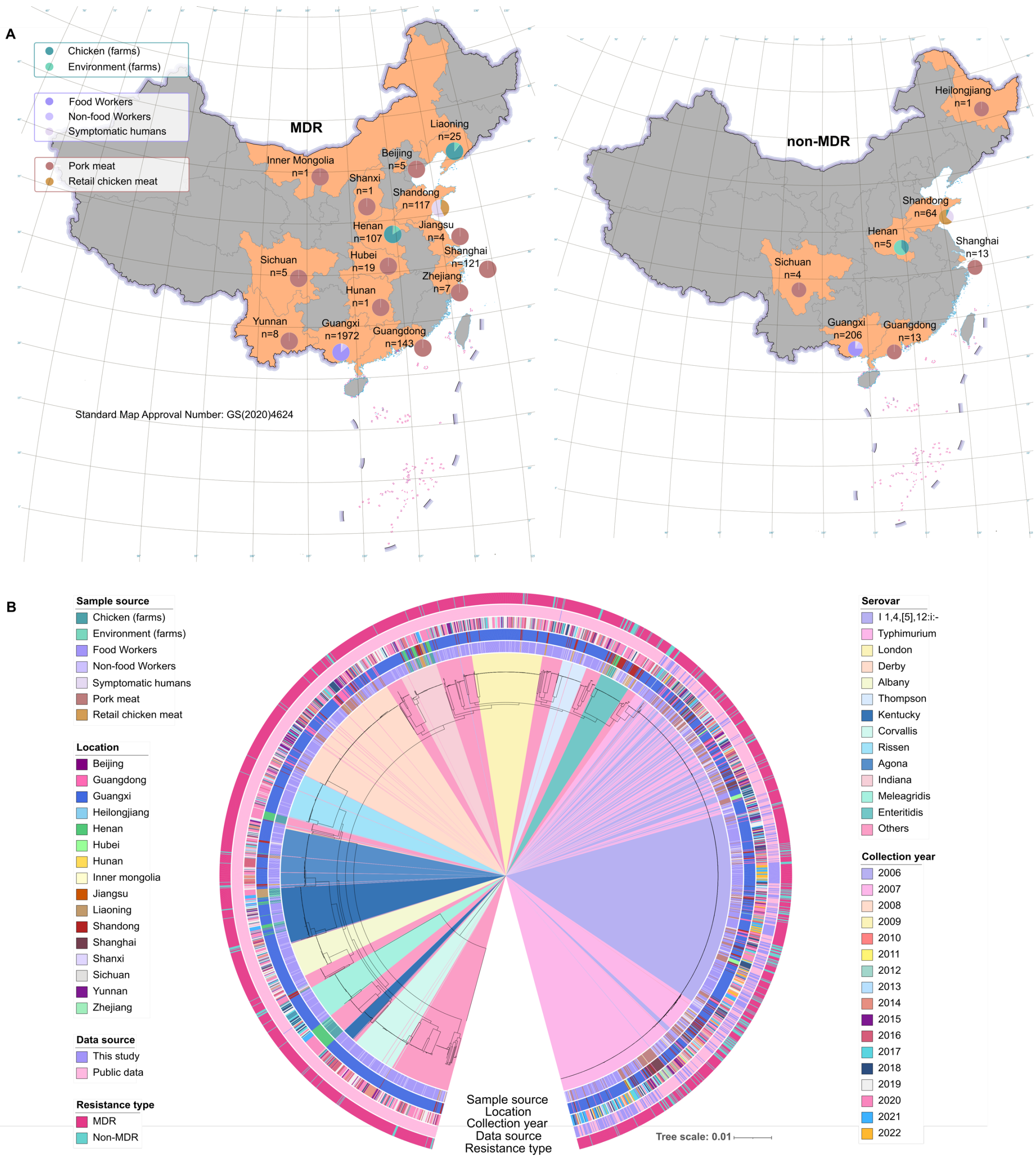


**Figure S5. Sample collection and phylogeny** (A) Maps of China (standard map approval number: GS(2020)4624 from Standard Map Service of the Ministry of Natural Resources of the People's Republic of China http://bzdt.ch.mnr.gov.cn/) , showing the provinces of China (highlighted in orange) where both MDR and non-MDR isolates throughout the food-chain were collected. Longitude and latitude lines added as overlay from standard map GS(2020)4617. For each province, the number of isolates collected is given and the split of these isolates by source type is shown as a pie chart. Source data are provided as a Source Data file.

(B) Maximum likelihood phylogenetic tree based on single nucleotide variant (SNPs) within the core genome (gene presence of 99%) of both the MDR and non-MDR isolates of *S. enterica* based on core genome of the 2843 isolates. Serovars are shown as coloured sections. Dataset, sample source, region and year of collection are shown as rings around the tree.

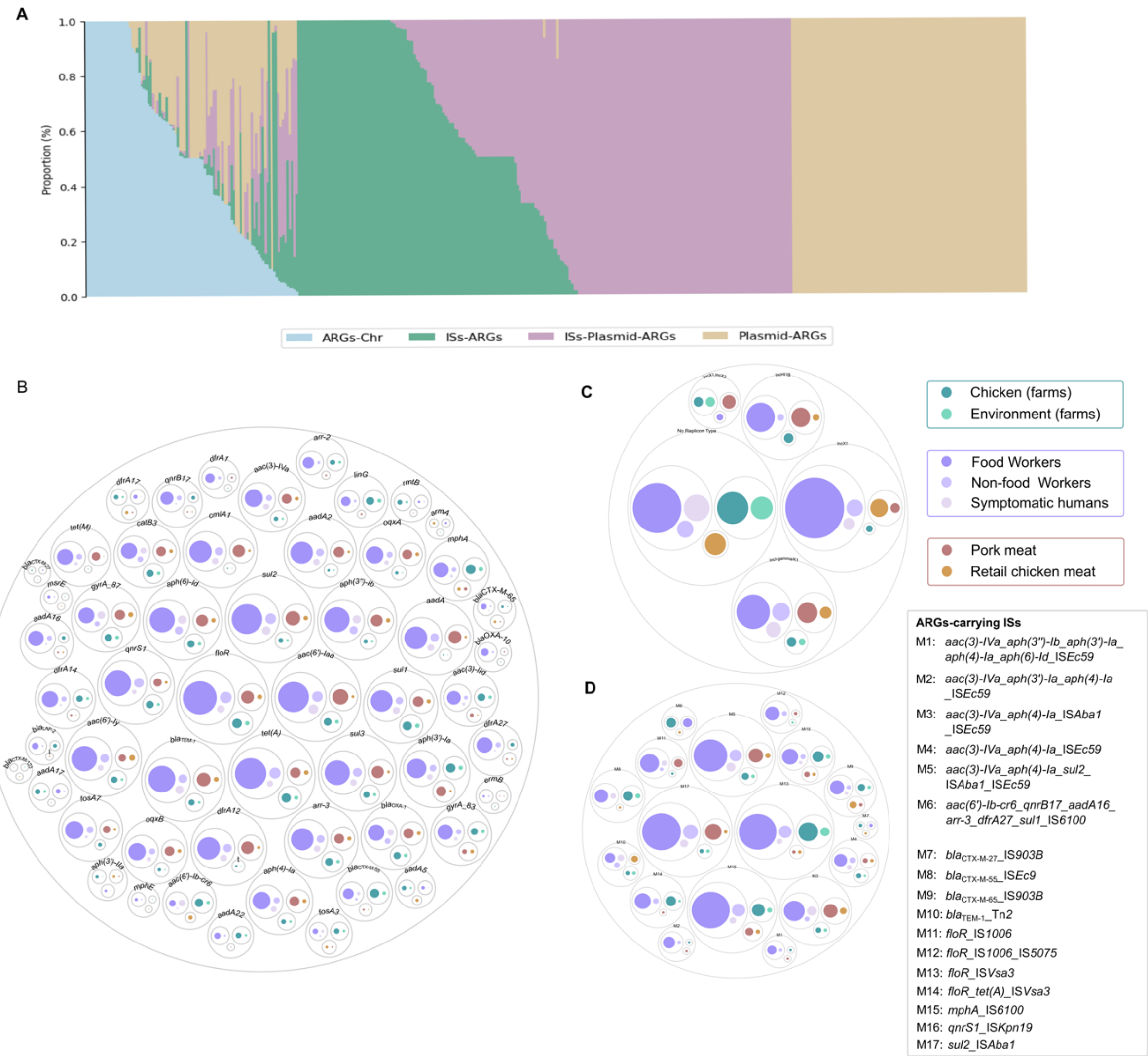


**Figure S6. AMR genomic traits within isolates and distribution across the food chain.** (A) distribution of AMR traits' locations (ARG located in the chromosome = ARGs-Chr, ARG located in the chromosome and integrated within an IS = ISs-ARGs, ARG located in the plasmid and integrated within an IS = ISs-Plasmid-ARG and ARGs present on plasmids but not within an insertion sequence = Plasmid-ARGs) across all isolates of each species. Each AMR feature is represented as a bar in the plot, with the proportion of isolates carrying the feature in each location represented by differently coloured stacked bars: ARGs-Chr (light blue), ISs-ARGs (pink), ISs-Plasmid-ARGs (green) and Plasmid-ARGs (orange); distribution through the food chain (farms: chicken and environment; humans: food workers, non-food workers, symptomatic humans; food: Pork and chicken meat), via (B) ARGs, (C) ARG-carrying plasmids and (D) ARG-carrying ISs. Only features distributed in farms, environment and humans shown in panels B-D. Source data are provided as a Source Data file.

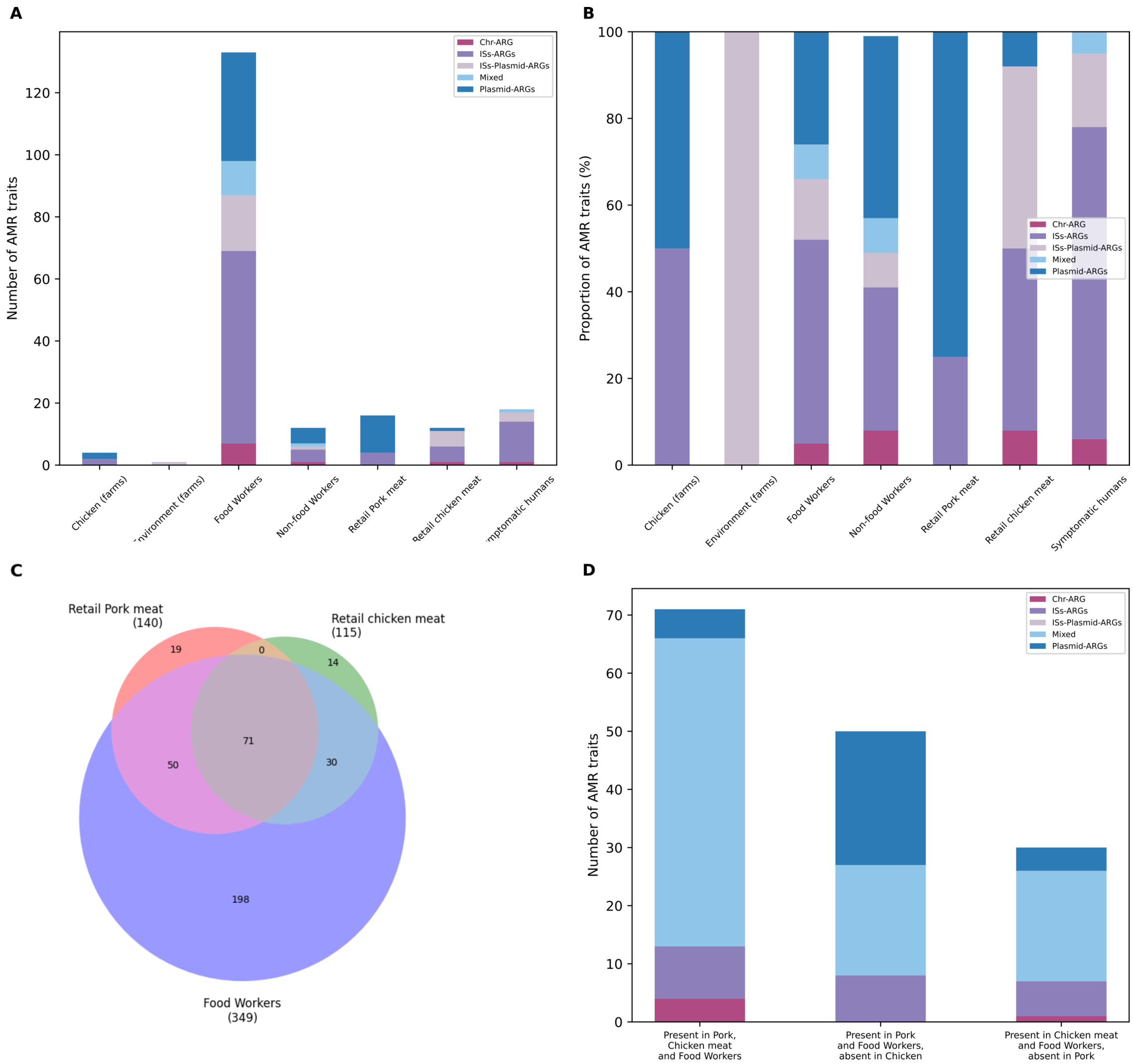


**Figure S7: Unique and shared AMR-associated genomic traits across source types** (A) The number of unique AMR-associated traits identified in each source type, separated by trait location (Chr-ARG, ISs-ARGs, ISs-Plasmid-ARGs, Plasmid-ARGs or mixed locations). (B) Percentage composition of unique AMR-associated traits within each source type, separated by trait location (Chr-ARG, ISs-ARGs, ISs-Plasmid-ARGs, Plasmid-ARGs or mixed locations). (C) Venn diagram showing the overlap of AMR-associated traits shared among isolates from food workers, retail chicken meat, and retail pork meat. (D) The distribution of AMR-associated traits, separated by trait location (Chr-ARG, ISs-ARGs, ISs-Plasmid-ARGs, Plasmid-ARGs or mixed locations), among specific source combinations: isolates from three sources (food workers, retail chicken meat, and retail pork meat); isolates from food workers and retail chicken meat but not retail pork meat; and isolates from food workers and retail pork meat but not retail chicken meat. Source data are provided as a Source Data file.

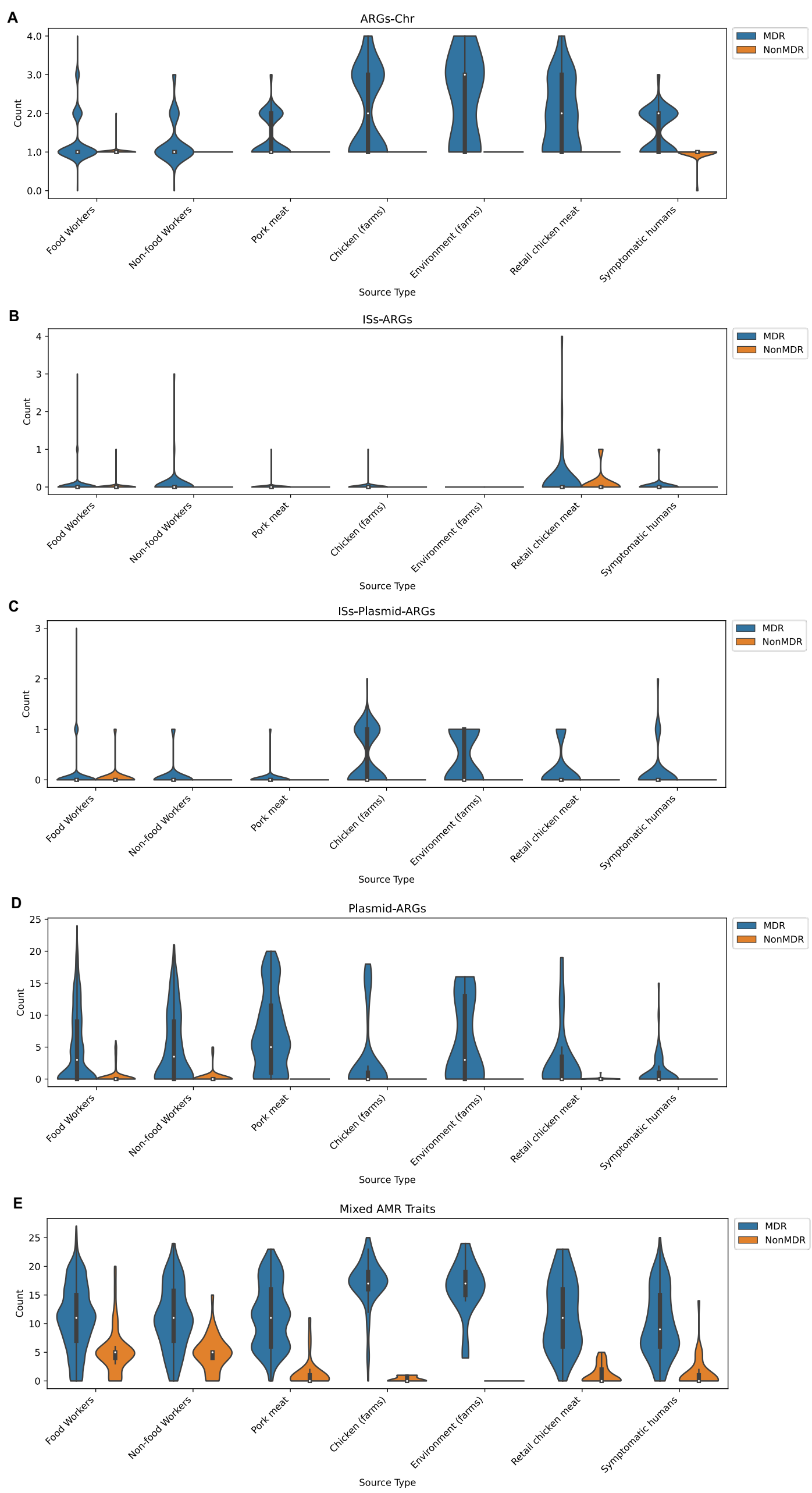


**Figure S8. Differential distribution of AMR genomic traits between MDR and non-MDR datasets** (A) Violin plot illustrating the distribution of the number of Chr-ARG traits across different source types, comparing MDR and non-MDR datasets; (B) Violin plot showing the distribution of the number of ISs-ARGs traits across different source types, comparing MDR and non-MDR datasets; (C) Violin plot depicting the distribution of the number of ISs-Plasmid-ARGs traits across different source types, (D) Violin plot showing the distribution of the number of Plasmid-ARGs traits across different source types, comparing MDR and non-MDR dataset; and (E) Violin plot showing the distribution of the number of AMR traits of mixed genomic locations across different source types, comparing MDR and non-MDR datasets. Source data are provided as a Source Data file.

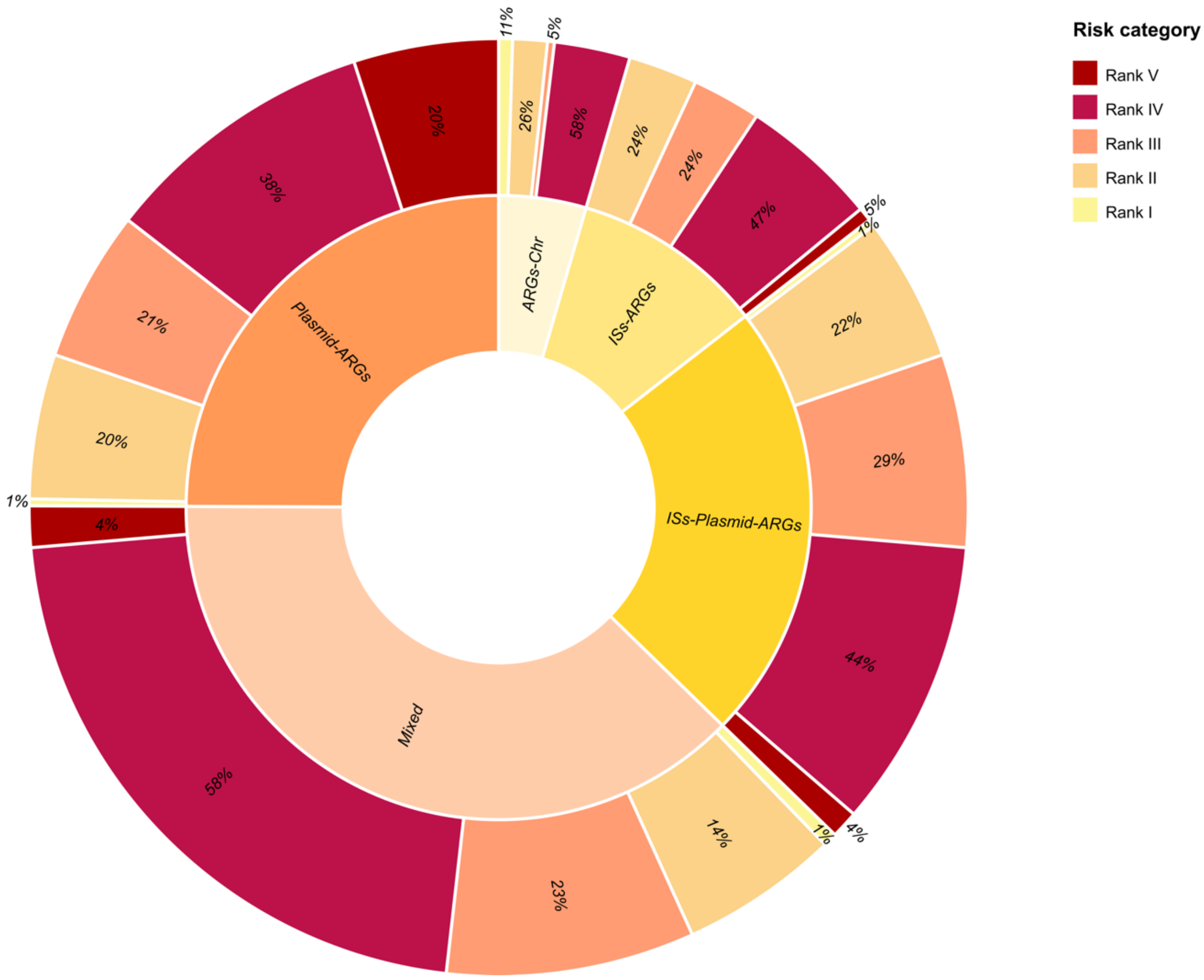


**Figure S9. Risk levels associated with AMR genomic traits disseminated throughout the food chain.** The outer ring shows the proportion of AMR traits associated with each risk level (Ranks I – V, with Rank IV and V considered high risk) while the inner ring indicates the proportion of different types of genomic traits (ARGs-Chr, ISs-ARGs, ISs-Plasmid-ARGs, Plasmid-ARGs or mixed) based on genomic location, identified within the 2536 MDR *S. enterica* isolates. Source data are provided as a Source Data file.

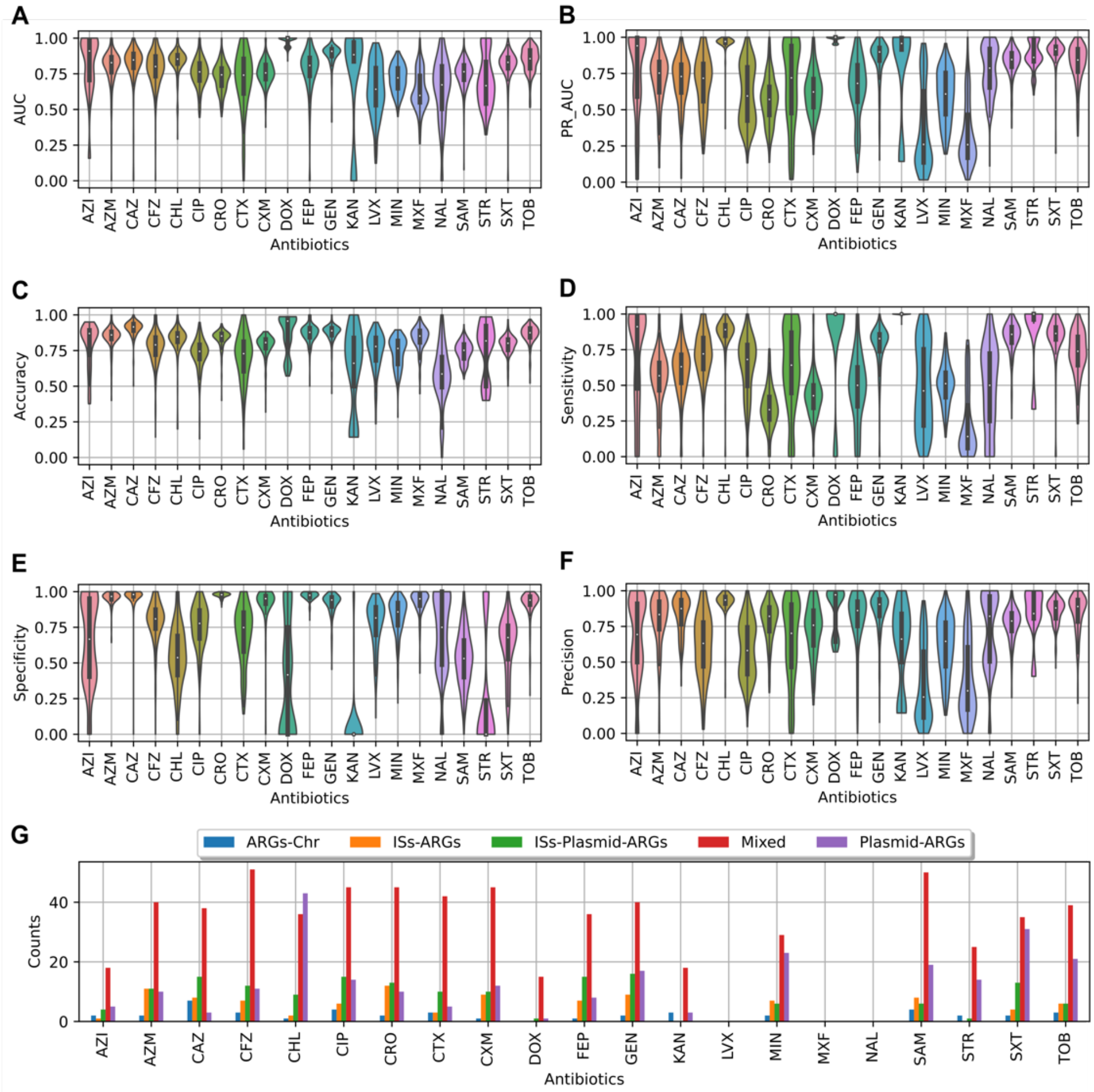


**Figure S10. Supervised machine performance metrics and feature counts from MDR isolates.** (A) – (F) Machine learning performance results for six performance indicators: (A) area under the curve AUC, (B) precision-recall AUC, (C) accuracy, (D) sensitivity, (E) Specificity, and (F) precision from 50 training runs for each antimicrobial. (G) Counts of features selected by each antibiotic model separated by feature type: ARGs-Chr (blue), ISs-ARGs (orange), ISs-Plasmid-ARGs (green), Plasmid-ARGs (red) or mixed (purple). Predictive models were generated for twenty-two different antimicrobials (X axis): azithromycin (AZI), aztreonam (AZM), ceftazidime (CAZ), cefazolin (CFZ), chloramphenicol (CHL), ciprofloxacin (CIP), ceftriaxone (CRO), cefotaxime (CTX), cefuroxime (CXM), doxycycline (DOX), cefepime (FEP), gentamicin (GEN), kanamycin (KAN), levofloxacin (LVX), minocycline (MIN), moxifloxacin (MXF), nalidixic acid (NAL), ampicillin-sulbactam (SAM), streptomycin (STR), trimethoprim-sulfamethoxazole (SXT), tobramycin (TOB), ceftiofur (XNL). Source data are provided as a Source Data file.

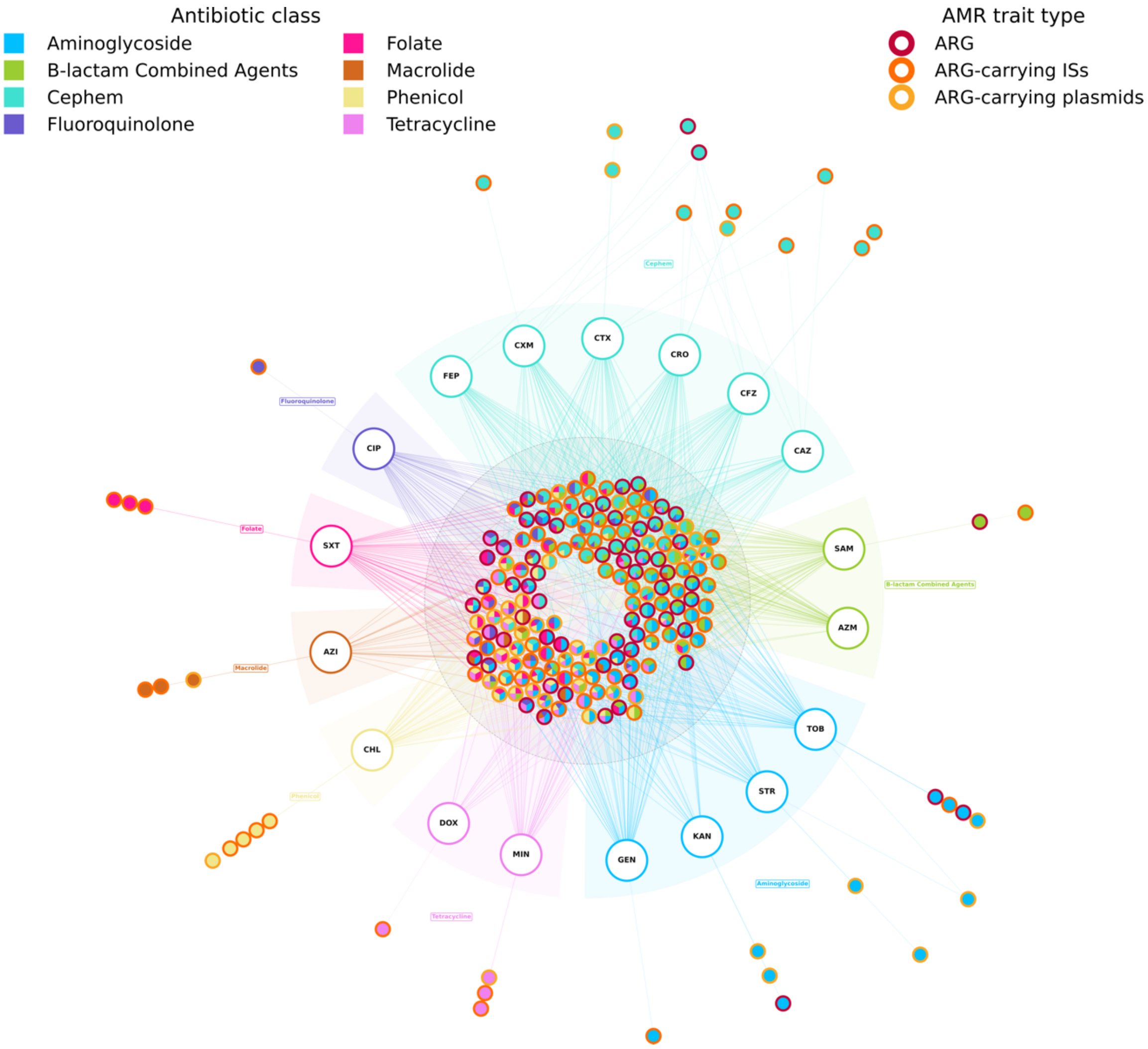


**Figure S11. Undirected graph of high risk AMR traits found by ML as predictive of resistance-susceptibility profiles to a panel of 18 antimicrobials with an AUC performance greater than 0.7 in MDR *S. enterica.*** Only rank V and rank IV AMR traits are shown. Nodes of the graph represent either the antibiotic model (indicated by three-letter antibiotic abbreviation) or the AMR trait. Trait nodes are pie charts colour-coded according antibiotic class and the border is colour-coded by the type of trait. Antibiotic nodes are colour coded by class with the colour key in the legend. Edges of graphs connect antibiotic model nodes to trait nodes and are unweighted. Node inside the circle were shared across multiple antibiotic classes; those outside the main circle were selected in only one antibiotic class. azithromycin (AZI), aztreonam (AZM), ceftazidime (CAZ), cefazolin (CFZ), chloramphenicol (CHL), ciprofloxacin (CIP), ceftriaxone (CRO), cefotaxime (CTX), cefuroxime (CXM), doxycycline (DOX), cefepime (FEP), gentamicin (GEN), kanamycin (KAN), levofloxacin (LVX), minocycline (MIN), moxifloxacin (MXF), nalidixic acid (NAL), ampicillin-sulbactam (SAM), streptomycin (STR), trimethoprim-sulfamethoxazole (SXT), tobramycin (TOB), ceftiofur (XNL). Source data are provided as a Source Data file.

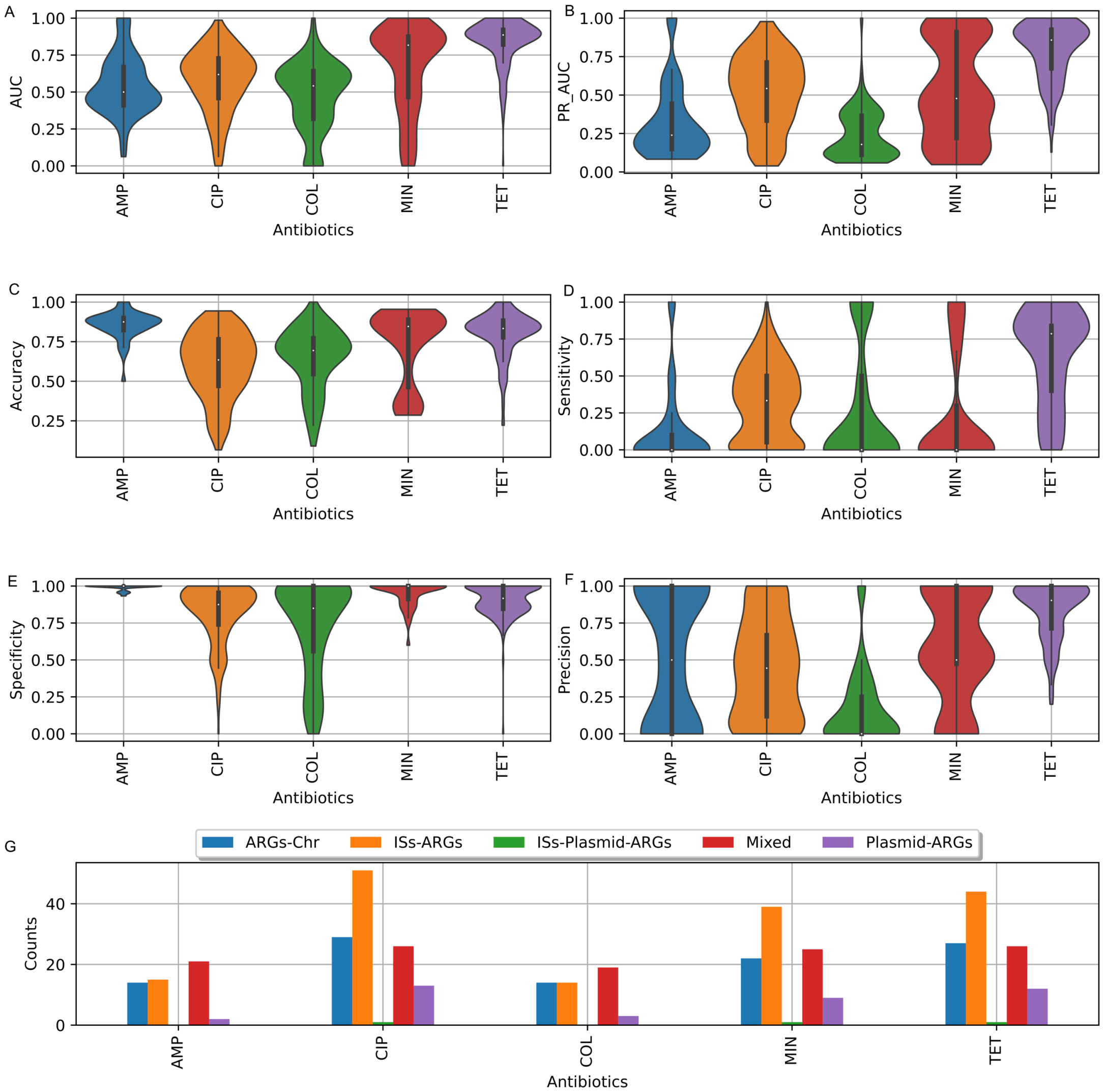


**Figure S12. Supervised machine performance metrics and feature counts from non-MDR isolates.** (A) – (F) Machine learning performance results for six performance indicators: (A) area under the curve AUC, (B) precision-recall AUC, (C) accuracy, (D) sensitivity, (E) Specificity, and (F) precision from 50 training runs for each antimicrobial. (G) Counts of features selected by each antibiotic model separated by feature type: ARGs-Chr (blue), ISs-ARGs (orange), ISs-Plasmid-ARGs (green), Plasmid-ARGs (red) or mixed (purple). Predictive models were generated for four different antimicrobials (X axis): ampicillin (AMP), colistin (COL), ciprofloxacin (CIP), minocycline (MIN), tetracycline (TET). Source data are provided as a Source Data file.

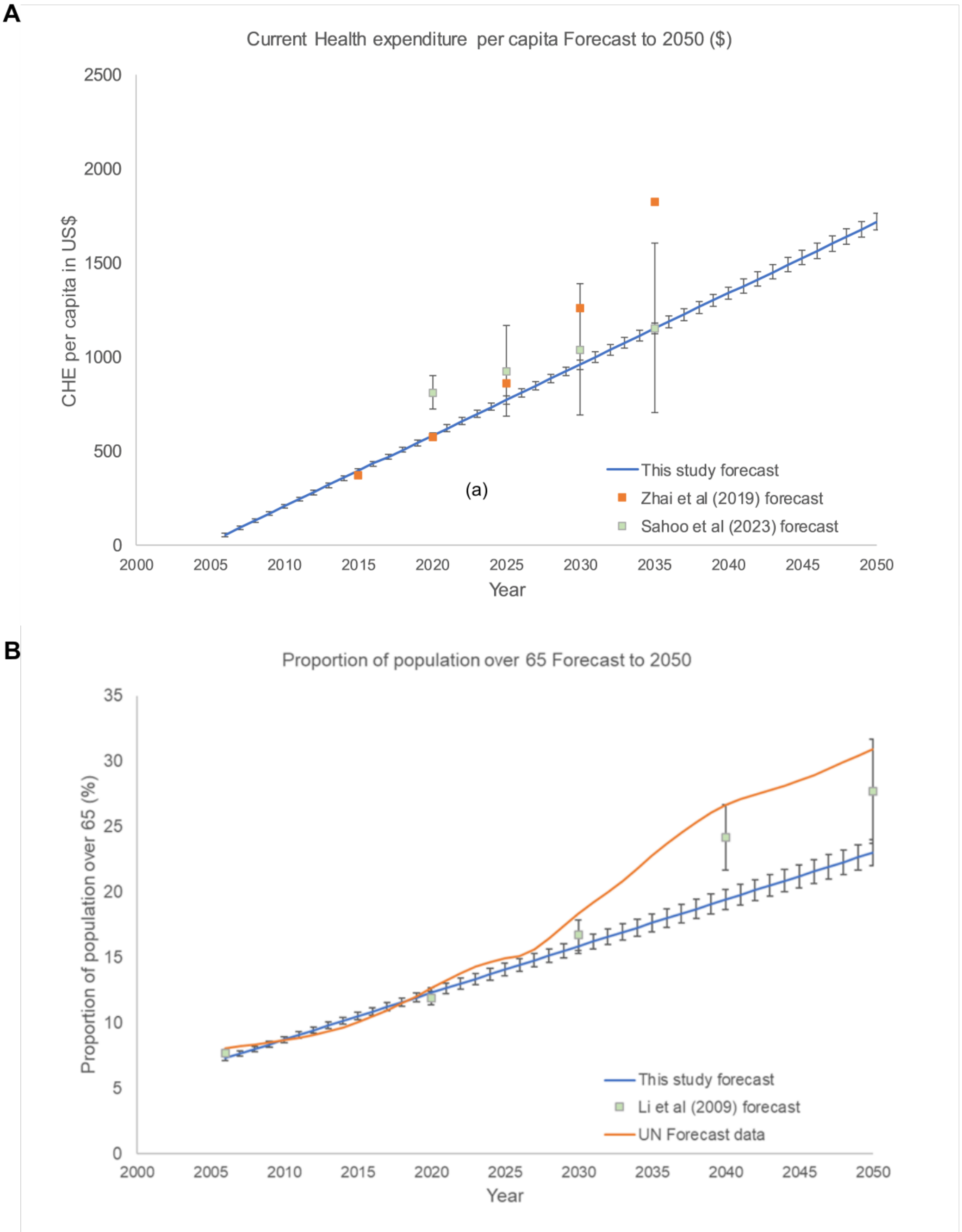


**Figure S13. Comparison of forecast indicator values to forecasts from literature.** (A) Current health expenditure forecast (blue) (mean of 10000 simulations with mean and standard deviation shown) plotted with forecasts of projected current health expenditure from Zhai, Goss [1] (orange) and Sahoo, Rout [2] (green, mean and standard deviation). (B) Proportion of population over 65 forecast (blue) ) (median of 10000 simulations with 5$^{th}$/95$^{th}$ percentiles shown) plotted against projected population over 65 from Li, Reuser [3] (green, median and 5$^{th}$/95$^{th}$ percentiles shown) and UN [4] (orange). Source data are provided as a Source Data file.

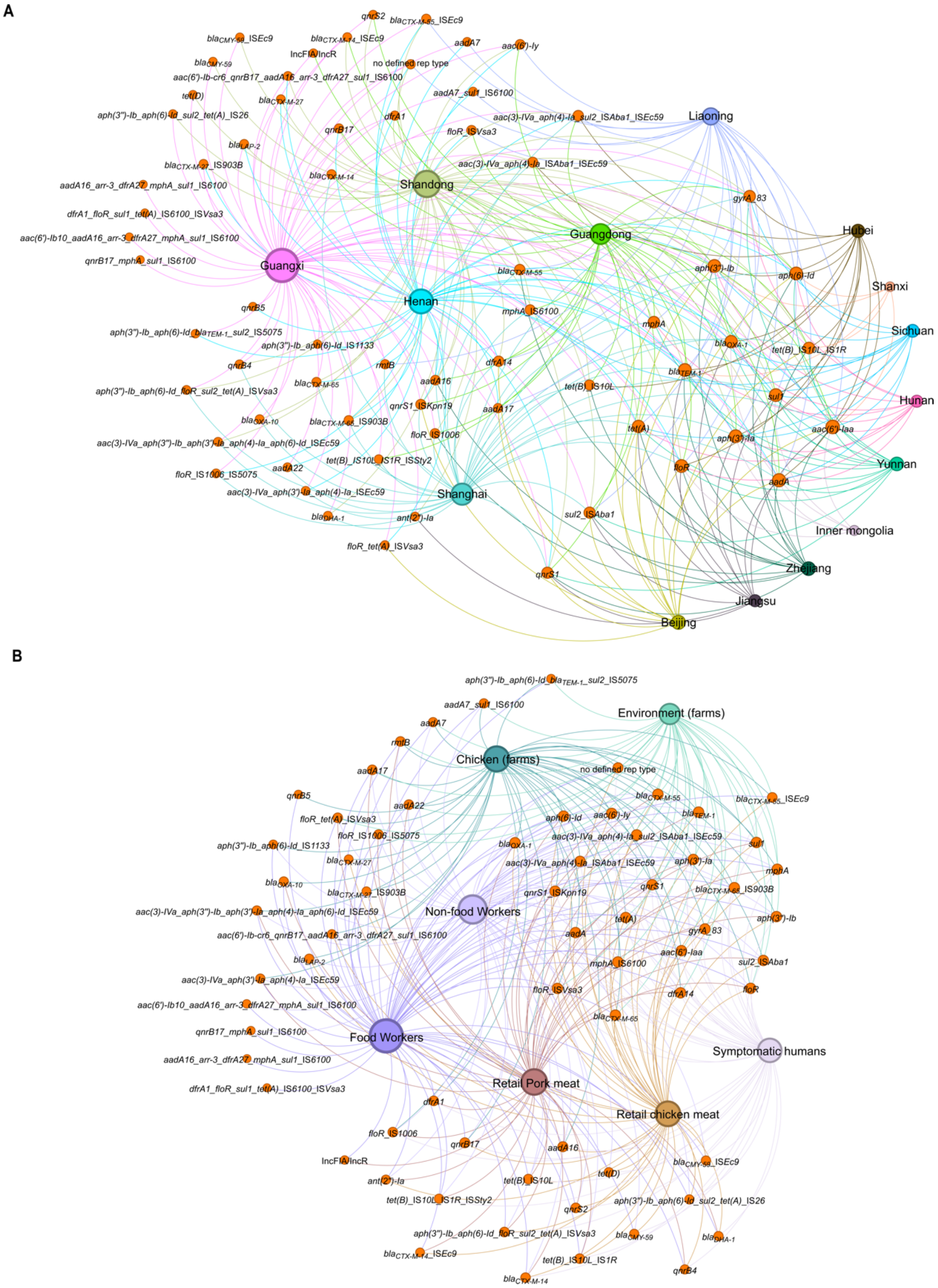


**Figure S14**. **Undirected network of high-risk AMR traits, associated with increasing or stationary indicators, connected to their associated location and source type.** (A) AMR trait nodes are color-coded based on their future trend: increasing, decreasing, stationary or a combination between them, due to the connection with different indicators. Location nodes—representing 15 provinces of China—are displayed as coloured circles, with size proportional to the number of connected features. Edges indicate the relationship between trait and location, with colour corresponding to the location. (B) AMR trait nodes are color-coded based on their future trend: increasing, decreasing, stationary or a combination between them, due to the connection with different indicators. Source type nodes—representing food workers, non-food workers, symptomatic humans, pork meat, chicken meat, chickens and their environment—are displayed as coloured circles, with size proportional to the number of connected features. Edges indicate the relationship between features and source type, with colour corresponding to the source type. Source data are provided as a Source Data file.

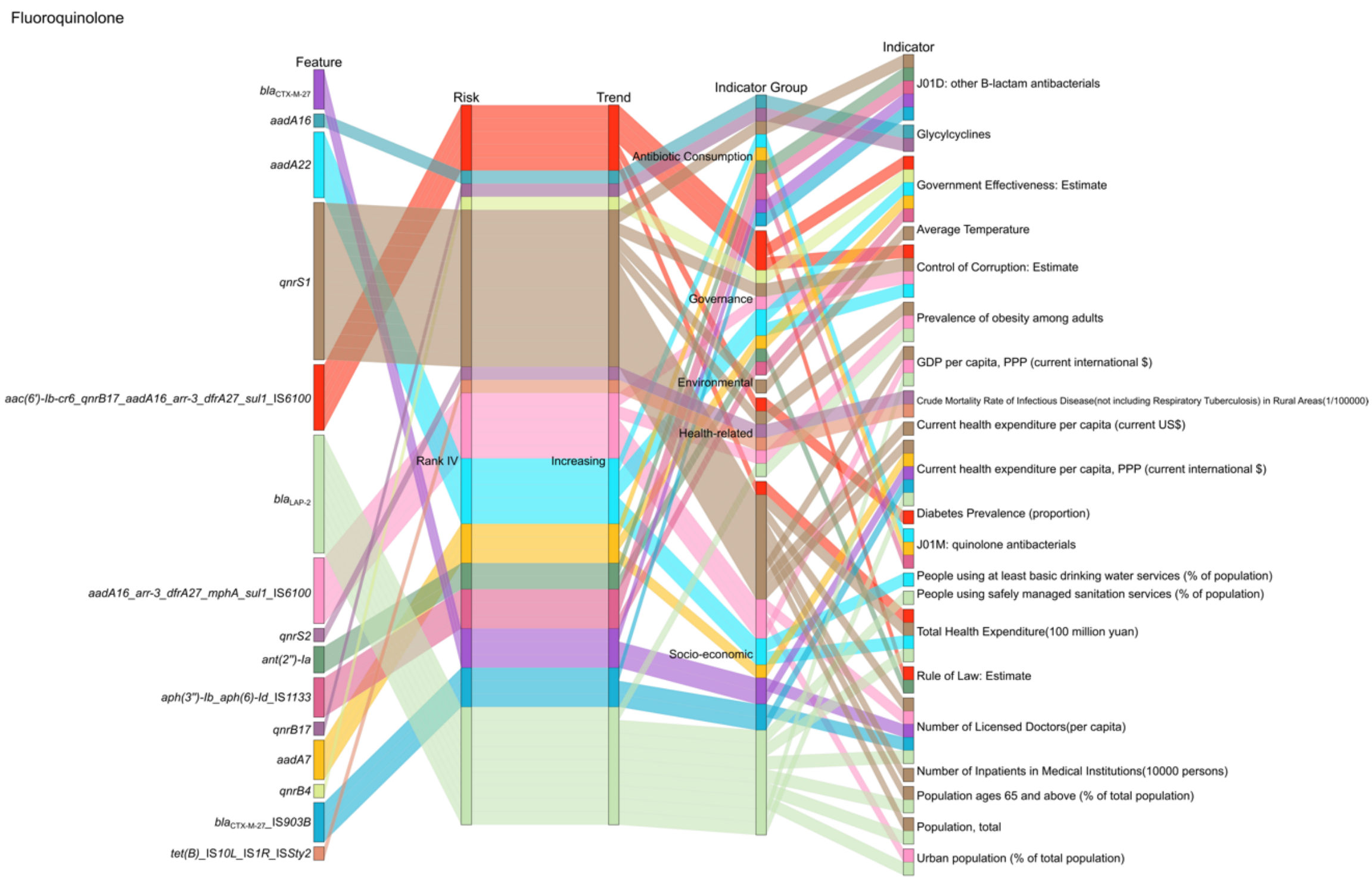


**Figure S15**. **Fluoroquinolone antibiotic model Sankey flow diagram.** This Sankey diagram illustrates the relationships between AMR genomic traits (AMR trait), their associated risk (Risk), the projected trend of each indicator (whether the indicator was forecasted to increased or remain stationary), indicator groups, and specific indicators (Indicator) within each group. Only high-risk traits associated with increasing or stationary indicators are displayed. The thickness of the links represents the number of different indicators, while the colours correspond to the associated genomic trait. Source data are provided as a Source Data file.

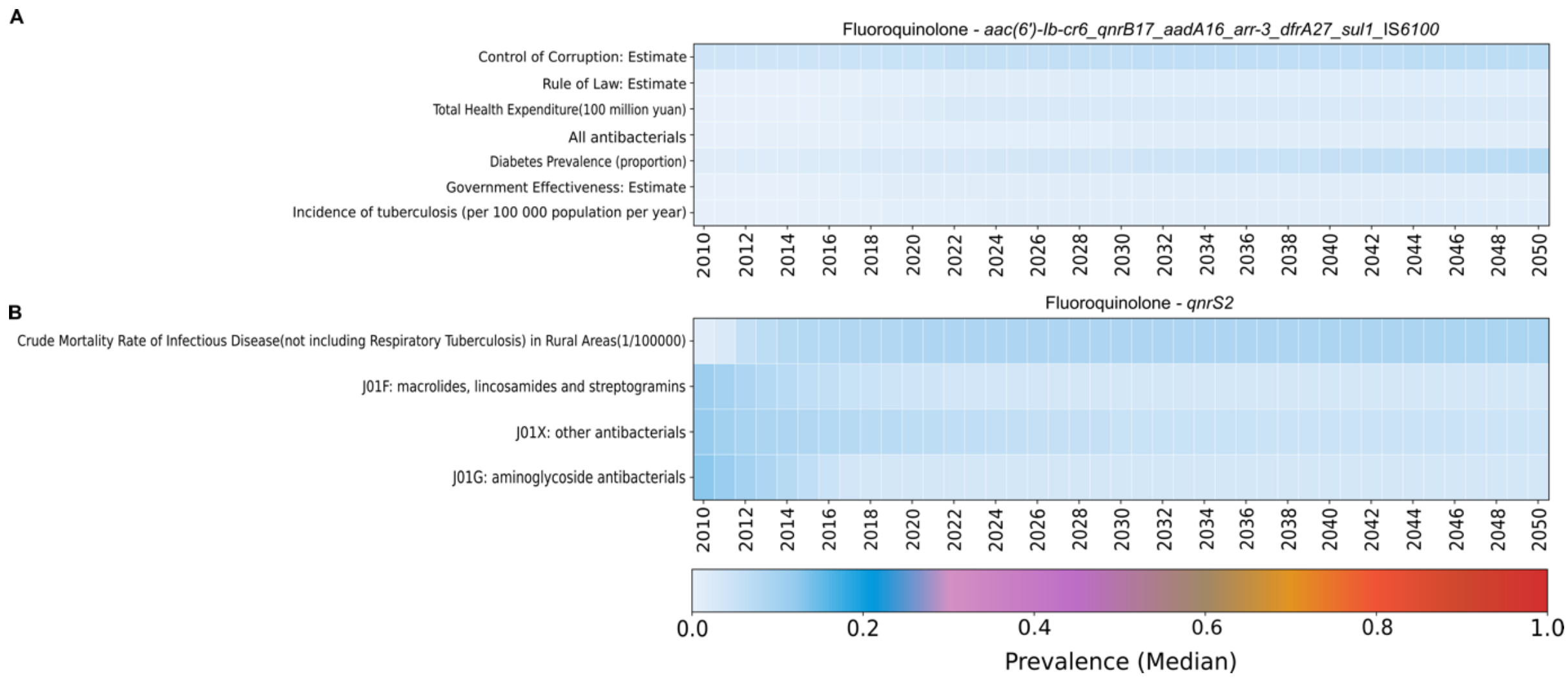


**Figure S16. Fluoroquinolone antibiotic model forecasting heatmap.** This heatmap illustrates the median value obtained by the Monte Carlo forecasts of the relative presence of the AMR traits (A) *aac(6')-Ib-cr6_qnrB17_aadA16_arr-3_dfrA27_sul1*_IS*6100*, and (B) *qnrS2* within fluoroquinolone resistant *S. enterica* to 2050 strongly correlated with increasing or stationary key determinants of health indicators. Median forecast of 10000 simulations is shown with a colour bar indicating the range of the feature prevalence rate between 0 and 1. Source data are provided as a Source Data file.

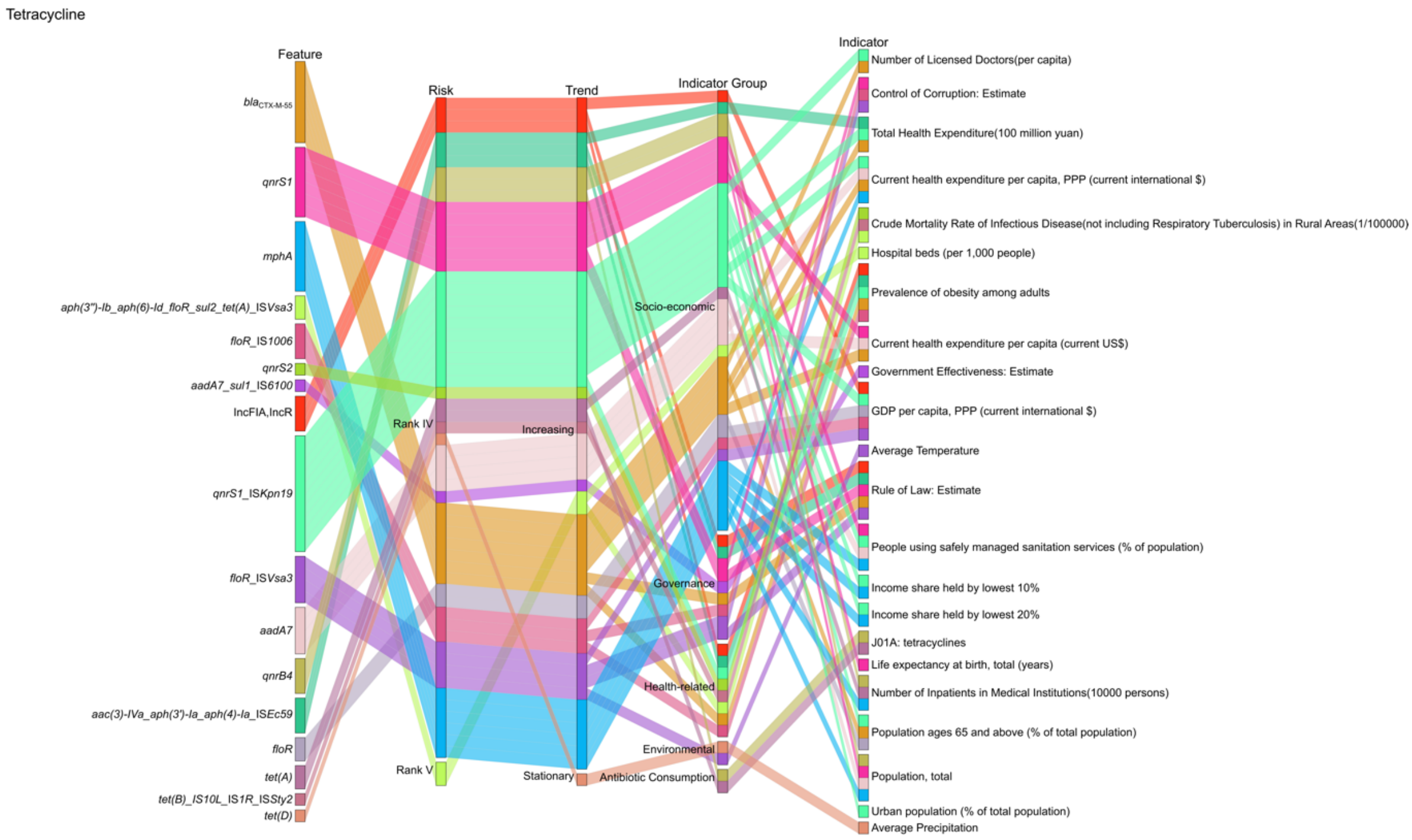


**Figure S17**. **Tetracycline antibiotic model Sankey flow diagram.** This Sankey diagram illustrates the relationships between AMR genomic traits (AMR trait), their associated risk (Risk), the projected trend of each indicator (whether the indicator was forecast to increased or remain stationary), indicator groups, and specific indicators (Indicator) within each group. Only high-risk traits associated with increasing or stationary indicators are displayed. The thickness of the links represents the number of different indicators, while the colours correspond to the associated genomic trait. Source data are provided as a Source Data file.

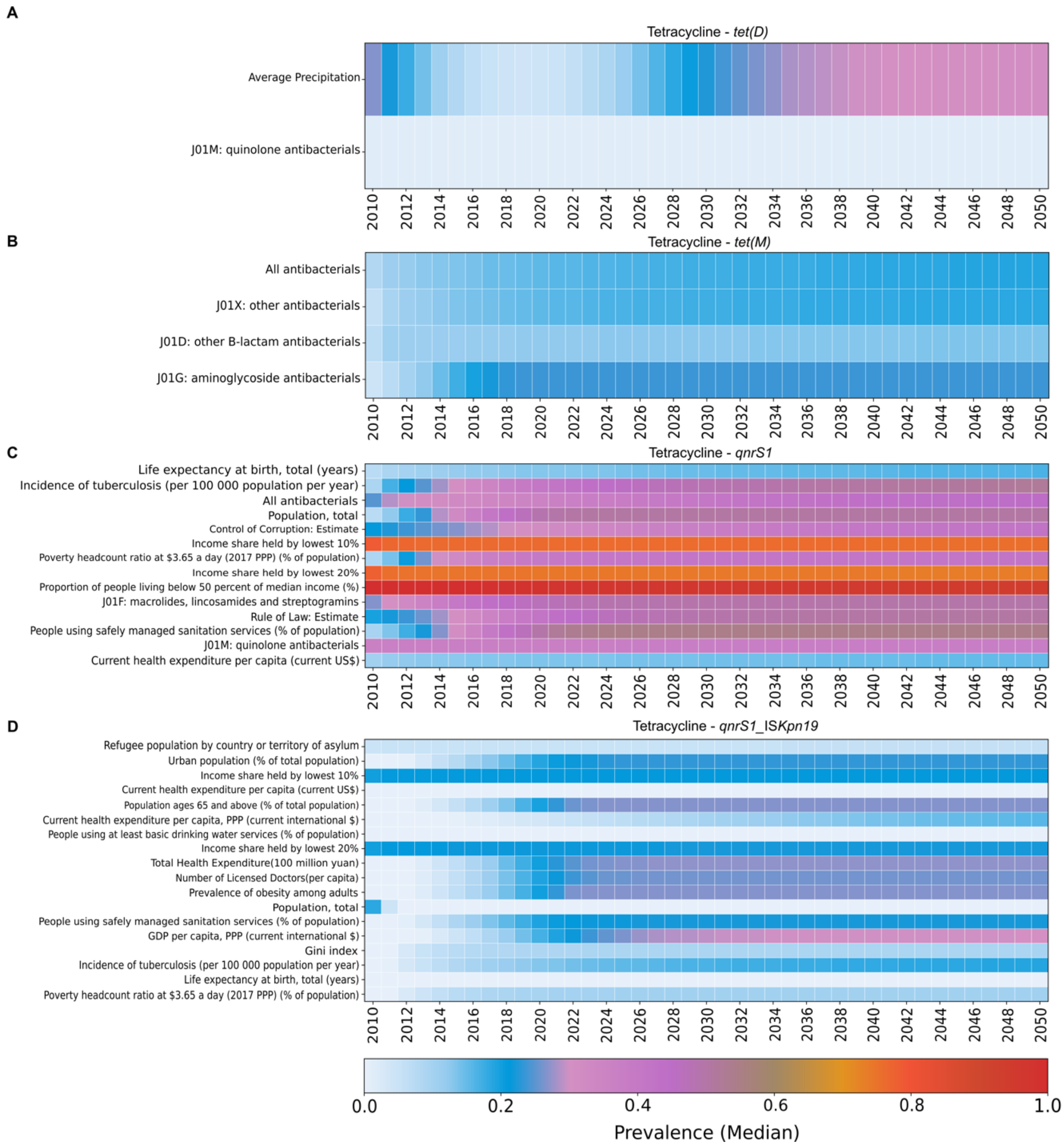


**Figure S18**. **Tetracycline antibiotic model forecasting heatmap.** This heatmap illustrates the median value obtained by the Monte Carlo forecasts of the relative presence of the AMR traits (A) *tet(D)*, (B) *tet(M)*, (C) *qnrS1* and (D) *qnrS1*_IS*Kpn19* within tetracycline resistant *S. enterica* to 2050 strongly correlated with increasing or stationary key determinants of health indicators. Median forecast of 10000 simulations is shown with a colour bar indicating the range of the feature prevalence rate between 0 and 1. Source data are provided as a Source Data file.

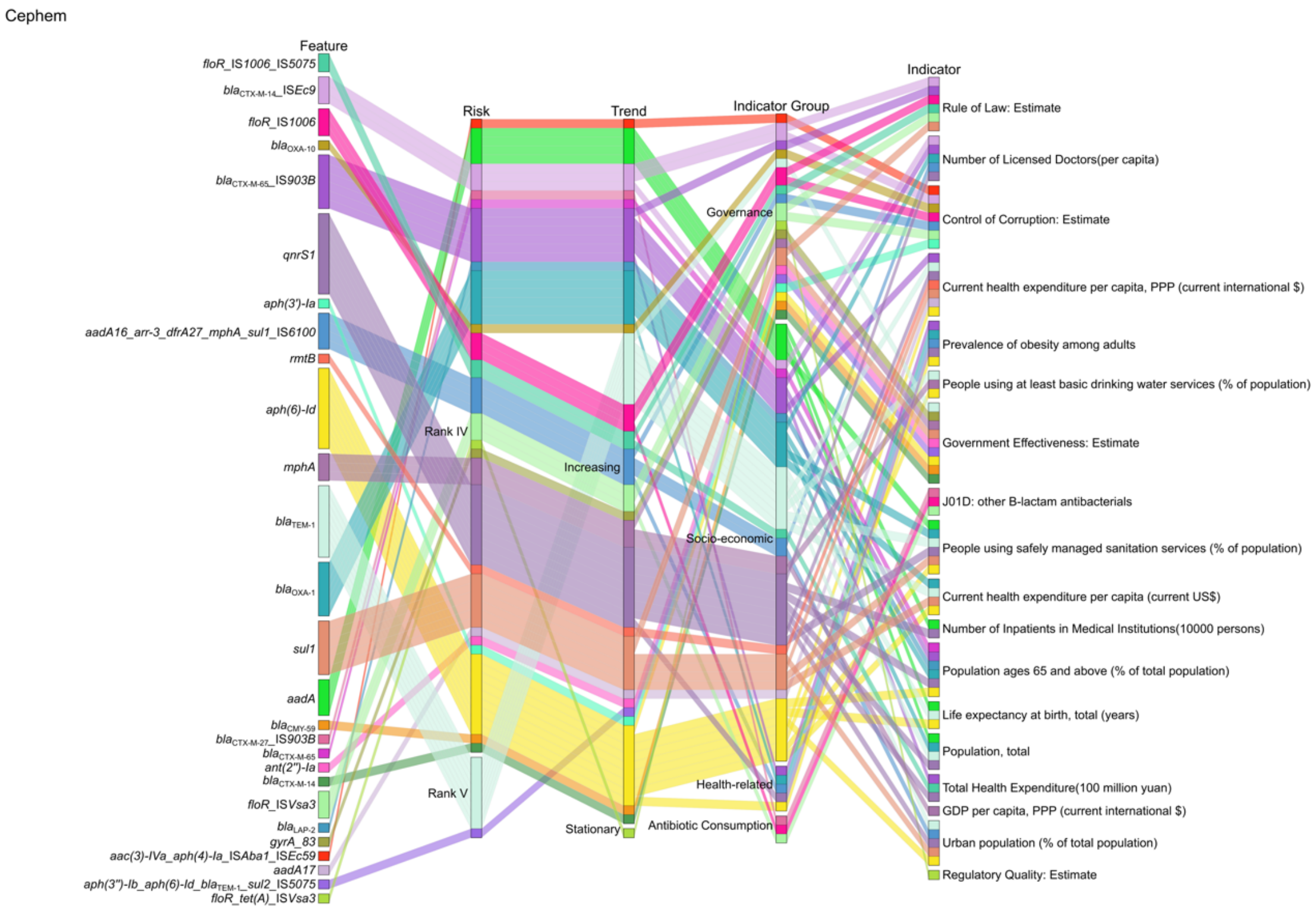


**Figure S19**. **Cephem antibiotic model Sankey flow diagram.** This Sankey diagram illustrates the relationships between AMR genomic traits (AMR trait), their associated risk (Risk), the projected trend of each indicator (whether the indicator was forecast to increased or remain stationary), indicator groups, and specific indicators (Indicator) within each group. Only high-risk AMR traits associated with increasing or stationary indicators are displayed. The thickness of the links represents the number of different indicators, while the colours correspond to the associated genomic trait. Source data are provided as a Source Data file.

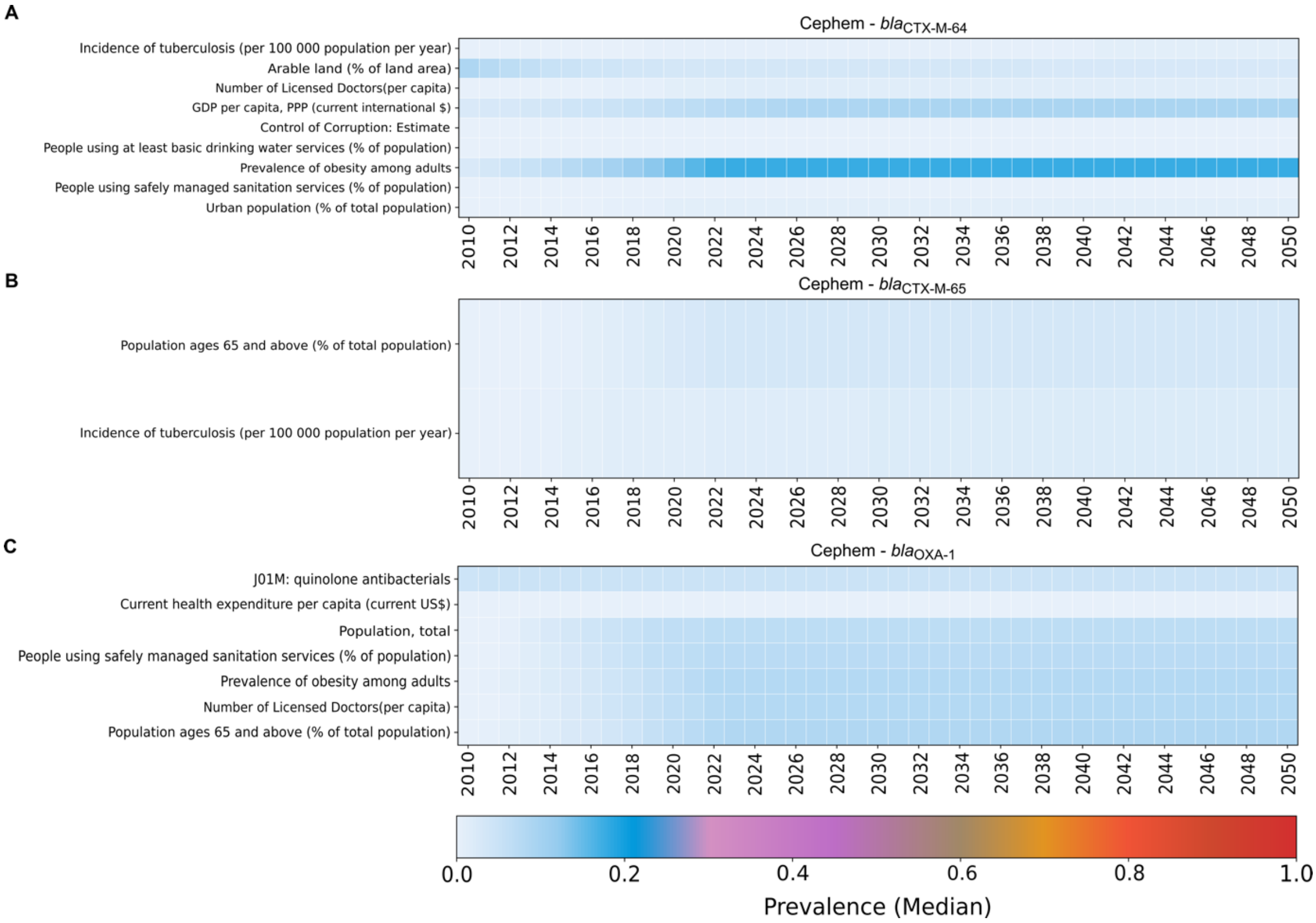


**Figure S20. Cephem antibiotic model forecasting heatmap.** This heatmap illustrates the median value obtained by the Monte Carlo forecasts of the relative presence of the AMR traits (A) $bla_{CTX-M-64}$, (B) $bla_{CTX-M-65}$ and (C) $bla_{OXA-1}$ within cephem resistant *S. enterica* to 2050 strongly correlated with increasing or stationary key determinants of health indicators. Median forecast of 10000 simulations is shown with a colour bar indicating the range of the feature prevalence rate between 0 and 1. Source data are provided as a Source Data file.

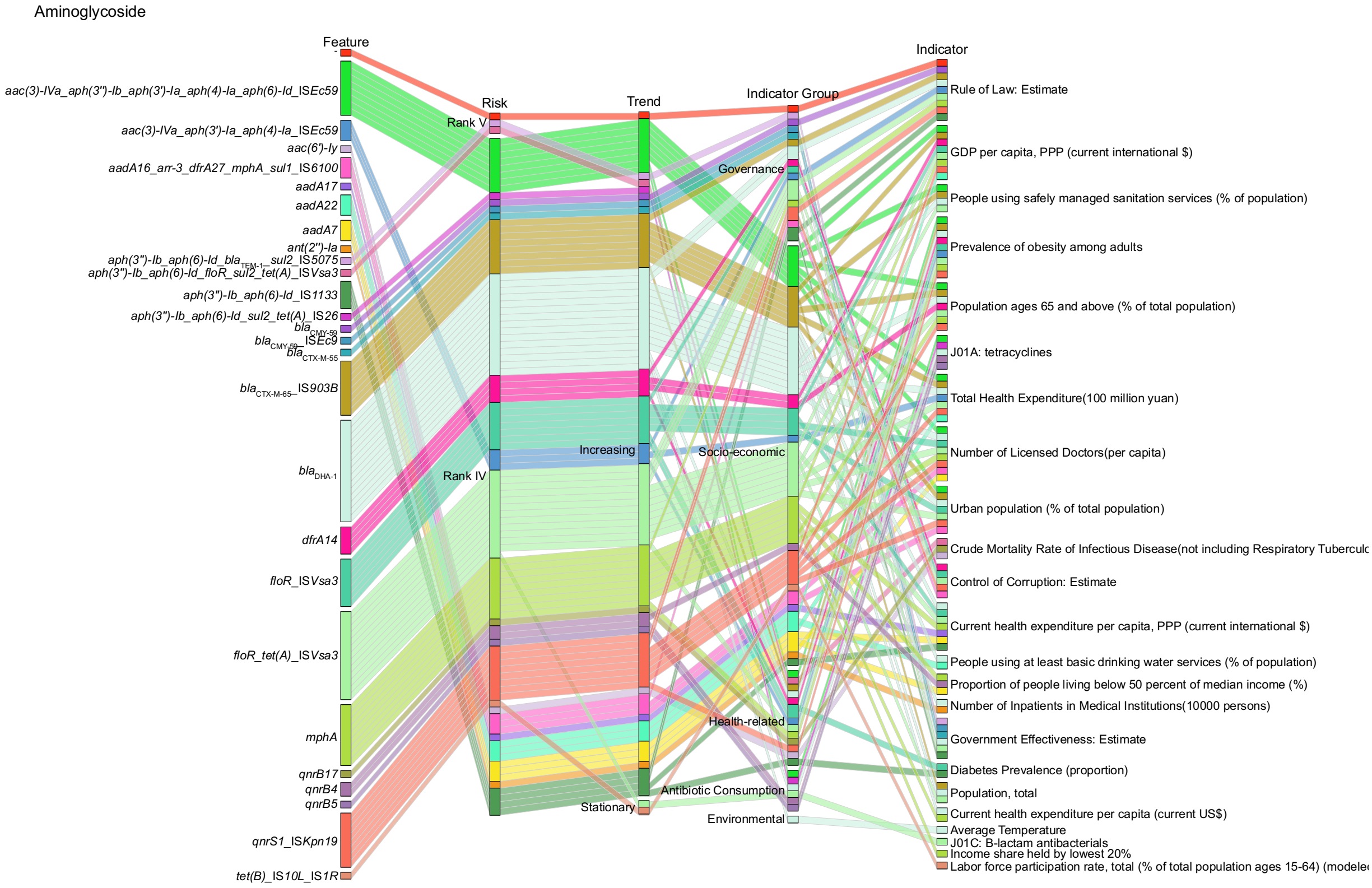


**Figure S21**. **Aminoglycoside antibiotic model Sankey flow diagram.** This Sankey diagram illustrates the relationships between AMR genomic traits (AMR trait), their associated risk (Risk), the projected trend of each indicator (whether the indicator was forecast to increase or remain stationary), indicator groups, and specific indicators (Indicator) within each group. Only high-risk traits associated with increasing or stationary indicators are displayed. The thickness of the links represents the number of different indicators, while the colours correspond to the associated genomic trait. Source data are provided as a Source Data file.

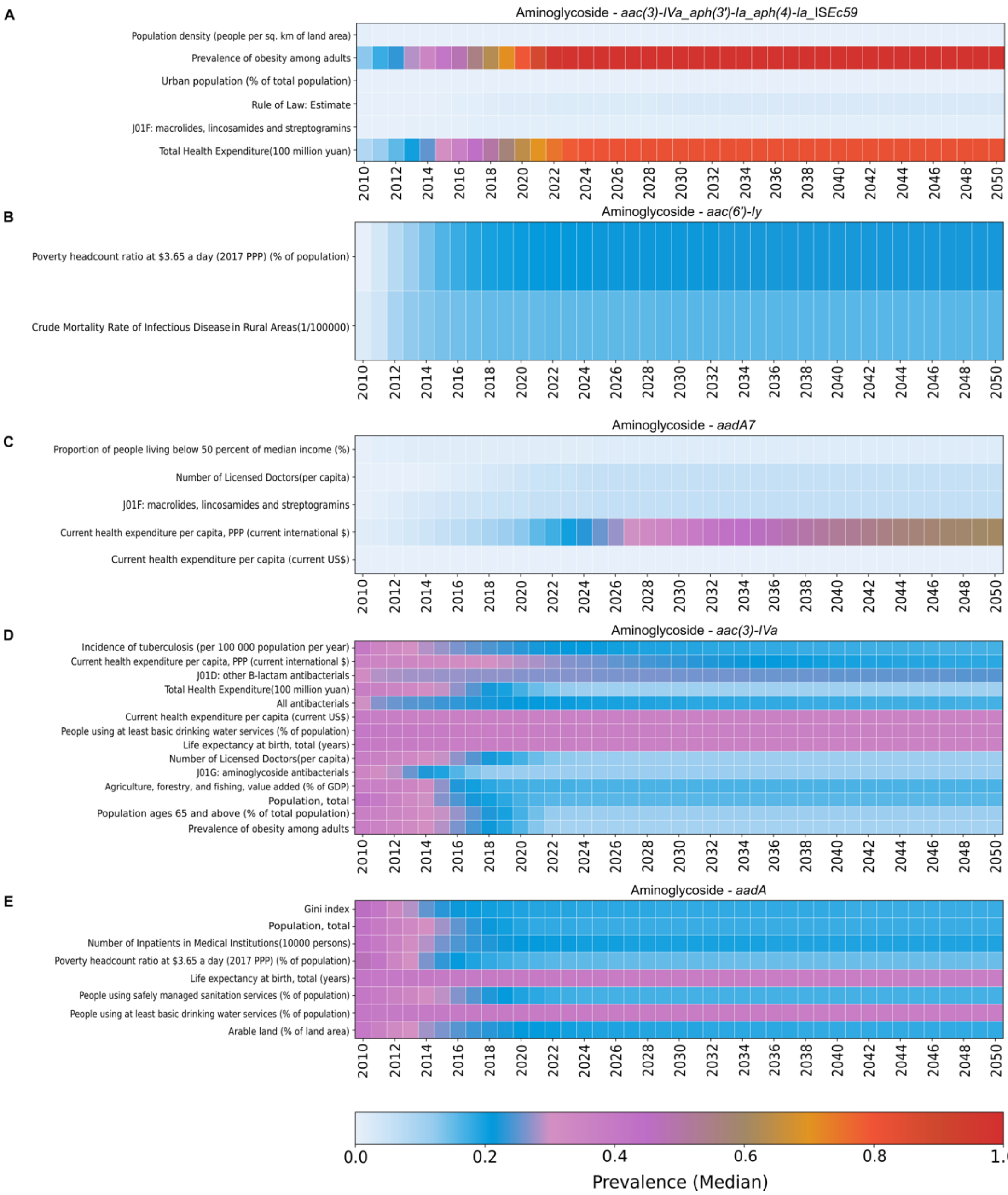


**Figure S22. Aminoglycoside antibiotic model forecasting heatmap.** This heatmap illustrates the median value obtained by the Monte Carlo forecasts of the relative presence of the AMR traits (A) *aac(3)-Iva_aph(3')-Ia_aph(4)*_IS*Ec59*, (B) *aac(6')-Iy*, (C) *aadA7,* (D) *aac(3)-IVa* and (E) *aadA* within aminoglycoside resistant *S. enterica* to 2050 strongly correlated with increasing or stationary key determinants of health indicators. Median forecast of 10000 simulations is shown with a colour bar indicating the range of the feature prevalence rate between 0 and 1. Source data are provided as a Source Data file.

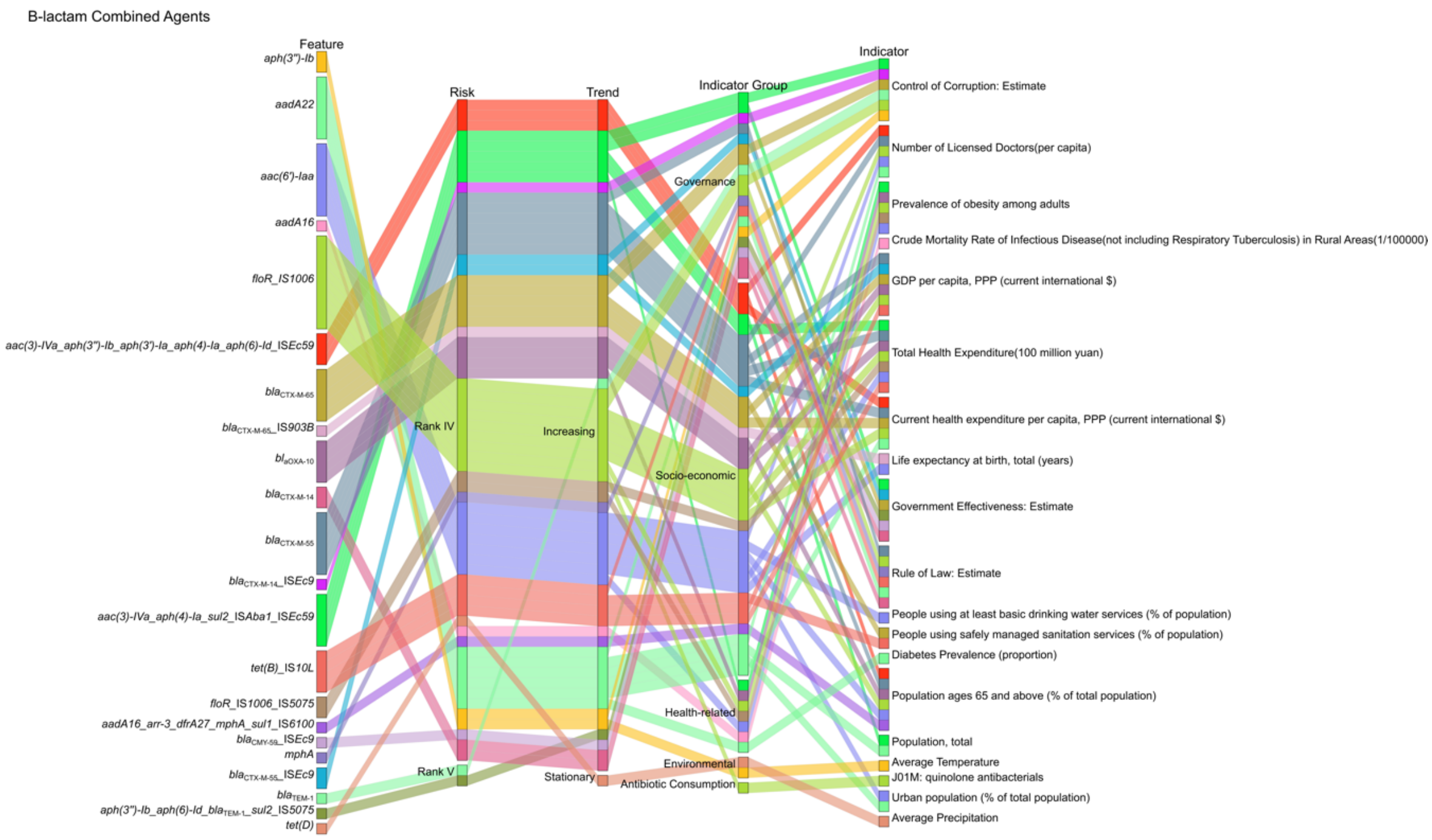


**Figure S23**. **β-lactam Combined Agents antibiotic model Sankey flow diagram.** This Sankey diagram illustrates the relationships between AMR genomic traits (AMR trait), their associated risk (Risk), the projected trend of each indicator (whether the indicator was forecast to increase or remain stationary), indicator groups, and specific indicators (Indicator) within each group. Only high-risk traits associated with increasing or stationary indicators are displayed. The thickness of the links represents the number of different indicators, while the colours correspond to the associated genomic trait. Source data are provided as a Source Data file.

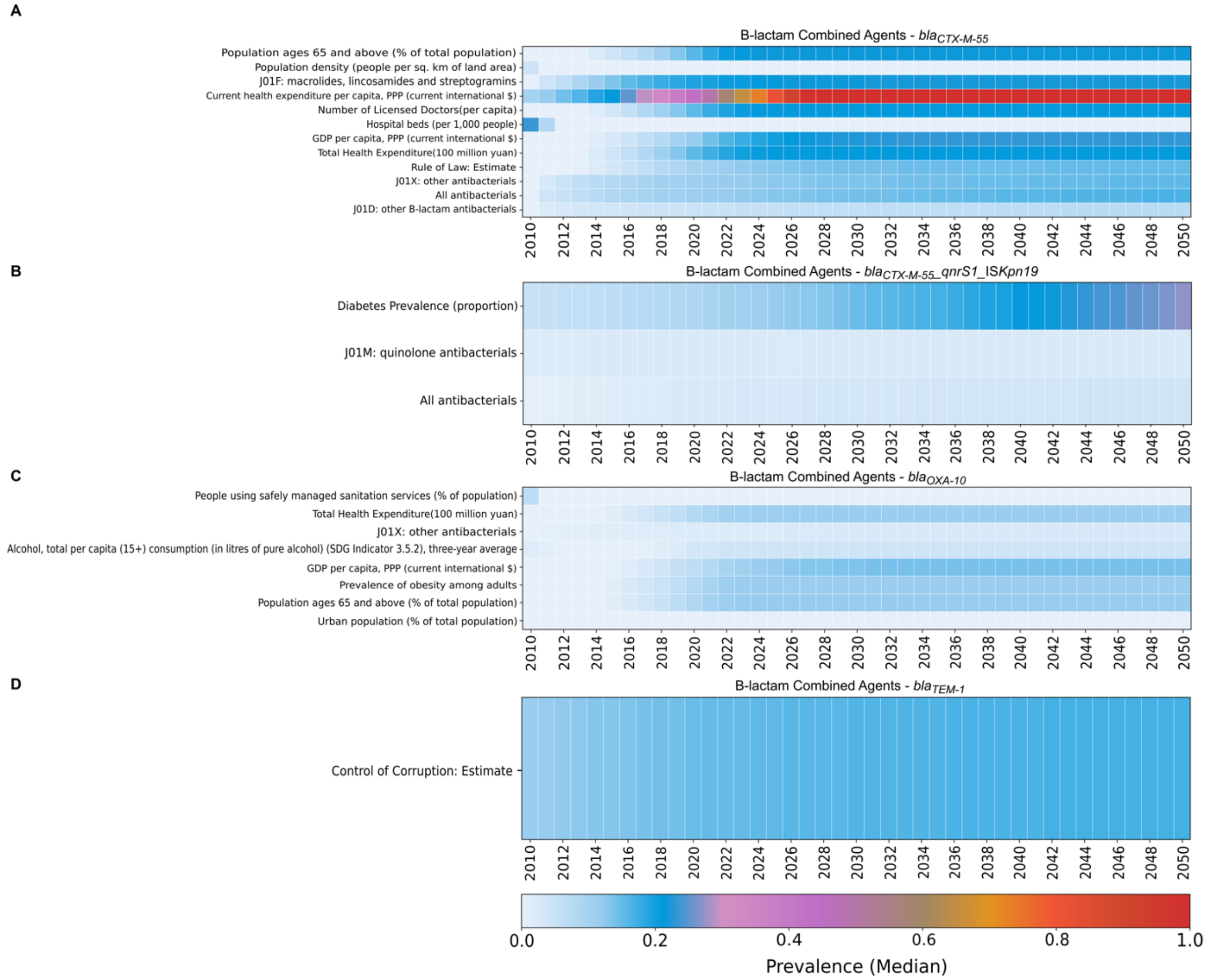


**Figure S24. β-lactam Combined Agents antibiotic model forecasting heatmap.** This heatmap illustrates the median value obtained by the Monte Carlo forecasts of the relative presence of the AMR traits (A) *bla*$_{CTX-M-55}$, (B) *bla*$_{CTX-M-55}$_*qnrS1*_IS*Kpn19*, (C) *bla*$_{OXA-10}$ and (D) *bla*$_{TEM-1}$ within β-lactam Combined Agents resistant *S. enterica* to 2050 strongly correlated with increasing or stationary key determinants of health indicators. Median forecast of 10000 simulations is shown with a colour bar indicating the range of the feature prevalence rate between 0 and 1. Source data are provided as a Source Data file.

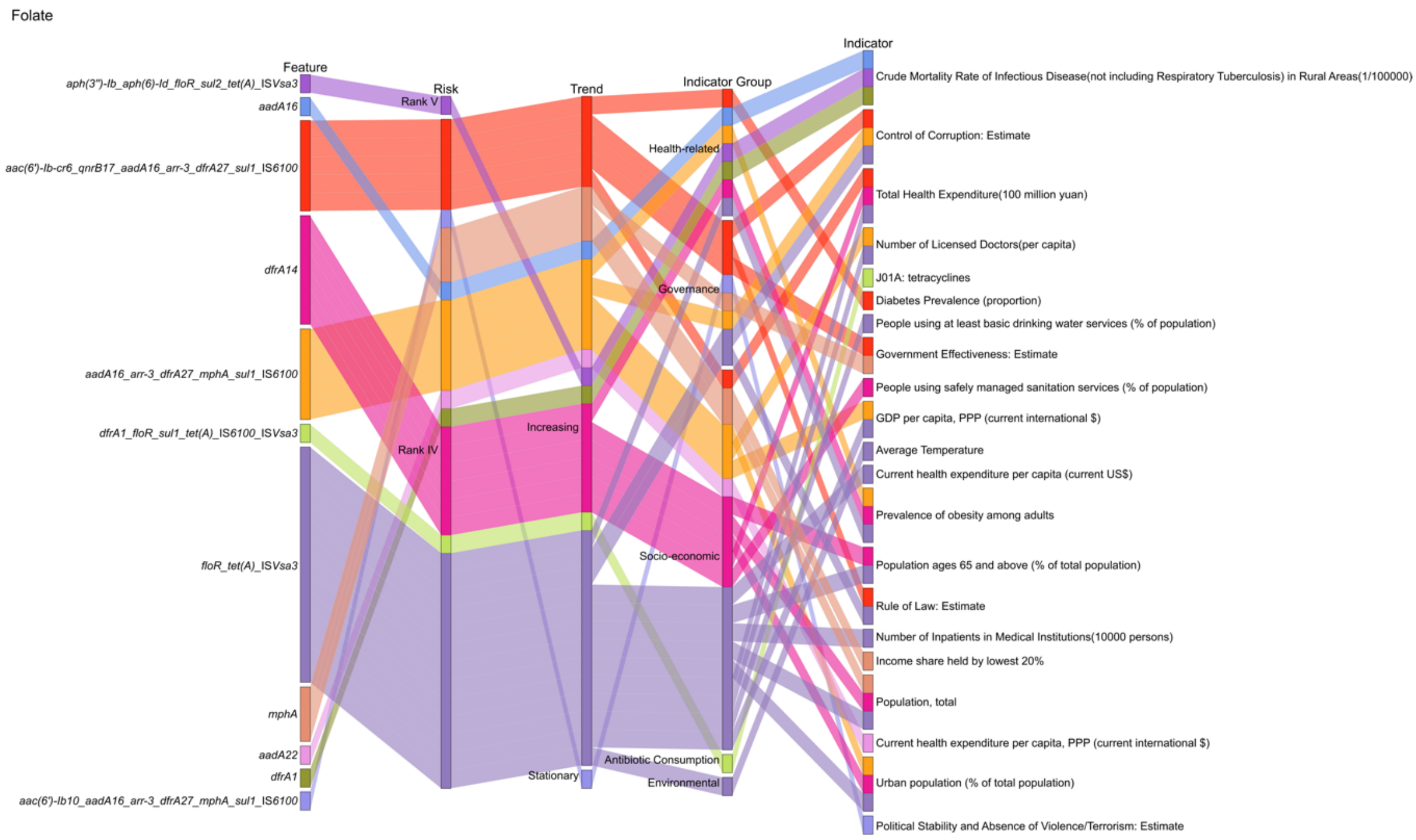


**Figure S25**. **Folate antibiotic model Sankey flow diagram.** This Sankey diagram illustrates the relationships between AMR genomic traits (AMR trait), their associated risk (Risk), the projected trend of each indicator (whether the indicator was forecast to increase or remain stationary), indicator groups, and specific indicators (Indicator) within each group. Only high-risk traits associated with increasing or stationary indicators are displayed. The thickness of the links represents the number of different indicators, while the colours correspond to the associated genomic trait. Source data are provided as a Source Data file.

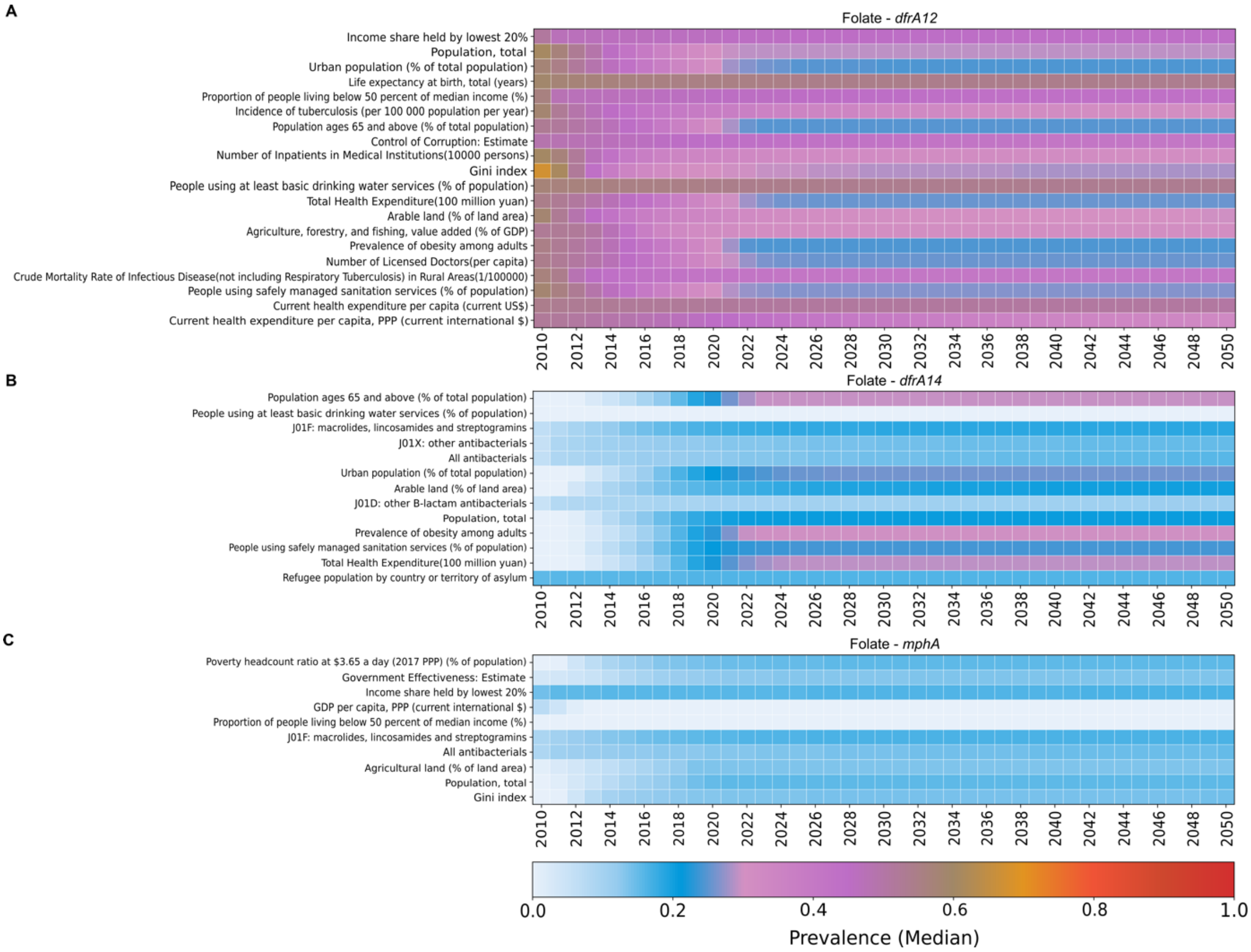


**Figure S26. Folate antibiotic model forecasting heatmap.** This heatmap illustrates the median value obtained by the Monte Carlo forecasts of the relative presence of the AMR trait (A) *dfrA12*, (B) *dfrA14*, and (C) *mphA* within folate resistant *S. enterica* to 2050 strongly correlated with increasing or stationary key determinants of health indicators. Median forecast of 10000 simulations is shown with a colour bar indicating the range of the feature prevalence rate between 0 and 1. Source data are provided as a Source Data file.

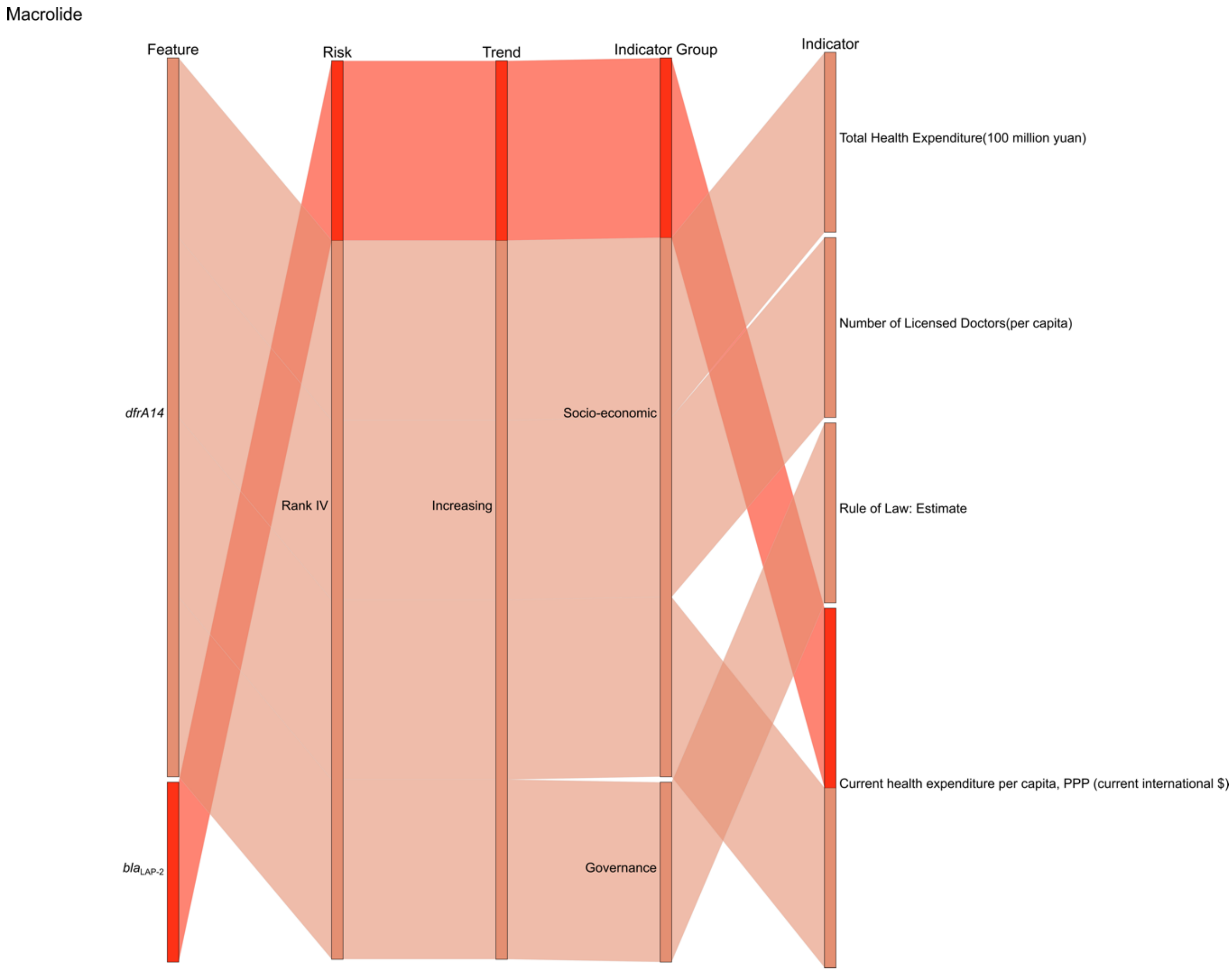


**Figure S27**. **Macrolide antibiotic model Sankey flow diagram.** This Sankey diagram illustrates the relationships between AMR genomic traits (AMR trait), their associated risk (Risk), the projected trend of each indicator (whether the indicator was forecast to increase or remain stationary), indicator groups, and specific indicators (Indicator) within each group. Only high-risk traits associated with increasing or stationary indicators are displayed. The thickness of the links represents the number of different indicators, while the colours correspond to the associated genomic trait. Source data are provided as a Source Data file.

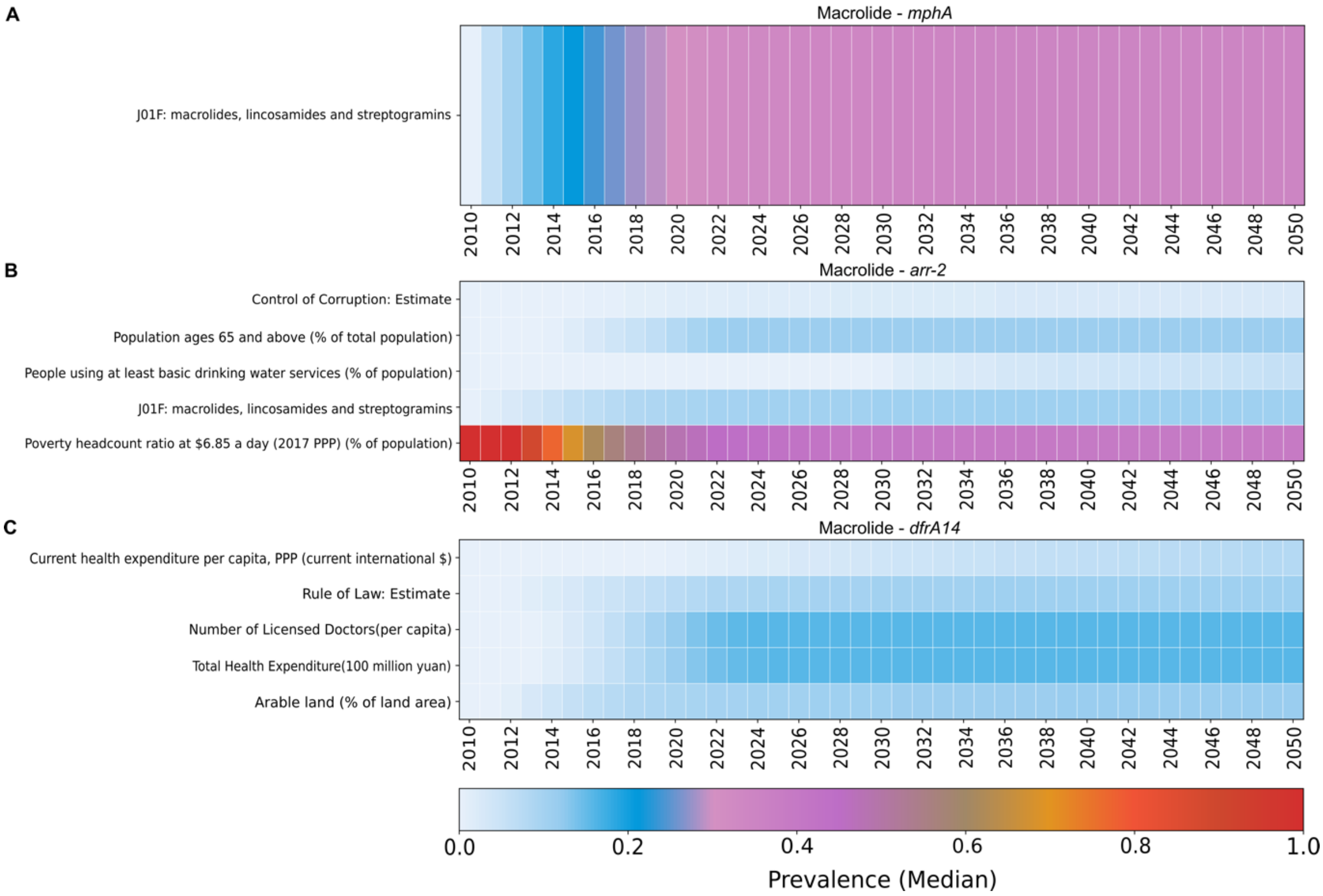


**Figure S28. Macrolide antibiotic model forecasting heatmap.** This heatmap illustrates the median value obtained by the Monte Carlo forecasts of the relative presence of the AMR traits (A) *mphA,* (B) *arr-3* and (C) *dfrA14*, within macrolide resistant *S. enterica* to 2050 strongly correlated with increasing or stationary key determinants of health indicators. Median forecast of 10000 simulations is shown with a colour bar indicating the range of the feature prevalence rate between 0 and 1. Source data are provided as a Source Data file.

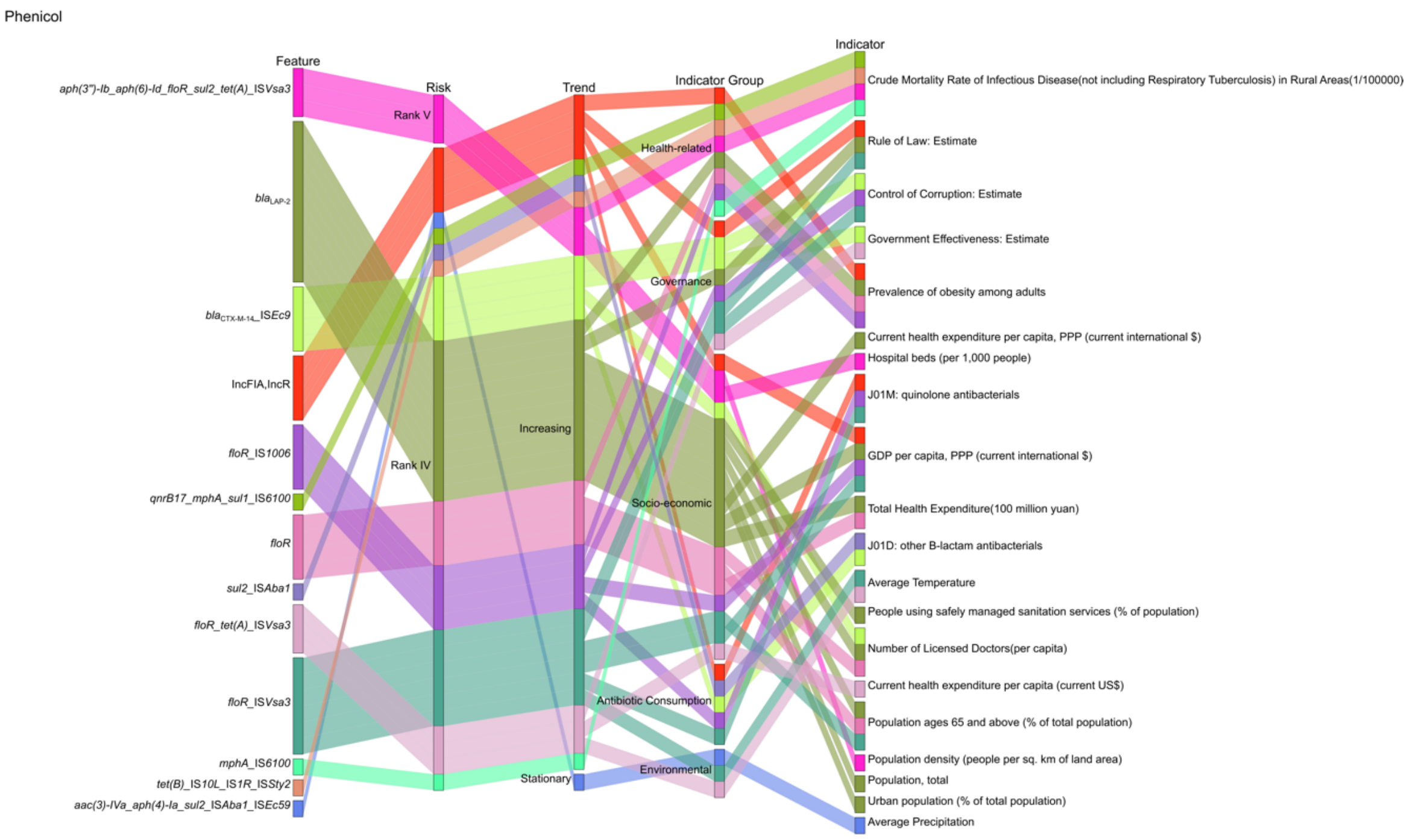


**Figure S29**. **Phenicol antibiotic model Sankey flow diagram.** This Sankey diagram illustrates the relationships between AMR genomic traits (AMR trait), their associated risk (Risk), the projected trend of each indicator (whether the indicator was forecast to increase or remain stationary), indicator groups, and specific indicators (Indicator) within each group. Only high-risk traits associated with increasing or stationary indicators are displayed. The thickness of the links represents the number of different indicators, while the colours correspond to the associated genomic trait. Source data are provided as a Source Data file.

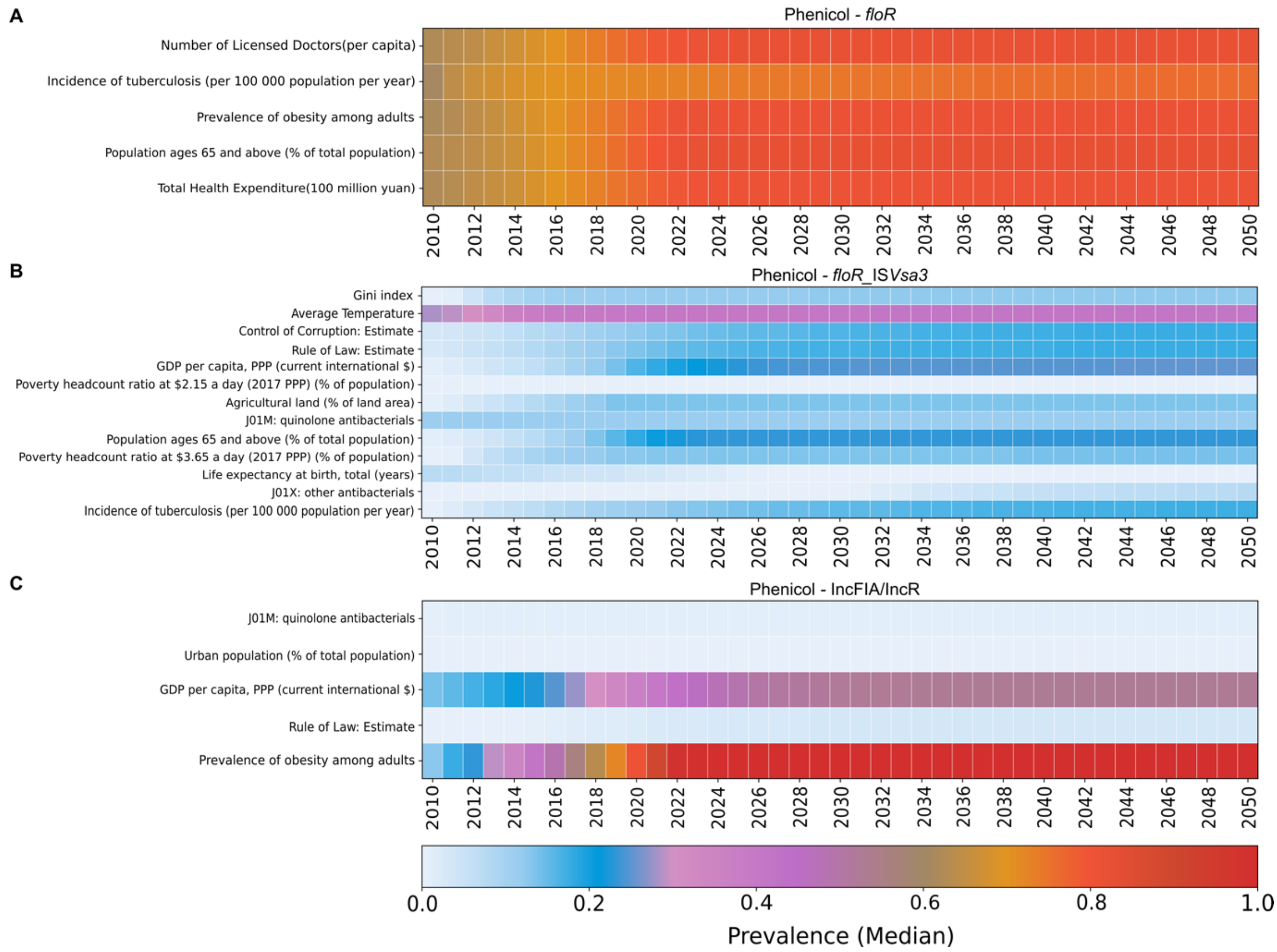


**Figure S30. Phenicol antibiotic model forecasting heatmap.** This heatmap illustrates the median value obtained by the Monte Carlo forecasts of the relative presence of the AMR traits (A) *floR*, (B) *floR*_IS*Vsa3*@IS*CR*@nan, and (C) IncFIA/IncRIS*Azba1* within phenicol resistant *S. enterica* to 2050 strongly correlated with increasing or stationary key determinants of health indicators. Median forecast of 10000 simulations is shown with a colour bar indicating the range of the feature prevalence rate between 0 and 1. Source data are provided as a Source Data file.

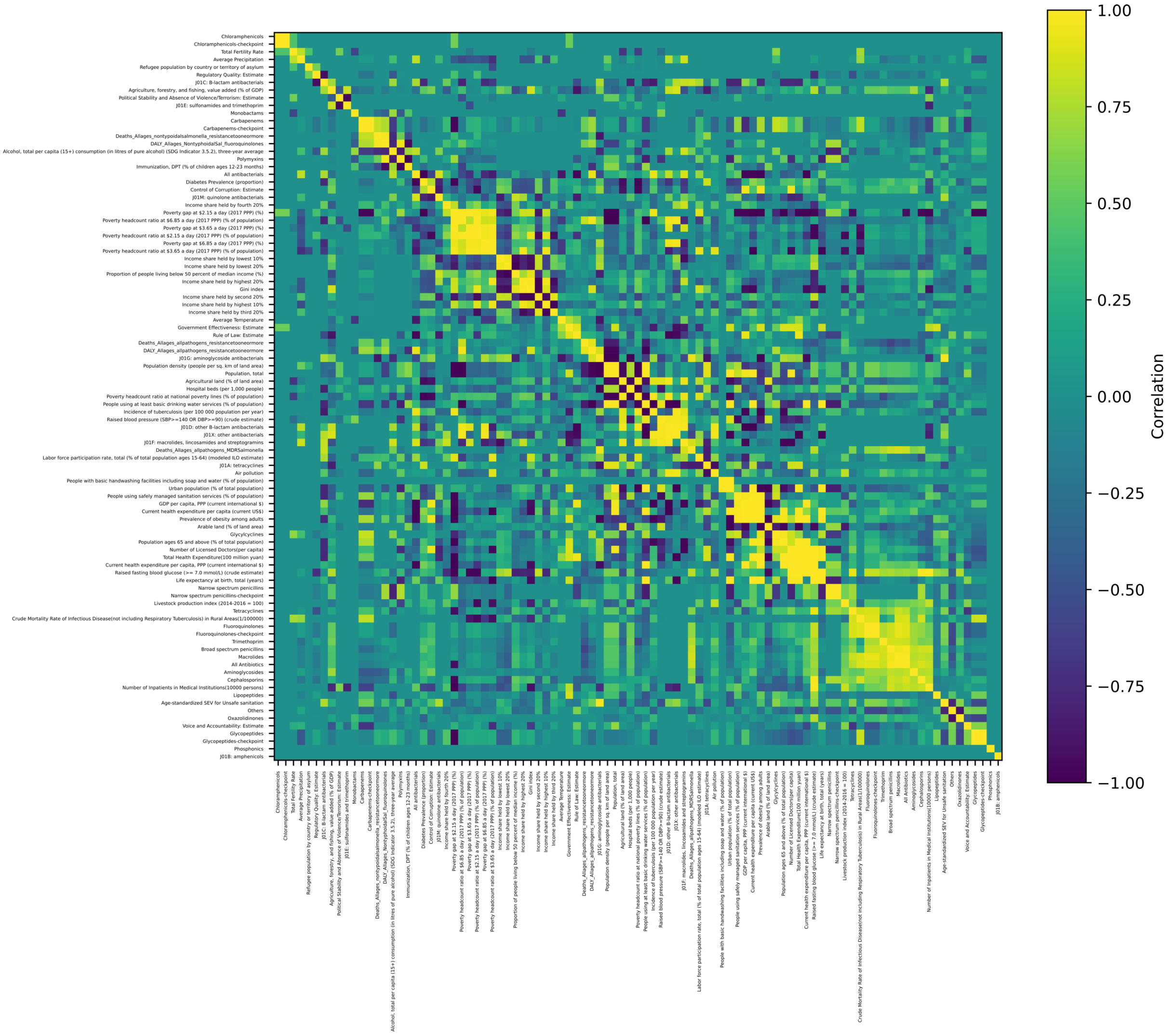


**Figure S31. Indicator-indicator correlation.** Clustered heatmap showing the amount of correlation between the indicators used in this study. Colours of the heatmap indicator the Pearson *r* value of each pair of indicators. Indicators were clustered based on |r| include positive and negative associations in the clustering. Source data are provided as a Source Data file.

Supplementary Results

*Comparison of MDR to non-MDR S. enterica genomic traits*

Comparing MDR isolates to non-MDR isolates, revealed that less AMR traits were distributed across all sources in non-MDR isolates (4.9% of traits), showing a significant difference from MDR isolates (adjusted p-value=0.0012, Fisher exact test, two-tailed). Across all source types, MDR isolates contained more ARGs per isolate than non-MDRs isolates (Mann Whitney U test, two-tailed, Bonferroni correction, p-values < 0.05). Similarly, MDR isolates carried more ARG-carrying ISs per isolate in all source types except non-food workers (Mann Whitney U test, two-tailed, Bonferroni correction, p-values < 0.05) **Fig. S8**. Additionally, MDR isolates from retail pork and chicken meat harboured more plasmids per isolate than non-MDR isolates (Mann Whitney U test, two-tailed, Bonferroni correction, p-values < 0.05) **Fig. S8**.

The non-MDR AMR genomic traits were encoded as features and the ML pipeline was used to associate the features to AMR phenotypes, following the same pipeline as that used for the MDR dataset (**Fig. S12, Table S3** and **Supplementary Data 11)**. Eighteen AMR traits were selected by the ML as being correlated to resistance. Of these, 17 were high risk (one Rank V and 16 Rank IV), and one was low risk (Rank II), **Table S3.** However, in contrast to the MDR data, no significant deviation between the risk distribution of non-MDR features and that of the broader population was found (chi-squared test, two-tailed, *p*-value=0.45),.

*Benchmarking of the forecasting of temporal trends against literature*

To benchmark the accuracy of our forecasts, we compared our predictions for two indicators (current health expenditure per capita and proportion of population over 65) with independent forecasts from the literature[1]. The results of our approach were compared and found to be broadly in line. Our projected health expenditure trend aligned closely with two prior forecasts published in 2019[1] and 2023, **Fig. S13**, both of which forecast current health expenditure out to 2035 and with the latter falling entirely within our prediction interval. The 2019 forecast projected higher expenditure than our forecast though followed similar trends, while for the most recent 2023 forecast our forecast was within the error bounds for all forecast points. For population aging, our estimates largely matched a 2009 projection by Li, Reuser [3], diverging at only one point, while UN projections[4] showed a steeper increase from 2028 onward.

*Details of genomic AMR traits forecast to increase in each antibiotic class linked to increasing/stationary trends*

*Aminoglycoside resistance traits*

For aminoglycoside-associated resistance, 27 traits (13 ARGs: aac(6')-Iy, aadA17, aadA22, aadA7, ant(2'')-Ia, bla$_{\text{CMY-59}}$, bla$_{\text{CTX-M-55}}$, bla$_{\text{DHA-1}}$, dfrA14, mphA, qnrB17, qnrB4, qnrB5; 13 ISs: aph(3'')-Ib_aph(6)-Id_floR_sul2_tet(A)_ISVsa3, aac(3)-IVa_aph(3'')-Ib_aph(3')-Ia_aph(4)-Ia_aph(6)-Id_ISEc59, aac(3)-IVa_aph(3')-Ia_aph(4)-Ia_ISEc59, aadA16_arr-3_dfrA27_mphA_sul1_IS6100, aph(3'')-Ib_aph(6)-Id_$_{\text{blaTEM-1}}$_sul2_IS5075, aph(3'')-Ib_aph(6)-Id_IS1133, aph(3'')-Ib_aph(6)-Id_sul2_tet(A)_IS26, bla$_{\text{CMY-59}}$_ISEc9, bla$_{\text{CTX-M-65}}$_IS903B, floR_ISVsa3, floR_tet(A)_ISVsa3, qnrS1_ISKpn19, tet(B)_IS10L_IS1R; and 1 plasmid: no rep_type) were linked to 21 increasing/stationary indicators including 14 socio-economic (e.g. income share of the lowest 20%, number of licensed doctors, people using safely managed sanitation services, number of inpatients in medical institutions, urban population and population density), 2 antibiotic consumption (tetracyclines, and beta-lactams), 3 health-related (diabetes prevalence, mortality of infectious disease, and prevalence of obesity among adults) and 2 governance (e.g. Government Effectiveness: Estimate), **Figs. S21 and S22.**

*β-lactam combination agent resistance traits*

For β-lactam combination agent-associated resistance, 22 traits (11 ARGs: aac(6')-Iaa, aadA16, aadA22, aph(3'')-Ib, bla$_{\text{CTX-M-14}}$, bla$_{\text{CTX-M-55}}$, bla$_{\text{CTX-M-65}}$, bla$_{\text{OXA-10}}$, bla$_{\text{TEM-1}}$, mphA, tet(D); 11 ISs: aac(3)-IVa_aph(3'')-Ib_aph(3')-Ia_aph(4)-Ia_aph(6)-Id_ISEc59, aac(3)-IVa_aph(4)-Ia_sul2_ISAba1_ISEc59, aadA16_arr-3_dfrA27_mphA_sul1_IS6100, aph(3'')-Ib_aph(6)-Id_bla$_{\text{TEM-1}}$_sul2_IS5075, bla$_{\text{CMY-59}}$_ISEc9, bla$_{\text{CTX-M-14}}$_ISEc9, bla$_{\text{CTX-M-55}}$_ISEc9, bla$_{\text{CTX-M-65}}$_IS903B, floR_IS1006, floR_IS1006_IS5075, tet(B)_IS10L) were linked to 19 increasing/stationary indicators including 10 socio-economic (e.g. number of licensed doctors, people using safely managed sanitation services, current healthcare expenditure, urban population and population density), 1 antibiotic consumption (e.g. quinolones), 2 environmental (temperature and precipitation), 3 health-related (mortality of infectious disease, diabetes prevalence and prevalence of obesity among adults) and 3 governance (government effectiveness, control of corruption, and rule of law), **Fig.S23**. The known β-lactam combination agent resistance genes bla$_{\text{CTX-M-14}}$, bla$_{\text{CTX-M-55}}$, bla$_{\text{CTX-M-65}}$, bla$_{\text{OXA-10}}$, bla$_{\text{TEM-1}}$were forecasted to increase in prevalence and positively correlated to 10 increasing indicators, **Fig. S24**.

*Folate resistance traits*

For folate-associated resistance, 11 traits (5 ARGs: *aadA16*, *aadA22*, *dfrA1*, *dfrA14*, *mphA*; and 6 ISs: *aph(3")-Ib_aph(6)-Id_floR_sul2_tet(A)*_IS*Vsa3,* *aac(6")-Ib_cr6_anrB17_aadA16_arr-3_dfrA27_sul1*_IS*6100,* *aadA16_arr-3_dfrA27_mphA_sul1*_IS*6100,* *dfrA1_floR_sul1_tet(A)*_IS*6100*_IS*Vsa3,* *floR_tet(A)*_IS*Vsa3,* *aac(6")-Ib10_aadA16_arr-3_dfrA17_mphA_sul1*_IS*6100)* were linked to 21 increasing/stationary indicators including 12 socio-economic (e.g. income share of the lowest 10%, basic drinking water access, number of inpatients in medical institutions, urban population and population), one antibiotic consumption (tetracyclines), 3 health-related (diabetes prevalence, mortality of infectious disease, and prevalence of obesity among adults) and four governance (e.g. government effectiveness and rule of law), **Fig. S25**. The known folate resistance genes *dfrA14* was forecast to increase in prevalence and positively correlated to 6 increasing indicators, while the folate resistant gene *dfrA12* was predicted to decrease, potentially reaching zero prevalence by 2040, **Fig. S26**.

*Macrolide resistance traits*

For macrolide-associated resistance, two traits, the ARGs *dfrA14* and *bla*$_{\text{LAP-2}}$ were linked to four increasing/stationary increasing/stationary indicators including three socio-economic (total health expenditure and number of licensed doctors, current health expenditure per capita and 1 governance (rule of law), **Figs. S27 and S28**.

*Phenicol resistance traits*

Thirteen phenicol-associated high risk traits(2 ARGs: *bla*$_{\text{LAP-2}}$ and *floR*; 1 plasmid: IncFIA/IncR; and 10 ISs: *aac(3)-IVa_aph(4)-Ia_sul2_ISAba1*_IS*Ec59*, *aph(3")-Ib_aph(6)-Id_floR_sul2_tet(A)*_IS*Vsa3*, *bla*$_{\text{CTX-M-14}}$_IS*Ec9*, *floR*_IS*1006*, *floR*_IS*Vsa3*, *floR_tet(A)*_IS*Vsa3*, *mphA*_IS*6100*, *qnrB17_mphA_sul1*_IS*6100*, *sul2*_IS*Aba1*, *tet(B)*_IS*10L*_IS*1R*_IS*Sty2*) were linked to 20 increasing or stationary indicators including 11 socio-economic (e.g. GDP, total health expenditure, people using safely managed sanitation services, hospital beds per 1000 people, and population density), 2 antibiotic consumption (e.g. beta lactams and), 2 health-related (incidence of infectious diease and prevalence of obesity among adults), 2 environmental (temperature and precipitation) and 3 governance (government effectiveness, control of corruption and rule of law), **Fig. S29**. The phenicol gene, *floR*, highly prevalent in historical data (83% relative prevalence in 2022), was also forecast to increase by 5 increasing indicators, **Fig. S30**. Concurrently, ARG-carrying ISs (*e.g. floR*_IS*Vsa3*), carrying *floR* were also forecast to increase in prevalence and was positively correlated to 12 increasing indicators.

**References**


1. Zhai T, Goss J, Dmytraczenko T, Zhang Y, Li J, Chai P. China's Health Expenditure Projections To 2035: Future Trajectory And The Estimated Impact Of Reforms. *Health Affairs* **38**, 835-843 (2019).

2. Sahoo PM, Rout HS, Jakovljevic M. Future health expenditure in the BRICS countries: a forecasting analysis for 2035. *Global Health* **19**, 49 (2023).

3. Li Q, Reuser M, Kraus C, Alho J. Ageing of a giant: a stochastic population forecast for China, 2006–2060. *Journal of Population Research* **26**, 21-50 (2009).

4. UN. United Nations - Department of Economic and Social Affairs Population Division. *World Population Prospects 2024* **Online Edition**, (2024).

5. People's Republic of China. National Bureau of Statistics of China (accessed 01-01-2025). (2025).
