# Supplementary material for: High-risk antimicrobial resistance genomic traits in multidrug-resistant Salmonella across China’s food chain: projections to 2050
Source: Nat Commun. 2026 Oct 20;17:10744. doi: 10.1038/s41467-026-78346-7 (PMC13683381; doi:10.1038/s41467-026-78346-7)
Supplement: Supplementary file 4 [file 41467_2026_78346_MOESM2_ESM.pdf]

Description of Additional Supplementary Files

File Name: Supplementary Data 1
Description: Metadata of genomes – List of isolates present in this study with their corresponding ID accession NCBI and in the National Genomics Data Center. For each isolate, the following information is given: sample ID, presence in MDR dataset or non-MDR dataset, database, Bioproject ID, sample accession ID, collection year, source type, symptom, serovar, ST type, serogroup, location province, AST phenotypes for: AMC (amoxicillin-clavulanate), AMK (amikacin), AMP (ampicillin), AZI (azithromycin), AZM (aztreonam), CAZ (ceftazidime), CFZ (cefazolin), CHL (chloramphenicol), CIP (ciprofloxacin), COL (colistin), CRO (ceftriaxone), CTX (cefotaxime), CXM (cefuroxime), CZA (ceftazidime-avibactam), DOX (doxycycline), ETP (ertapenem), FEP (cefepime), FOS (fosfomycin w/G6P), FOX (cefoxitin), GEN (gentamicin), IPM (imipenem), KAN (kanamycin), LVX (levofloxacin), MEM (meropenem), MIN (minocycline), MXF (moxifloxacin), NAL (nalidixic acid), NIT (nitrofurantoin), NOR (norfloxacin), SAM (ampicillin-sulbactam), STR (streptomycin), SUL (sulfisoxazole), SXT (trimethoprim-sulfamethoxazole), TET (tetracycline), TOB (tobramycin), TZP (piperacillin-tazobactam), XNL (ceftiofur).

File Name: Supplementary Data 2
Description: List of AMR traits and their genomic location based on the MDR S. enterica isolates, indicating the distribution of the AMR features location as a percentage (ARG located in the chromosome = ARGs-Chr, ARG located in the chromosome and integrated within an IS = ISs-ARGs, ARG located in the plasmid and integrated within an IS = ISs-Plasmid-ARG and ARGs present on plasmids but not within an insertion sequence = Plasmid-ARGs) across all MDR S. enterica isolates.

File Name: Supplementary Data 3
Description: List of AMR traits, their types, genomic locations and their source location based on the MDR S. enterica isolates, indicating for each of the 7 source types (chicken (farms), environment (farms), food workers, non-food workers, retail pork meat, retail chicken meat, symptomatic humans) the corresponding number of isolates where the AMR trait is present.

File Name: Supplementary Data 4
Description: Risk Assessment. List of AMR traits, types, genomic location and their corresponding risk assessment including the risk category and scores for both risk severity and risk likelihood.

File Name: Supplementary Data 5
Description: GLMM performance and ICCs for the MDR S. enterica isolates correlating the genetic features to the resistance/susceptibility against a panel of antimicrobials. The performance metrics were AUC, PR-AUC, accuracy (TP+TN/(P+N)), sensitivity (true positive rate: TP/P), specificity (true negative rate: TN/N), AUC and precision a. The scores for each performance metric were computed from 50 simulations using groups K-fold cross-validation. The mean ± standard deviation of these 50 iterations was then used as the result statistics for the performance.

File Name: Supplementary Data 6

Description: AMR traits selected by the GLM models with an OR ≥ 2, a lower 95% OR confidence interval ≥ 1 and OR ≥ 1 in at least 80% of CV trials. Shown is the antibiotic model in which the trait was selected, the antibiotic class associated with the antibiotic, the AMR trait, the traits rank the risk assessment, the traits coefficient, OR, and upper and lower 95% confidence intervals.

File Name: Supplementary Data 7

Description: GLMM performance and ICCs for the non-MDR S. enterica isolates correlating the genetic features to the resistance/susceptibility against a panel of antimicrobials. The performance metrics were AUC, PR-AUC, accuracy (TP+TN/(P+N)), sensitivity (true positive rate: TP/P), specificity (true negative rate: TN/N), AUC and precision a. The scores for each performance metric were computed from 50 simulations using groups K-fold cross-validation. The mean ± standard deviation of these 50 iterations was then used as the result statistics for the performance.

File Name: Supplementary Data 8

Description: Within-group external validation predictive performance metrics. Predictive performance for the within-group dataset, using coefficient estimated from the fitted training dataset (pre 2021 data) *S. enterica* models. The performance metrics were AUC, PR-AUC, accuracy (TP+TN/(P+N)), sensitivity (true positive rate: TP/P), specificity (true negative rate: TN/N), AUC and precision. Also given is the training data set AUC mean and standard deviation; the full model AUC mean and standard deviation, the antibiotic specific number of isolates included in the training and within-group validation datasets (after intermediate phenotypes and missing phenotypes were removed); and the proportion of resistant isolates (R/(R+S)) in the training and within-group validation datasets. For KAN, the within-group validation set contained no susceptible isolates (100% resistant, n=39); specificity and AUC are therefore undefined and reported as blank cells, and the corresponding accuracy, precision, PR-AUC and sensitivity values should be interpreted accordingly.

File Name: Supplementary Data 9

Description: Out-of-group external validation predictive performance metrics. Predictive performance for the out-of-group dataset, using coefficient estimated from the fitted MDR S. enterica models. The performance metrics were AUC, PR-AUC, accuracy (TP+TN/(P+N)), sensitivity (true positive rate: TP/P), specificity (true negative rate: TN/N), AUC and precision. Also given is the full model AUC mean and standard deviation, the antibiotic specific number of isolates included in the training (original MDR dataset) dataset and out-of-group validation datasets (after intermediate phenotypes and missing phenotypes were removed); and the proportion of resistant isolates (R/(R+S)) in the training and out-of-group-group validation datasets.

File Name: Supplementary Data 10

Description: List of indicators from World Bank Data, ResistanceMap, Meteostat data, Wushouer et al, National Bureau of Statistics of China and Global Health Data Exchange including governance, antibiotic consumption, social-economical, deaths, health-related and environmental risk factors collected from China based on the same years as the genomics data on Supplementary Data 1.

File Name: Supplementary Data 11
Description: Province-level GDP per capita for the 15 Chinese provinces represented in the study dataset, 2006–2022. Data extracted from the National Bureau of Statistics of China Statistical Yearbook[5]. The coefficient of variation (CV) across provinces is reported for each year to quantify the degree of geographic heterogeneity in this socioeconomic indicator over the study period. National level GDP per capita is also included for comparison but not included in the CV calculation.

File Name: Supplementary Data 12
Description: Pearson correlation coefficients and p-value statistics for each indicator-trait pair (Pearson correlation $r > |0.5|$).

File Name: Supplementary Data 13
Description: List of trait-indicator pairs strongly correlated (Pearson correlation $r > |0.5|$) with the indicator and forecasted to increase/decrease/remain stationary by 2050. For each trait-indicator pair the following information is given: risk category, the number of isolates and years used for the forecasting analysis; the trend analysis performed for both the indicator and the feature forecasting using ADF (Augmented Dickey-Fuller) and KPSS (Kwiatkowski-Phillips-Schmidt-Shin) test statistics with a confidence level of 95% for each one; the number of locations the trait was found in, a list of the locations, whether the trait is found in more than 1 location, the number of serogroups the trait was found in, a list of the serogroups, whether the trait was found in more than 1 serogroup, the number of classes related to the antibiotic GLM models selecting the trait, a list of the antibiotic classes, whether the trait was associated to more than 2 classes in the GLM model (considered MDR).
